# Design and Optimization of Thioglycosyl–naphthalimides as Efficient Inhibitors Against Human O-GlcNAcase

**DOI:** 10.3389/fchem.2019.00533

**Published:** 2019-07-25

**Authors:** Shengqiang Shen, Lili Dong, Wei Chen, Renjie Wu, Huizhe Lu, Qing Yang, Jianjun Zhang

**Affiliations:** ^1^Department of Applied Chemistry, College of Science, China Agricultural University, Beijing, China; ^2^Institute of Plant Protection, Chinese Academy of Agricultural Sciences, Beijing, China

**Keywords:** thioglycosyl-naphthalimides, ureido glycosides, β-N-acetylhexosaminidases, human O-GlcNAcase, inhibitors, molecular docking and MD simulations

## Abstract

β-N-acetylhexosaminidases represent an important class of exoglycosidases and have emerged as the promising targets for drug and pesticide discovery. Among these, human O-GlcNAcase (hOGA) has been reported to be closely linked to several diseases such as Alzheimer's disease, diabetes, and cancer. Potent hOGA inhibitors with high selectivity are therefore of great significance for the regulation of the corresponding physiological processes. In this study, several classes of novel and readily available thioglycosyl-naphthalimides bearing the amide linker were designed and synthesized. To investigate their potency and selectivity, the inhibitory efficiencies toward hOGA and human β-N-acetylhexosaminidase B (HsHexB) were assayed. Especially, compounds **10a** (*K*_i_ = 0.61 μM) and **16l** (*K*_i_ = 0.72 μM) exhibited excellent inhibitory potency against hOGA and high selectivity (HsHexB, *K*_i_ > 100 μM). In addition, during the preparation of these thioglycosyl–naphthalimides, a new practical method was developed for the synthesis of ureido glycosides from trichloroethyl carbamates at room temperature and normal pressure without catalyst. Furthermore, the possible binding modes of hOGA with **10a**, **10d**, and **16j** were studied using molecular docking and molecular dynamics simulations to explore the molecular basis for the potency of these thioglycosides. This work present here provides useful clues for the further structural optimization toward hOGA.

## Introduction

β-N-acetylhexosaminidases (EC 3.2.1.52) are responsible for catalyzing the hydrolysis of β-linked N-acetyl-D-hexosamine unit from non-reducing ends of glycans, glycoproteins, and glycolipids (Henrissat and Davies, 1997; Liu et al., 2012). Based on the Carbohydrate Active Enzymes database (CAZy, http://www.cazy.org/), most β-N-acetylhexosaminidases are classified into three glycosyl hydrolase families, namely, GH3, GH20, and GH84 (Henrissat and Davies, 1997; Cantarel et al., 2009). These enzymes are widely distributed in many organisms and are involved in various physiological functions (Intra et al., 2008; Liu et al., 2012). Remarkably, GH84 human O-GlcNAcase (hOGA) has drawn significant attention and has been considered as a potential target for drug development (Bergeron-Brlek et al., 2015; Kong et al., 2016; Roth et al., 2017; Shen et al., 2018a).

GH84 human O-GlcNAcase (hOGA) is a 103-kDa multidomain protein that can catalyze the removal of β-linked N-acetyl-D-glucosamine (GlcNAc) from serine and threonine residues (Elsen et al., 2017). The hOGA combined with human O-GlcNAc transferase (hOGT) regulate the dynamic removal and addition of O-GlcNAc for hundreds of nucleocytoplasmic proteins (Yuzwa et al., 2008; Elsen et al., 2017). This modification has also been reported to compete with protein phosphorylation (Yuzwa et al., 2008). Perturbation of O-GlcNAc cycling has been implicated in many diseases such as Alzheimer's disease (Yuzwa and Vocadlo, 2014), type II diabetes (McClain et al., 2002), and cancer (Ferrer et al., 2014). Since they share the same substrate-assisted catalytic mechanism, GH20 human β-N-acetylhexosaminidase (HsHex) can hydrolyze the terminal non-reducing GalNAc from GM2 ganglioside (Mahuran, 1999). Dysfunction of HsHex results in GM2 ganglioside storage disorders in the lysosome, which leads to Tay–Sachs and Sandhoff disease (Mahuran, 1999).

**Figure 1:**
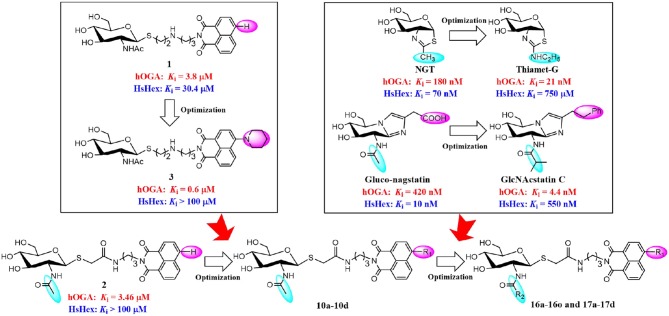
Optimization strategy of thioglycosyl–naphthalimides toward hOGA.

Potent hOGA inhibitors with good selectivity (over HsHex) are greatly important to study the physiological functions of hOGA (Yuzwa et al., 2008; Dorfmueller et al., 2009). To date, a number of hOGA inhibitors have been reported, such as PUGNAc (Macauley et al., 2005), NAG-thiazoline (NGT) (Krejzova et al., 2014), Streptozotocin (Toleman et al., 2006), Nagstatin (Aoyagi et al., 1992; Terinek and Vasella, 2005), iminocyclitols (Bergeron-Brlek et al., 2015), and thioglycosyl–naphthalimides (Chen et al., 2018; Shen et al., 2018a,b). Among these inhibitors, NGT is synthesized as an analog of NAG-oxaoline (which is a reaction intermediate of the substrate-assisted mechanism) (Knapp et al., 1996) with low selectivity toward hOGA (*K*_i_ = 180 nM) (Krejzova et al., 2014) and HsHex (*K*_i_ = 70 nM) (Knapp et al., 1996). To improve the selective inhibition against hOGA, Thiamet-G bearing an ethylamino substituent on the thiazoline ring was designed and found to exhibit excellent potency (*K*_i_ = 21 nM against hOGA) and selectivity (*K*_i_ = 750 μM against HsHex) (Yuzwa et al., 2008) (Figure 1). This modification indicates that hOGA possesses a larger catalytic active pocket than HsHex, and the appropriate increase of the methyl group of thiazoline could improve the binding affinity toward hOGA. The potent β-N-acetylhexosaminidase inhibitor Nagstatin, was originally isolated from fermentation broth of *Streptomyces amakusaensis* (Aoyagi et al., 1992). Subsequently, Gluco-nagstatin was reported by Terinek with *K*_i_ values of 420 nM against hOGA and 10 nM against HsHex (Terinek and Vasella, 2005). Furthermore, GlcNAcstatin C, bearing the 2-isobutyramido on the glycosyl moiety and phenemyl on the iminazole group was found to be an extremely potent hOGA inhibititor (*K*_i_ = 4.4 nM) with 164-fold selectivity compared to HsHex (*K*_i_ = 550 nM) (Dorfmueller et al., 2009) (Figure 1). These results suggest that enlargement of the substituent at the 2-position of the glycosyl moiety of Nagstatin leads to an increase of both the inhibitory efficiency and selectivity toward hOGA. These classical inhibitors exhibited significant potency against hOGA; however, further applications may be limited by the large-scale preparation (complex synthetic methods) and drug-like properties (Dorfmueller et al., 2009; Krejzova et al., 2014).

In our previous research, we presented thioglycosyl–naphthalimides **15b** (**1**, *K*_i_ = 3.8 μM against hOGA, *K*_i_ = 30.4 μM against HsHexB) and **5c** (**2**, *K*_i_ = 3.46 μM against hOGA, *K*_i_ > 100 μM against HsHexB) as promising lead compounds for hOGA (Chen et al., 2018; Shen et al., 2018a). Subsequently, modification of the 4-substituent group at the naphthalimide of **15b** led to the synthesis of **13r** (**3**, *K*_i_ = 0.6 μM against hOGA; *K*_i_ >100 μM against HsHexB), bearing a 4-piperidylnaphthalimide moiety, which exhibited higher potency and selectivity toward hOGA (Shen et al., 2018b) (Figure 1). This indicated that the 4-substituent at the naphthalimide scaffold critically affects the activity and selectivity of thioglycosyl–naphthalimides against hOGA (Shen et al., 2018b). Considering the important functions of hOGA for the post-translational modification within eukaryotic cells, this study further attempted to optimized the structure of **5c** (**2**) to continue to increase the efficiency and selectivity against hOGA. The utilized strategy first synthesized 4-substituted naphthalimide thioglycosides and evaluated their inhibitory activities against hOGA and HsHexB. Thus, suitable 4-substituents (on the naphthalimide moiety) were identified through structure-activity relationship studies. Then, we focused on the optimization of the 2-acetamido group at the glycosyl moiety to obtain novel thioglycosyl–naphthalimides hOGA inhibitors with increased potency and selectivity (Figure 1). Accordingly, several classes of thioglycosyl–naphthalimides bearing amide linkers were synthesized and their inhibitory activities against hOGA and HsHexB were evaluated.

## Results and Discussion

### Synthesis of Thioglycosyl–naphthalimides 10a-10d

**Scheme 1:**
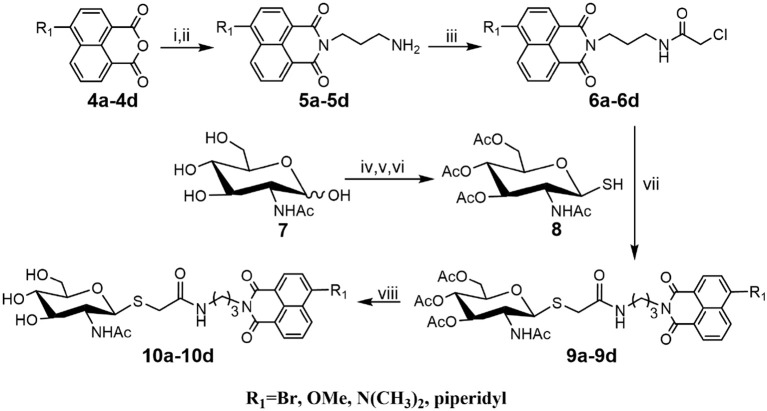
Synthesis of thioglycosyl–naphthalimides **10a-10d**. (i) *tert*-butyl (3-aminopropyl) carbamate, EtOH; (ii) DCM, CF_3_COOH; (iii) 2-chloroacetyl chloride, Et_3_N, DCM; (iv) AcCl; (v) thiourea, acetone; (vi) Na_2_S_2_O_5_, DCM, H_2_O; (vii) K_2_CO_3_, acetone, H_2_O; (viii) CH_3_NH_2_, MeOH. R_1_ is defined in Table 1.

As shown in Scheme 1, compounds **5a-5d** were prepared according to literature methods (Shen et al., 2018b) and reacted with chloracetyl chloride to afford **6a-6d**. Meanwhile, the key intermediate **8** was obtained from N-acetyl-D-glucosamine as previously described (Paul and Korytnyk, 1984). Then, treatment of **6a-6d** with thiol **8** in 2:1 acetone/H_2_O mixture yielded precursors **9a-9d**. Finally, deacetylation of **9a-9d** via methylamine catalysis formed the target products **10a-10d** (Scheme S1).

### Inhibitory Activities of 10a-10d Against hOGA and HsHexB

Inhibitory activities of target compounds **10a-10d** against hOGA and HsHexB were evaluated *in vitro* (Table 1). The results showed that the 4-substituted group on the naphthalimide moiety significantly influenced the potency of thioglycosyl–naphthalimides against hOGA. Specifically, thioglycosyl–naphthalimides bearing OMe (**10b**) and N(CH_3_)_2_ (**10c**) substituents exhibited an activity decrease compared to that of naphthalimides bearing no 4-substituents (lead compound **2**). Thioglycosides bearing the 4-bromo (**10a**) and 4-piperidyl (**10d**) on the naphthalimide moiety improved the inhibitory potency. Compound **10a** in particular exhibited the highest inhibitory efficiency against hOGA with a *K*_i_ value of 0.61 μM (indicating an almost 6-fold increase in potency) and excellent selectivity (*K*_i_ >100 μM against HsHexB).

**Table 1:** Inhibitory activities of **10a-10d** against hOGA and HsHexB.

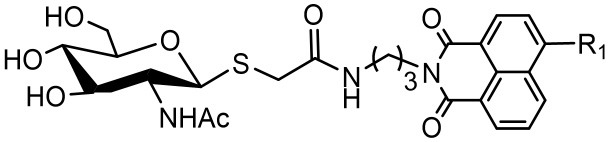					
**Compd**	**R**_**1**_	**Inhibition rate at 100** **μM (%)**		***K***_**i**_ **(μM)**	
		**hOGA**	**HsHexB**	**hOGA**	**HsHexB**
**10a**	Br	98.7 ± 0.3	36.5 ± 0.8	**0.61** **±** **0.02**	**>100**
**10b**	OMe	90.6 ± 0.5	49.2 ± 1.4	4.43 ± 0.21	>100
**10c**	N(CH_3_)_2_	84.4 ± 1.7	36.9 ± 0.2	9.72 ± 0.49	>100
**10d**	Piperidyl	93.1 ± 0.2	32.8 ± 2.7	**1.42** **±** **0.02**	**>100**
**2**	H	97.6 ± 0.9	45.5 ± 0.5	3.46 ± 0.11	>100

*Bold values indicate bioassay data of compounds with better activity*.

### Optimization of Inhibitors 10a and 10d

**Scheme 2:**
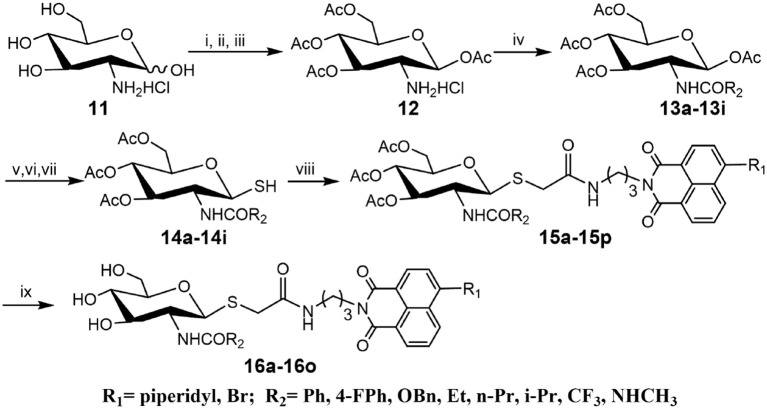
Synthesis of thioglycosyl–naphthalimides **16a-16o**. (i) *p*-anisaladehyde, NaOH, H_2_O; (ii) Py, Ac_2_O; (iii) acetone, HCl, H_2_O; (iv) Et_3_N, CH_2_Cl_2_, R_2_COCl for **13a-13h**; Et_3_N, CH_2_Cl_2_, TFAA for **13i**; (v) HBr, CH_3_COOH, CH_2_Cl_2_; (vi) thiourea, acetone; (vii) Na_2_S_2_O_5_, CH_2_Cl_2_, H_2_O; (viii) K_2_CO_3_, acetone, H_2_O, **6a** or **6d**; (viii) CH_3_NH_2_, MeOH. R_1_ and R_2_ are defined in Table 2.

Compounds with higher inhibitory potency toward hOGA (**10a** and **10d**), were selected for further structural optimization. Considering that hOGA has a larger catalytic pocket than HsHex and can accommodate more bulky groups near the 2-acetamide of related substrates (Dorfmueller et al., 2009; Igual et al., 2019), we then focused on investigating the effect of 2-substituents at the glycosyl moiety on the inhibitory activity of hOGA. Accordingly, the Ph, 4-FPh, OBn, Et, *n*-Pr, *i*-Pr, CF_3_, and NHCH_3_ substituents were introduced into the position of the methyl group of 2-acetamido. The synthetic routes to thioglycosyl–naphthalimides **16a-16o** have been outlined in Scheme 2, and the specific synthesis methods are shown in Scheme S2.

### Inhibitory Activities of 16a-16o Against hOGA and HsHexB

Assays of the inhibitory activities of **16a-16o** against hOGA and HsHexB are presented in Table 2. Enlarging the size of the methyl group of 2-acetamido led to a decrease in inhibitory activity against hOGA, and larger substituents led to lower efficiency. Briefly, slightly increasing the size of the Me group (**10d**) to Et (**16d**) resulted in a minor decrease in inhibitory potency. Modification of Me to CF_3_, NHCH_3_, *n*-Pr, and *i*-Pr reduced the activity to a greater degree (from **10d** to **16g**, **16h**, **16e**, and **16f**). Upon increasing the size from Me (**10d**) to Ph (**16a**), 4-FPh (**16b**), and OBn (**16c**) led to the loss of activity against hOGA. Moreover, the 4-bromo group on the naphthalimide moiety of these thioglycosides was more beneficial than 4-piperidyl for increasing the inhibitory activity toward hOGA (compared to **16l-16o** and **16d-16g**). All target compounds **16a-16o** exhibited low inhibitory activity against HsHexB (*K*_i_ >100 μM), which indicates that these thioglycosides possess promising selectivity toward hOGA. Among **16a-16o**, compound **16l** showed the highest potency against hOGA with a *K*_i_ value of 0.72 μM.

**Table 2:** Inhibitory activities of **16a-16o** against hOGA and HsHexB.

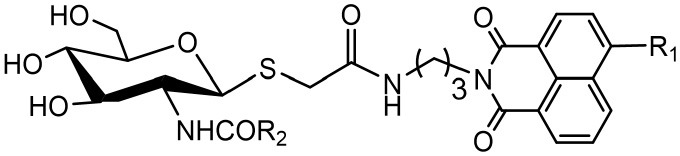						
**Compd**	**Substituent group**		**Inhibition rate at 100** **μM (%)**		***K***_**i**_ **(μM)**	
	**R**_**1**_	**R**_**2**_	**hOGA**	**HsHexB**	**hOGA**	**HsHexB**
**16a**	Piperidyl	Ph	22.0 ± 0.5	12.2 ± 0.2	>100	>100
**16b**	Piperidyl	4-FPh	50.9 ± 0.8	15.8 ± 0.4	>100	>100
**16c**	Piperidyl	OBn	9.7 ± 0.7	2.0 ± 1.1	>100	>100
**16d**	Piperidyl	Et	88.0 ± 1.5	28.7 ± 2.0	**1.69** **±** **0.05**	**>100**
**16e**	Piperidyl	n-Pr	78.9 ± 1.9	24.6 ± 1.5	12.80 ± 0.27	>100
**16f**	Piperidyl	i-Pr	72.2 ± 0.3	21.4 ± 0.5	23.58 ± 0.43	>100
**16g**	Piperidyl	CF_3_	82.7 ± 1.0	6.4 ± 0.7	6.37 ± 0.09	>100
**16h**	Piperidyl	NHCH_3_	85.5 ± 1.5	25.2 ± 0.3	6.60 ± 0.22	>100
**16j**	Br	Ph	41.1 ± 0.6	49.1 ± 1.4	>100	>100
**16k**	Br	4-FPh	48.0 ± 0.9	48.5 ± 1.7	>100	>100
**16l**	Br	Et	97.7 ± 2.2	41.8 ± 1.0	**0.72** **±** **0.01**	**>100**
**16m**	Br	n-Pr	97.1 ± 1.3	39.8 ± 2.3	4.36 ± 0.21	>100
**16n**	Br	i-Pr	87.8 ± 0.4	37.0 ± 0.4	6.92 ± 0.10	>100
**16o**	Br	CF_3_	90.9 ± 0.2	37.7 ± 0.9	4.62 ± 0.18	>100
**10d**	Piperidyl	Me	93.1 ± 0.2	32.8 ± 2.7	**1.42** **±** **0.02**	**>100**
**10a**	Br	Me	98.7 ± 0.3	36.5 ± 0.8	**0.61** **±** **0.02**	**>100**

*Bold values indicate bioassay data of compounds with better activity*.

### New Method for the Synthesis of Thioglycosyl–naphthalimide 16h

**Scheme 3:**
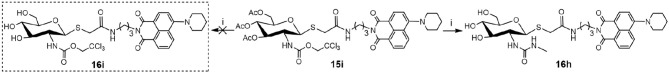
New method for the synthesis of thioglycosyl–naphthalimides **16h** (i) CH_3_NH_2_, MeOH.

Deacetylation of **15i**, bearing trichloroethoxycarbonyl (Troc) group, with methylamine catalysis at room temperature for 48 h did not obtain **16i**. Instead, thioglycoside **16h** bearing methylaminocarbonyl formed (Scheme 3). This suggests that the mechanism for this reaction is that methylamine nucleophilic attacks the isothiocyanate intermediate (yielded by the well-known base-promoted elimination of trichloroethanol) to produce the corresponding urea compounds (Azad et al., 2006). Review of previous literature, we found that the occurrence of this reaction required high pressure (Azad et al., 2006), high temperature (Jeong et al., 2016), or catalyst (Simard et al., 2009; Kono et al., 2013; Jang and Kim, 2017; Zhang et al., 2018) conditions. Accordingly, a new and more practical approach for the synthesis of ureido glycosides at room temperature and normal pressure without catalyst is reported here.

### Synthesis of Ureido Glycosides 17a-17d and Their Activities Against hOGA and HsHexB

**Scheme 4:**
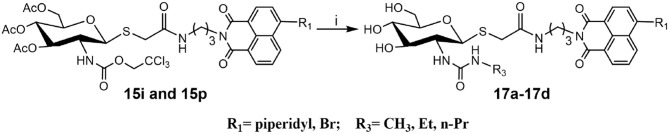
Synthesis of ureido glycosides **17a-17d**. (i) R_3_NH_2_, MeOH. R_1_, and R_3_ are defined in Table 3.

To verify the universality and reliability of this method, acetyl-protected precursors **15i** and **15p** were reacted with different amines (Scheme 4). Successfully, four new ureido glycosides (**17a-17d**) were synthesized through this reaction route. The specific synthesis methods can be found in Scheme S4 and the substituents R_1_ and R_3_ are defined in Table 3.

**Table 3:** Inhibitory activities of **17a-17d** against hOGA and HsHexB.

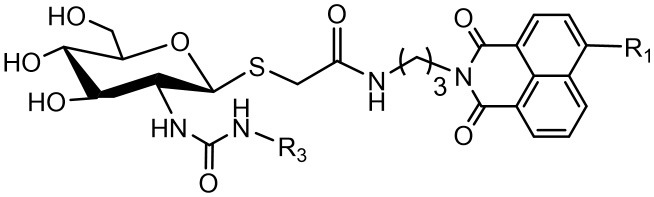						
**Compd**	**Substituent group**		**Inhibition rate at 100** **μM (%)**		***K***_**i**_ **(μM)**	
	**R**_**1**_	**R**_**3**_	**hOGA**	**HsHexB**	**hOGA**	**HsHexB**
**17a**	Piperidyl	Et	68.6 ± 0.8	25.9 ± 0.3	12.01 ± 0.30	>100
**17b**	Piperidyl	n-Pr	66.3 ± 0.6	22.7 ± 0.2	13.83 ± 0.02	>100
**17c**	Br	Me	96.7 ± 1.6	40.7 ± 0.7	**2.17** **±** **0.13**	**>100**
**17d**	Br	Et	83.5 ± 0.9	26.3 ± 1.1	7.11 ± 0.01	>100
**16h**	Piperidyl	Me	85.5 ± 1.5	25.2 ± 0.3	6.60 ± 0.22	>100

*Bold values indicate bioassay data of compounds with better activity*.

Analysis of **17a-17d** against hOGA indicated that the inhibitory potency of these compounds was associated with the size of 2-position substituents on the glycosyl moiety. In detail, enlargement of the size of the NHCH_3_ (**16h** or **17c**) to NHC_2_H_5_ (**17a** or **17d**) resulted in a 2 to 3-fold decrease in inhibitory activity. Continuing to increase of the size of the substituents from NHC_2_H_5_ (**17a**) to NHC_3_H_7_ (**17b**) showed a minor activity loss (the *K*_i_ value increased from 12.01 μM to 13.83 μM). Further comparison of **16d** (bearing CH_2_CH_3_) and **16h** (bearing NHCH_3_) showed that the basic substituent at the 2-position of the glycosyl moiety decreased the potency against hOGA (reduced by about 4-fold), which suggests that the 2-ureido group on the glycosyl moiety was not beneficial for the improvement of the activity of thioglycosyl-naphthalimides toward hOGA. Additionally, the presence of the 4-bromo group on the naphthalimide moiety also demonstrated a more effective potency than 4-piperidyl in these ureido glycosides **17a-17d**.

### Inhibitory Mechanism of the Thioglycosyl–naphthalimides Toward hOGA

**Figure 2:**
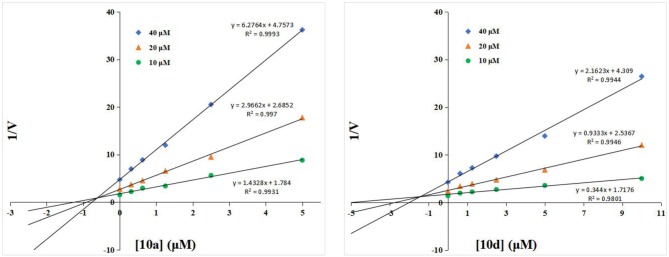
Dixon plots for compounds **10a** and **10d** against hOGA.

To understand the inhibitory mechanism of these thioglycosides toward hOGA, the highly potent inhibitors **10a** and **10d** were selected for kinetic studies via Dixon plots. As shown in Figure 2, the trendlines drawn for each concentration of substrate meet in quadrant two, suggesting that thioglycosides **10a** and **10d** are competitive inhibitors of hOGA.

**Figure 3:**
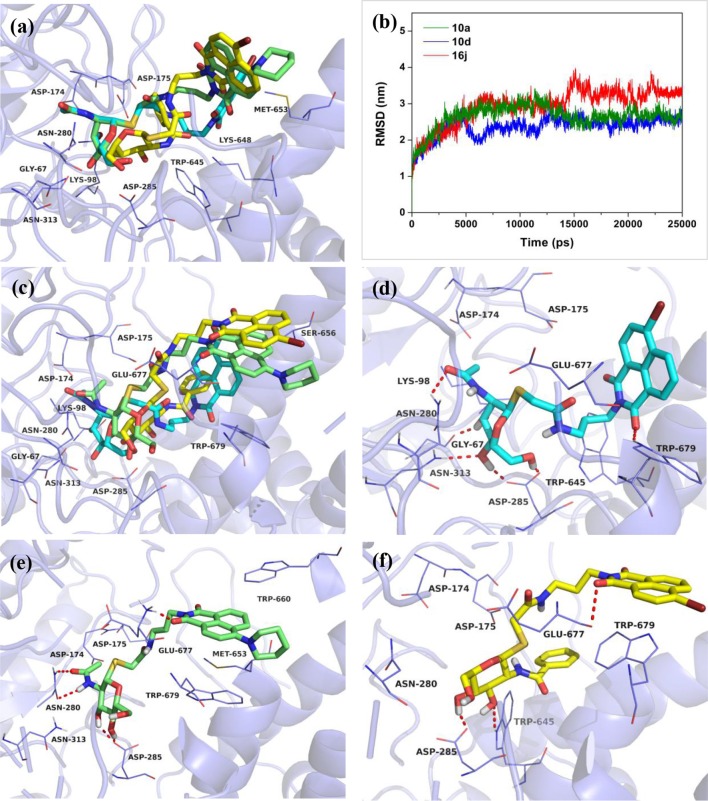
Predicted binding mechanisms of **10a**, **10d** and **16j** with hOGA calculated by molecular docking and MD simulations. Superimposition of the conformations of **10a**, **10d**, and **16j** with hOGA at **(a)** molecular docking and **(c)** 25 ns MD simulations. **(b)** RMSD changes of the three simulated systems (hOGA in complex with **10a**, **10d**, and **16j**). Specific binding patterns of **(d)** hOGA-**10a**, **(e)** hOGA-**10d**, and **(f)** hOGA-**16j** systems after 25-ns MD simulations. Compound **10a** is shown in cyan, **10d** is shown in green, **16j** is shown in yellow (colored by element).

Then, compounds **10a**, **10d**, and **16j** were used to investigate the basis for the potency of these thioglycosides against hOGA. Superimposition of the conformations of **10a**, **10d**, and **16j** with hOGA at molecular docking are shown in Figure 3a. The glycosyl moiety from these three compounds was bound into the active pocket of hOGA, and the conformation of glycosyl in **10d** was similar to that of **10a**. Compound **16j** showed a shallower binding in the active pocket compared to **10a**. Moreover, the naphthalimide group of **10a** was found to be closer to the active pocket than both **10d** and **16j**.

To shed further light on the appropriate binding patterns of **10a**, **10d** and **16j** with hOGA, 25 ns of MD simulations were implemented. As shown in Figure 3b and Figure S1a, the root-mean-square deviations (RMSDs) values of these three systems were maintained at around 1.9–3.4 Å, and the dynamic convergences were all achieved after 22 ns of simulations. These results suggested that the systems underwent reasonable conformational change.

The conformations of **10a**, **10d**, and **16j** in complex with hOGA at 25 ns of MD simulations are displayed in Figures 3c–f. As shown in Figure 3c, the glycosyl moiety of **10a** entered more deeply into the hOGA pocket compared to **10d**, and the glycosyl of **16j** only bound at the entrance of the active pocket (shallower than **10d**). These results are consistent with the inhibitory potency of these three compounds against hOGA (Tables 1, 2), and provide a preliminary explanation of the basis for their difference in activity.

Figures 3d–f show the specific binding patterns of **10a**, **10d**, and **16j** with hOGA. The glycosyl moiety of **10a** was found to be tightly bound to the active pocket via H-bonding interactions with residues Gly67, Asn280, Asp285, Asn313, and the oxygen of naphthalimide formed a hydrogen bond with Trp679 (Figure 3d). Moreover, the naphthalimide group of **10a** established van der Waals interactions with Glu677 and π-π stacking interactions with Trp679 (Figure 3d). As a comparison, affected by the larger 4-piperidyl group on the naphthalimide, the binding affinity of **10d** has a specific degree of reduction (Figure 3e). In particular, the glycosyl moiety of **10d** formed four hydrogen bonds (< **10a**, which possessed five hydrogen bonds) with residues Asn280, Asp285. Furthermore, the naphthalimide of **10d** formed a H-bonding interaction with Glu677 and π-π stacking interactions with Trp660 and Trp 679 (Figure 3e). These binding interactions may have resulted in the 2.3-fold decrease in inhibitory potency compared to **10a** (*K*_i_ = 0.61 μM) and **10d** (*K*_i_ = 1.42 μM). The large benzamido group of **16j** led to the inability of the glycosyl moiety to bind well at the hOGA active pocket, thus only forming two hydrogen bonds with Asp285 and Trp645. This result may explain the basis for the loss of inhibitory activity of **16j**.

## Conclusion

In summary, a series of novel and readily available thioglycosyl–naphthalimides derivatives were designed and synthesized as effective hOGA inhibitors. Remarkably, thioglycosides **10a** (*K*_i_ = 0.61 μM) and **16l** (*K*_i_ = 0.72 μM) displayed higher inhibitory potencies toward hOGA and exhibited excellent selectivity over HsHexB (Ki > 100 μM). Moreover, a new method for the synthesis of ureido glycosides was obtained by reaction of trichloroethyl carbamates and primary amines under room temperature and normal pressure without catalyst. Furthermore, molecular docking and MD simulations were performed to investigate the basis for the potency of these thioglycosides for hOGA. This work sheds light on the structure-activity relationship of these thioglycosyl–naphthalimides against hOGA, and provides useful information for the further design and development of hOGA-related drugs.

## Materials and Methods

### Chemistry

All reagents and solvents were purchased from commercial sources and were used without further purification. The progress of reactions was routinely checked by thinlayer chromatography (TLC) on silica gel GF254 plates with detection by charring with 15% (v/v) H_2_SO_4_ in MeOH or by UV light (254 nm). ^1^H NMR and ^13^C NMR spectra were recorded on a Bruker AVANCE600 spectrometer with TMS as an internal reference and chemical shifts were reported in ppm (δ). High-resolution mass spectra (HRMS) was collected on a micro TOF-Q mass spectrometer of Bruker.

The specific synthetic procedures and characterization data for all the synthesized compounds can be found in Supporting Information.

### Biology

#### Enzyme Preparation

Human O-GlcNAcase (hOGA) was expressed in *Escherichia coli* BL21(DE3) and purified according to previous methods (Kong et al., 2016). Human β-N-acetylhexosaminidase B (HsHexB) was overexpressed in *Pichia pastoris* and then purified as described previously (Shen et al., 2018a).

#### Enzyme Inhibitory Activity Test

All target compounds were evaluated for their inhibitory activities against hOGA and HsHexB in end-point experiments. 4-Methylumbelliferyl N-acetyl-β-D-glucosaminide (4-MU-GlcNAc) was used as substrate. In a final assay volume of 100 μL, inhibitor and substrate (40 μM) were mixed with Britton-Robinson buffer (hOGA, pH 6.5; HsHexB, pH 4.5) and enzyme; then, the assay components were incubated at 30°C for 30 min. Subsequently, the enzymatic reaction was terminated by the addition of 100 μL 0.5 M Na_2_CO_3_ solution. The fluorescence of the liberated 4-methylumbelliferone (Mu) was quantified at an excitation of 366 nm and emission of 445 nm on a Varioskan Flash microplate reader (Thermo Fisher Scientific, USA). To determine the inhibition constant (*K*_i_), Dixon plots were performed by changing the concentration of the 4-MU-GlcNAc (40, 20, and 10 μM).

### Computational Methods

#### Molecular Docking

In this study, Sybyl 7.3 Software (Tripos Associates, 2006) was used for molecular docking. The crystal structure of the hOGA-PugNAc type inhibitor (PDB ID: 5M7T) (Roth et al., 2017) was obtained from the Protein Data Bank (http://www.rcsb.org/pdb) and selected as the starting model for docking. Before docking calculations, all water molecules were removed and missing hydrogen atoms were added to the protein. The MMFF94 force field was used to optimize the inhibitors to gain low energy conformations. Subsequently, the ligand mode was applied to generate the appropriate putative ligand pose (protomol), which was used for molecule docking (Jain, 1996; Welch et al., 1996). Finally, the protein–ligand complexes were achieved using the Surflex–Dock algorithm (ring flexibility was considered and default settings were chosen for the remaining parameters).

#### Molecular Dynamics (MD) Simulations

MD simulations of three systems (hOGA in complex with **10a**, **10d**, and **16j**) were performed after molecular docking in the Amber14 package (Case et al., 2014). For each system, AMBER03 force field (Duan et al., 2003) and GAFF force field (Wang et al., 2004) were selected for the protein and the ligand, respectively. This complex system was immersed in a truncated octahedral box with TIP3P water molecules, and an appropriate number of counterions (Cl^−^ or Na^+^) were added to achieve electrostatic neutrality. Subsequently, two stages of energy minimizations were conducted using the Sander module. The water molecules and the ligand were minimized by restraining the protein; then 2,500 cycles of the steepest-descent method and 2,500 cycles of the conjugated gradient algorithm were used to minimize all atoms of the systems. After that, the system was gradually heated from 0 to 300 K in the NVT ensemble and equilibrated to 300 K. Finally, full MD simulations were performed for 25 ns at a temperature of 300 K and a pressure of 10^5^ Pa, employing the PMEMD module in Amber14. The long-range electrostatic interactions under periodic boundary conditions were accounted for using the particle mesh Ewald (PME) method (Darden et al., 1993).

## Data Availability

All datasets analyzed for this study are included in the manuscript and the Supplementary Files.

## Author Contributions

JZ and QY designed and guided this investigation. SS and LD performed this study and wrote this paper. WC, RW, and HL implemented the modification of this paper in order to improve its quality. All authors read and approved the final manuscript.

### Conflict of Interest Statement

The authors declare that the research was conducted in the absence of any commercial or financial relationships that could be construed as a potential conflict of interest.

## References

[B1] Aoyagi T. Suda H. Uotani K. Kojima F. Aoyama T. Horiguchi K.. (1992). Nagstatin, a new inhibitor of N-acetyl-β-D-glucosaminidase, produced by Streptomyces amakusaensis MG846-fF3. Taxonomy, production, isolation, physico-chemical properties and biological activities. J. Antibiot. 45, 1404–1408. 10.7164/antibiotics.45.1404 1429224

[B2] Azad S. Kumamoto K. Uegaki K. Ichikawa Y. Kotsuki H. (2006). A new practical method for the synthesis of unsymmetrical ureas via high-pressure-promoted condensation of 2,2,2-trichloroethyl carbamates (Troc-carbamates) with amines. Tetrahedron Lett. 47, 587–590. 10.1016/j.tetlet.2005.11.045

[B3] Bergeron-Brlek M. Goodwin-Tindall J. Cekic N. Zandberg W. F. Shan X. Varghese V.. (2015). A convenient approach to stereoisomeric iminocyclitols: generation of potent brain-permeable OGA inhibitors. Angew. Chem. Int. Ed. 54, 15429–15433. 10.1002/anie.201507985 26545827

[B4] Cantarel B. L. Coutinho P. M. Rancurel C. Bernard T. Lombard V. Henrissat B. (2009). The Carbohydrate-Active EnZymes database (CAZy): an expert resource for glycogenomics. Nucleic Acids Res. 37, D233–D238. 10.1093/nar/gkn663 18838391 PMC2686590

[B5] Case D. A. Babin V. Berryman J. T. Betz R. M. Cai Q. Cerutti D. S. (2014). AMBER 14. San Francisco, CA: University of California.

[B6] Chen W. Shen S. Dong L. Zhang J. Yang Q. (2018). Selective inhibition of β-N-acetylhexosaminidases by thioglycosyl-naphthalimide hybrid molecules. Bioorg. Med. Chem. 26, 394–400. 10.1016/j.bmc.2017.11.042 29242020

[B7] Darden T. York D. Pedersen L. (1993). Particle mesh Ewald: an N·log(N) method for Ewald sums in large systems. J. Chem. Phys. 98, 10089–10092. 10.1063/1.464397

[B8] Dorfmueller H. C. Borodkin V. S. Schimpl M. van Aalten D. M. F. (2009). GlcNAcstatins are nanomolar inhibitors of human O-GlcNAcase inducing cellular hyper-O-GlcNAcylation. Biochem. J. 420, 221–227. 10.1042/BJ20090110 19275764 PMC2691177

[B9] Duan Y. Wu C. Chowdhury S. Lee M. C. Xiong G. Zhang W.. (2003). A point-charge force field for molecular mechanics simulations of proteins based on condensed-phase quantum mechanical calculations. J. Comput. Chem. 24, 1999–2012. 10.1002/jcc.10349 14531054

[B10] Elsen N. L. Patel S. B. Ford R. E. Hall D. L. Hess F. Kandula H.. (2017). Insights into activity and inhibition from the crystal structure of human O-GlcNAcase. Nat. Chem. Biol. 13, 613–615. 10.1038/nchembio.2357 28346407

[B11] Ferrer C. M. Lynch T. P. Sodi V. L. Falcone J. N. Schwab L. P. Peacock D. L.. (2014). O-GlcNAcylation regulates cancer metabolism and survival stress signaling via regulation of the HIF-1 pathway. Mol. Cell 54, 820–831. 10.1016/j.molcel.2014.04.026 24857547 PMC4104413

[B12] Henrissat B. Davies G. (1997). Structural and sequence-based classification of glycoside hydrolases. Curr. Opin. Struct. Biol. 7, 637–644. 10.1016/S0959-440X(97)80072-3 9345621

[B13] Igual M. O. Nunes P. S. G. da Costa R. M. Mantoani S. P. Tostes R. C. Carvalho I. (2019). Novel glucopyranoside C2-derived 1,2,3-triazoles displaying selective inhibition of O-GlcNAcase (OGA). Carbohydr. Res. 471, 43–55. 10.1016/j.carres.2018.10.007 30412832

[B14] Intra J. Pavesi G. Horner D. S. (2008). Phylogenetic analyses suggest multiple changes of substrate specificity within the Glycosyl hydrolase 20 family. BMC Evol. Biol. 8, 214–230. 10.1186/1471-2148-8-214 18647384 PMC2492878

[B15] Jain A. N. (1996). Scoring noncovalent protein-ligand interactions: a continuous differentiable function tuned to compute binding affinities. J. Comput. Aided. Mol. Des. 10, 427–440. 10.1007/BF00124474 8951652

[B16] Jang H. S. Kim H.-K. (2017). Novel direct synthesis of asymmetrical urea compounds from trichloroethyl carbamates using catalytic DBU. Bull. Korean Chem. Soc. 38, 1515–1518. 10.1002/bkcs.11314

[B17] Jeong B.-H. Kim H.-K. Thompson D. H. (2016). A facile and efficient method for the formation of unsymmetrical ureas using DABAL-Me-3. Aust. J. Chem. 69, 805–810. 10.1071/ch15675

[B18] Knapp S. Vocadlo D. Gao Z. Kirk B. Lou J. Withers S. G. (1996). NAG-thiazoline, an N-acetyl-β-hexosaminidase inhibitor that implicates acetamido participation. J. Am. Chem. Soc. 118, 6804–6805. 10.1021/JA960826U

[B19] Kong H. Lu H. Dong Y. Liang X. Jin S. Chen W.. (2016). Synthesis of NAM-thiazoline derivatives as novel O-GlcNAcase inhibitors. Carbohydr. Res. 429, 54–61. 10.1016/j.carres.2016.04.008 27233493

[B20] Kono M. Matsumoto T. Kawamura T. Nishimura A. Kiyota Y. Oki H.. (2013). Synthesis, SAR study, and biological evaluation of a series of piperazine ureas as fatty acid amide hydrolase (FAAH) inhibitors. Bioorg. Med. Chem. 21, 28–41. 10.1016/j.bmc.2012.11.006 23218778

[B21] Krejzova J. Simon P. Kalachova L. Kulik N. Bojarova P. Marhol P.. (2014). Inhibition of GlcNAc-processing glycosidases by C-6-azido-NAG-thiazoline and its derivatives. Molecules 19, 3471–3488. 10.3390/molecules19033471 24658571 PMC6271965

[B22] Liu T. Yan J. Yang Q. (2012). Comparative biochemistry of GH3, GH20 and GH84 β-N-acetyl-D-hexosaminidases and recent progress in selective inhibitor discovery. Curr. Drug Targets 13, 512–525. 10.2174/138945012799499730 22280348

[B23] Macauley M. S. Whitworth G. E. Debowski A. W. Chin D. Vocadlo D. J. (2005). O-GlcNAcase uses substrate-assisted catalysis: kinetic analysis and development of highly selective mechanism-inspired inhibitors. J. Biol. Chem. 280, 25313–25322. 10.1074/jbc.M413819200 15795231

[B24] Mahuran D. J. (1999). Biochemical consequences of mutations causing the GM2 gangliosidoses. Biochim. Biophys. Acta. 1455, 105–138. 10.1016/S0925-4439(99)00074-5 10571007

[B25] McClain D. A. Lubas W. A. Cooksey R. C. Hazel M. Parker G. J. Love D. C.. (2002). Altered glycan-dependent signaling induces insulin resistance and hyperleptinemia. Proc. Natl. Aacd. Sci. U.S.A. 99, 10695–10699. 10.1073/pnas.152346899 12136128 PMC125016

[B26] Paul B. Korytnyk W. (1984). S-, N-, and O-glycosyl derivatives of 2-acetamido-2-deoxy-D-glucose with hydrophobic aglycons as potential chemotherapeutic agents and N-acetyl-β-D-glucosaminidase inhibitors. Carbohydr. Res. 126, 27–43. 10.1016/0008-6215(84)85124-1 6713431

[B27] Roth C. Chan S. Offen W. A. Hemsworth G. R. Willems L. I. King D. T.. (2017). Structural and functional insight into human O-GlcNAcase. Nat. Chem. Biol. 13, 610–612. 10.1038/nchembio.2358 28346405 PMC5438047

[B28] Shen S. Chen W. Dong L. Yang Q. Lu H. Zhang J. (2018a). Design and synthesis of naphthalimide group-bearing thioglycosides as novel β-N-acetylhexosaminidases inhibitors. J. Enzyme Inhib. Med. Chem. 33, 445–452. 10.1080/14756366.2017.1419217 29390898 PMC6009855

[B29] Shen S. Dong L. Chen W. Lu H. Yang Q. Zhang J.. (2018b). Modification of the thioglycosyl-naphthalimides as potent and selective human O-GlcNAcase inhibitors. Acs Med. Chem. Lett. 9, 1241–1246. 10.1021/acsmedchemlett.8b00406 30613333 PMC6295843

[B30] Simard J. R. Getlik M. Grutter C. Pawar V. Wulfert S. Rabiller M.. (2009). Development of a fluorescent-tagged sinase assay system for the detection and characterization of allosteric kinase inhibitors. J. Am. Chem. Soc. 131, 13286–13296. 10.1021/ja902010p 19572644

[B31] Terinek M. Vasella A. (2005). Synthesis of N-acetylglucosamine-derived nagstatin analogues and their evaluation as glycosidase inhibitors. Helv. Chim. Acta 88, 10–22. 10.1002/hlca.200490286

[B32] Toleman C. Paterson A. J. Shin R. Kudlow J. E. (2006). Streptozotocin inhibits O-GlcNAcase via the production of a transition state analog. Biochem. Biophys. Res. Commun. 340, 526–534. 10.1016/j.bbrc.2005.12.041 16376298

[B33] Tripos Associates (2006). Sybyl 7.3. St. Louis, MO: Tripos Associates.

[B34] Wang J. Wolf R. M. Caldwell J. W. Kollman P. A. Case D. A. (2004). Development and testing of a general amber force field. J. Comput. Chem. 26:114 10.1002/jcc.20145 15116359

[B35] Welch W. Ruppert J. Jain A. N. (1996). Hammerhead: fast, fully automated docking of flexible ligands to protein binding sites. Chem. Biol. 3, 449–462. 10.1016/S1074-5521(96)90093-9 8807875

[B36] Yuzwa S. A. Macauley M. S. Heinonen J. E. Shan X. Dennis R. J. He Y.. (2008). A potent mechanism-inspired O-GlcNAcase inhibitor that blocks phosphorylation of tau *in vivo*. Nat. Chem. Biol. 4, 483–490. 10.1038/nchembio.96 18587388

[B37] Yuzwa S. A. Vocadlo D. J. (2014). O-GlcNAc and neurodegeneration: biochemical mechanisms and potential roles in Alzheimer's disease and beyond. Chem. Soc. Rev. 43, 6839–6858. 10.1039/C4CS00038B 24759912

[B38] Zhang T. Hu X. Dong X. Li G. Lu H. (2018). Iridium-catalyzed unreactive C(sp(3))-H amination with 2,2,2-trichloroethoxycarbonyl azide. Org. Lett. 20, 6260–6264. 10.1021/acs.orglett.8b02738 30232895

