# Supplementary material for: Design and Optimization of Thioglycosyl–naphthalimides as Efficient Inhibitors Against Human O-GlcNAcase
Source: Front Chem. 2019 Jul 25;7:533. doi: 10.3389/fchem.2019.00533 (PMC6669961; doi:10.3389/fchem.2019.00533)

**Design and Optimization of thioglycosyl−naphthalimides as efficient inhibitors against Human O‑GlcNAcase**

Shengqiang Shen,^†,#^ Lili Dong,^†,#^ Wei Chen,^‡^ Renjie Wu,^†^ Huizhe Lu,^†^ Qing Yang,*^,‡^ and

Jianjun Zhang*^,†^

^†^Department of Applied Chemistry, College of Science, China Agricultural University, Beijing, China

^‡^Institute of Plant Protection, Chinese Academy of Agricultural Sciences, Beijing 100193, China

**Contents**

[1. Synthetic procedures and characterization of compounds 3](#_Toc1541)

[1.1. Synthesis of thioglycosyl−naphthalimides 10a-10d 3](#_Toc10345)

[1.2. Synthesis of thioglycosyl−naphthalimides 16a-16o. 8](#_Toc22019)

[1.3. New method for the synthesis of thioglycosyl−naphthalimides 16h. 21](#_Toc31375)

[1.4. Synthesis of ureido glycosides 17a-17d. 22](#_Toc460)

[2. The supplementary MD simulations studies. 23](#_Toc22179)

[References 25](#_Toc31972)

[^1^H NMR and ^13^C NMR spectrum 26](#_Toc11907)

1. **Synthetic procedures and characterization of compounds**

**1.1. Synthesis of thioglycosyl−naphthalimides 10a-10d**

**10a**: R_1_=Br; **10b**: R_1_=OCH_3_; **10c**: R_1_=N(CH_3_)_2_; **10d**: R_1_=piperidyl

**Scheme S1.** Synthesis of thioglycosyl−naphthalimides **10a-10d**. (i) *tert*-butyl (3-aminopropyl) carbamate, EtOH; (ii) DCM, CF_3_COOH; (iii) 2-chloroacetyl chloride, Et_3_N, DCM; (iv) AcCl; (v) thiourea, acetone; (vi) Na_2_S_2_O_5_, DCM, H_2_O; (vii) K_2_CO_3_, acetone, H_2_O; (viii) CH_3_NH_2_, MeOH.

Compounds **5a-5d** were synthesized as described previously.^1^

2-(3-aminopropyl)-6-bromo-1*H*-benzo[*de*]isoquinoline-1,3(2*H*)-dione (**5a**): light yellow solid; (2.6 g, 79.1%) yield; ^1^H NMR (300 MHz, DMSO-*d*_6_) δ 8.52 –8.48 (m, 2H, ArH), 8.23 (d, *J* = 8.3 Hz, 1H, ArH), 8.16 (dd, *J* = 8.1, 7.6 Hz, 1H, ArH), 7.99 (d, *J* = 8.5 Hz, 1H, ArH), 4.07 (t, *J* = 6.8 Hz, 2H, CH_2_), 2.61 (t, *J* = 6.8 Hz, 2H, CH_2_), 2.03–1.93 (m, 2H, CH_2_).

2-(3-aminopropyl)-6-methoxy-1H-benzo[de]isoquinoline-1,3(2H)-dione (**5b**): light yellow solid; 1H NMR (300 MHz, DMSO-d6) δ 8.41 (dd, *J* = 7.8, 3.2 Hz, 2H, ArH), 8.35 (d, *J* = 8.3 Hz, 1H, ArH), 7.74 (dd, *J* = 8.1, 7.6 Hz, 1H, ArH), 7.24 (d, *J* = 8.5 Hz, 1H, ArH), 4.19 – 4.00 (m, 5H, OCH_3_, CH_2_), 2.82 (dd, *J* = 9.0, 6.8 Hz, 2H, CH_2_), 2.04 – 1.94 (m, 2H, CH_2_).

2-(3-aminopropyl)-6-(dimethylamino)-1*H*-benzo[*de*]isoquinoline-1,3(2*H*)-dione (**5c**): yellow solid; ^1^H NMR (300 MHz, CDCl_3_) δ 8.57 (d, J = 8.2 Hz, 1H, ArH), 8.49 – 8.41 (m, 2H, ArH), 7.70 (dd, *J* = 8.2, 7.5 Hz, 1H, ArH), 7.13 (d, J = 8.2 Hz, 1H, ArH), 4.30 (t, *J* = 6.9 Hz, 2H, CH_2_), 2.79 – 2.73 (m, 2H, CH_2_), 2.34 (s, 6H, 2 CH_3_), 1.99 – 1.85 (m, 2H, CH_2_).

2-(3-aminopropyl)-6-(piperidin-1-yl)-1*H*-benzo[*de*]isoquinoline-1,3(2*H*)-dione (**5d**): yellow solid; ^1^H NMR (300 MHz, CDCl_3_) δ 8.55 (dd, *J* = 7.3, 1.1 Hz, 1H, ArH), 8.46 (d, *J* = 8.1 Hz, 1H, ArH), 8.37 (dd, *J* = 8.4, 1.1 Hz, 1H, ArH), 7.65 (dd, *J* = 8.4, 7.4 Hz, 1H, ArH), 7.16 (d, *J* = 8.2 Hz, 1H, ArH), 4.31 (t, *J* = 5.6 Hz, 2H, CH_2_), 3.27 – 3.14 (m, 4H, 2 CH_2_), 2.82 – 2.73 (m, 2H, CH_2_), 2.08 – 1.81 (m, 6H, 3 CH_2_), 1.79 – 1.61 (m, 2H, CH_2_).

A solution of **5a-5d** (5 mmol, 1 eq) in dry DCM (50 mL) was mixed with Et_3_N (1.5 mL,7.5 mmol) at 0 ℃, then 2-chloroacetyl chloride (0.6 mL, 6 mmol) in dry DCM (10 mL) was added dropwise under N_2_. The mixture was further stirred for 3 h at room temperature, and the completion of the reaction was confirmed by TLC (petroleum ether/EtOAc, 3:1 v/v) analysis. After the reaction mixture was concentrated *in vacuo*, **6a-6d** were prepared by flash column chromatography (petroleum ether/EtOAc, 6:1 v/v).

*N*-(3-(6-bromo-1,3-dioxo-1*H*-benzo[*de*]isoquinolin-2(3*H*)-yl)propyl)-2-chloroacetamide (**6a**): light yellow solid; (1.8 g, 87.9 %) yield; ^1^H NMR (300 MHz, CDCl_3_) δ 8.67 (d, *J* = 7.3 Hz, 1H, ArH), 8.58 (dd, *J* = 8.5, 0.8 Hz, 1H, ArH), 8.42 (d, *J* = 7.9 Hz, 1H, ArH), 8.05 (d, *J* = 7.9 Hz, 1H, ArH), 7.86 (dd, *J* = 8.4, 7.5 Hz, 1H, ArH), 7.50 (br s, 1H, NH), 4.27 (t, *J* = 6.3 Hz, 2H, ArCH_2_), 4.10 (s, 2H, ClCH_2_), 3.34 (dd, *J* = 12.3, 6.2 Hz, 2H, CH_2_), 2.10 – 1.93 (m, 2H, CH_2_); ^13^C NMR (75 MHz, CDCl_3_) δ 165.13, 163.03, 132.59, 131.34, 130.50, 130.18, 129.66, 127.99, 127.14, 121.74, 120.85, 41.76, 36.56, 35.57, 26.86.

2-chloro-*N*-(3-(6-methoxy-1,3-dioxo-1*H*-benzo[*de*]isoquinolin-2(3*H*)-yl)propyl)acetamide (**6b**): light yellow solid; (1.5 g, 83.1 %) yield; ^1^H NMR (300 MHz, DMSO-*d*_6_) δ 8.59 – 8.38 (m, 3H, ArH), 8.24 (t, J = 5.2 Hz, 1H, NH), 7.85 – 7.74 (m, 1H, ArH), 7.30 (d, *J* = 8.4 Hz, 1H, ArH), 4.11 (s, 3H, OCH_3_), 4.09 – 3.95 (m, 4H, ArCH_2_, ClCH_2_), 3.21 – 3.06 (m, 2H, CH_2_), 1.84 – 1.70 (m, 2H, CH_2_); ^13^C NMR (75 MHz, DMSO-*d*_6_) δ 163.71, 162.87, 160.32, 132.88, 130.91, 128.52, 127.90, 125.94, 122.76, 121.97, 114.32, 106.21, 56.53, 41.80, 37.15, 35.94, 27.27.

2-chloro-*N*-(3-(6-(dimethylamino)-1,3-dioxo-1*H*-benzo[*de*]isoquinolin-2(3*H*)-yl)propyl)acetamide (**6c**): yellow solid; (1.6 g, 85.6 %) yield; ^1^H NMR (300 MHz, CDCl_3_) δ 8.67 – 8.41 (m, 3H, ArH), 7.72 (d, *J* = 8.2 Hz, 1H, ArH), 7.57 (s, 1H, NH), 7.13 (d, J = 8.2 Hz, 1H, ArH), 4.29 (t, *J* = 6.7 Hz, 2H, ArCH_2_), 4.11 (s, 2H, ClCH_2_), 3.39 – 3.28 (m, 2H, CH_2_), 2.33 (s, 6H, 2 CH_3_), 1.98 – 1.80 (m, 2H, CH_2_); ^13^C NMR (75 MHz, DMSO-*d*_6_) δ 163.51, 163.04, 156.52, 132.05, 131.29, 130.37, 129.40, 124.91, 124.15, 122.18, 113.22, 112.70, 44.31, 42.01, 37.13, 35.88, 27.07.

2-chloro-*N*-(3-(1,3-dioxo-6-(piperidin-1-yl)-1*H*-benzo[*de*]isoquinolin-2(3*H*)-yl)propyl)acetamide (**6d**): yellow solid; (1.7 g, 82.1 %) yield; ^1^H NMR (300 MHz, DMSO-*d*_6_) δ 8.70 – 8.47 (m, 2H, ArH), 8.40 (d, *J* = 8.5, 1H, NH), 7.78 – 7.62 (m, 2H, ArH, NH), 7.18 (d, *J* = 8.2 Hz, 1H, ArH), 4.18 – 4.05 (m, 4H, ArCH_2_, ClCH_2_), 3.38 – 3.29 (m, 2H, CH_2_), 3.28 – 3.19 (m, 4H, 2 CH_2_), 2.02 – 1.94 (m, 2H, CH_2_), 1.93 – 1.84 (m, 4H, 2 CH_2_), 1.79 – 1.69 (m, 2H, CH_2_); ^13^C NMR (75 MHz, DMSO-*d*_6_) δ 163.81, 163.21, 156.71, 132.36, 130.67, 129.34, 125.90, 125.55, 122.70, 120.82, 115.13, 115.01, 54.09, 41.79, 36.90, 35.64, 27.14, 25.83, 23.96.

2-acetamido-3,4,6-tri-O-acetyl-2-deoxy-1-thio-β-D-glucopyranose (**8**) was synthesized from N-acetyl-D-glucosamine as described previously.^2^ White solid; (65.2 % over three steps) yield; [α]_D_^25^ -15.1 (c=1.0,CHCl_3_); ^1^H NMR (300 MHz, CDCl_3_) δ 5.78 (d, *J* = 9.5 Hz, 1H, NH), 5.20 – 5.03 (m, 2H, H-3, H-4), 4.60 (d, *J* = 9.9 Hz, 1H, H-1), 4.25 (dd, *J* = 12.4, 4.8 Hz, 1H, H-6b), 4.19 – 4.05 (m, 2H, H-6a, H-2), 3.70 (ddd, *J* = 9.8, 4.7, 2.2 Hz, 1H, H-5), 2.57 (s, 1H, SH), 2.10 (s, 3H, OAc), 2.05 (s, 3H, OAc), 2.03 (s, 3H, OAc), 1.99 (s, 3H, NAc).

A solution of **6a-6d** (2 mmol, 1.0 eq) in acetone (60 mL) and H_2_O (30 mL) was mixed with solid potassium carbonate (2.4 mmol, 1.2 eq), then **8** (2 mmol, 1 eq) was added. The mixture was stirred for 5 h at room temperature until TLC (petroleum ether/EtOAc, 1:2 v/v) indicated that the reaction was complete. After the solution was concentrated *in vacuo*, the residue was diluted with DCM (80 mL), washed with H_2_O (100 mL), brine (100 mL), dried over Na_2_SO_4_, and concentrated. Finally, the residue was purified by flash column chromatography (petroleum ether/EtOAc, 2:1 v/v) to obtain **9a-9d**.

*N*-[3-(6-bromo-1,3-dioxo-1*H*-benzo[*de*]isoquinolin-2(3*H*)-yl)propyl]-2-[(2-acetamido-3,4,6- tri-O-acetyl-β-D-glucopyranosyl)thio]acetamide (**9a**): light yellow solid; (1.2 g, 81.6 %) yield; [α]_D_^25^ -100.5 (c=1, DMSO); ^1^H NMR (300 MHz, DMSO-*d*_6_) δ 8.51 (ddd, *J* = 9.4, 7.9, 0.9 Hz, 2H, ArH), 8.29 (d, *J* = 7.9 Hz, 1H, ArH), 8.17 (d, *J* = 7.9 Hz, 1H, ArH), 8.07 – 7.91 (m, 3H, 2 NH, ArH), 5.11 (t, *J* = 9.7 Hz, 1H, H-3), 4.90 (t, *J* = 9.7 Hz, 1H, H-4), 4.80 (d, *J* = 10.4 Hz, 1H, H-1), 4.18 (dd, *J* = 12.3, 4.8 Hz, 1H, H-6b), 4.09 – 3.99 (m, 3H, CH_2_Ar, H-6a), 3.99 – 3.91 (m, 1H, H-2), 3.90 – 3.80 (m, 1H, H-5), 3.34 – 3.21 (m, 2H, SCH_2_), 3.16 (dd, *J* = 13.1, 6.4 Hz, 2H, CH_2_), 1.99, 1.97 (2 s, 6H, 2 OAc), 1.93 (s, 3H, OAc), 1.86 – 1.72 (m, 5H, NAc, CH_2_); ^13^C NMR (75 MHz, DMSO-*d*_6_) δ 170.13, 169.73, 169.37, 168.37, 163.05, 163.00, 132.69, 131.67, 131.42, 131.05, 129.87, 129.22, 128.85, 128.39, 122.86, 122.08, 83.00, 74.80, 73.68, 68.67, 62.08, 52.17, 38.05, 37.01, 32.88, 27.87, 22.73, 20.56, 20.52, 20.45; HRMS (ESI) calcd for C_31_H_35_BrN_3_O_11_S (M+H^+^) 736.1176, found 736.1169.

*N*-[3-(6-methoxy-1,3-dioxo-1*H*-benzo[*de*]isoquinolin-2(3*H*)-yl)propyl]-2-[(2-acetamido-3,4,6- tri-O-acetyl-β-D-glucopyranosyl)thio]acetamide (**9b**): light yellow solid; (1.2 g, 87.5 %) yield; [α]_D_^25^ -91.3 (c=1, DMSO); ^1^H NMR (300 MHz, DMSO-*d*_6_) δ 8.47 – 8.29 (m, 3H, ArH), 8.07 – 7.89 (m, 2H, 2 NH), 7.76 – 7.68 (m, 1H, ArH), 7.22 (d, *J* = 8.5 Hz, 1H, ArH), 5.09 (t, *J* = 9.7 Hz, 1H, H-3), 4.87 (t, *J* = 9.7 Hz, 1H, H-4), 4.77 (d, *J* = 10.4 Hz, 1H, H-1), 4.15 (dd, *J* = 12.3, 4.7 Hz, 1H, H-6b), 4.08 (s, 3H, OCH_3_), 4.03 – 3.95 (m, 3H, CH_2_Ar, H-6a), 3.94 – 3.88 (m, 1H, H-2), 3.85 – 3.78 (m, 1H, H-5), 3.32 – 3.17 (m, 2H, SCH_2_), 3.16 – 3.03 (m, 2H, CH_2_), 1.96 (s, 3H, OAc), 1.94 (s, 3H, OAc), 1.90 (s, 3H, OAc), 1.79 – 1.69 (m, 5H, NAc, CH_2_); ^13^C NMR (75 MHz, DMSO-*d*_6_) δ 170.14, 169.73, 169.40, 169.38, 168.36, 163.72, 163.07, 160.42, 133.34, 131.09, 128.59, 128.30, 126.37, 122.78, 121.90, 114.26, 106.27, 83.00, 74.82, 73.70, 68.68, 62.06, 56.71, 52.20, 37.70, 37.03, 32.88, 28.03, 22.72, 20.54, 20.50, 20.44; HRMS (ESI) calcd for C_32_H_38_N_3_O_12_S (M+H^+^) 688.2176, found 688.2162.

*N*-[3-(6-dimethylamino-1,3-dioxo-1*H*-benzo[*de*]isoquinolin-2(3*H*)-yl)propyl]-2-[(2-acetamido-3,4,6- tri-O-acetyl-β-D-glucopyranosyl)thio]acetamide (**9c**): yellow solid; (1.3 g, 92.8 %) yield; [α]_D_^25^ -103.8 (c=1, DMSO); ^1^H NMR (300 MHz, DMSO-*d*_6_) δ 8.46 – 8.33 (m, 2H, ArH), 8.24 (d, *J* = 8.3 Hz, 1H, ArH), 8.07 – 7.87 (m, 2H, 2 NH), 7.66 (dd, *J* = 8.3, 7.5 Hz, 1H, ArH), 7.10 (d, *J* = 8.4 Hz, 1H, ArH), 5.10 (t, *J* = 9.7 Hz, 1H, H-3), 4.87 (t, *J* = 9.7 Hz, 1H, H-4), 4.78 (d, *J* = 10.4 Hz, 1H, H-1), 4.16 (dd, *J* = 12.3, 4.8 Hz, 1H, H-6b), 4.04 – 3.96 (m, 3H, CH_2_Ar, H-6a), 3.94 – 3.87 (m, 1H, H-2), 3.85 – 3.76 (m, 1H, H-5), 3.34 – 3.18 (m, 2H, SCH_2_), 3.11 (dd, *J* = 13.2, 6.6 Hz, 2H, CH_2_), 3.04 (s, 6H, 2 NCH_3_), 1.96 (s, 3H, OAc), 1.94 (s, 3H, OAc), 1.90 (s, 3H, OAc), 1.79 – 1.69 (m, 5H, CH_2_, NAc); ^13^C NMR (75 MHz, DMSO-*d*_6_) δ 170.13, 169.74, 169.41, 169.38, 168.35, 163.77, 163.11, 156.62, 132.36, 131.56, 130.62, 129.66, 124.96, 124.23, 122.31, 113.30, 112.96, 83.00, 74.83, 73.71, 68.67, 62.05, 52.21, 44.45, 37.56, 37.01, 32.88, 28.06, 22.71, 20.53, 20.49, 20.43; HRMS (ESI) calcd for C_33_H_41_N_4_O_11_S (M+H^+^) 701.2493, found 701.2497.

*N*-[3-[6-(piperidin-1-yl)-1,3-dioxo-1*H*-benzo[*de*]isoquinolin-2(3*H*)-yl]propyl]-2-[(2-acetamido-3,4,6- tri-O-acetyl-β-D-glucopyranosyl)thio]acetamide (**9d**): yellow solid; (1.2 g, 81.1 %) yield; [α]_D_^25^ -107.1 (c=1, DMSO); ^1^H NMR (300 MHz, DMSO-*d*_6_) δ 8.53 – 8.44 (m, 1H, ArH), 8.43 – 8.34 (m, 2H, ArH), 8.07 – 7.91 (m, 2H, 2 NH), 7.80 (dd, *J* = 8.4, 7.4 Hz, 1H, ArH), 7.30 (d, *J* = 8.2 Hz, 1H, ArH), 5.10 (t, *J* = 9.7 Hz, 1H, H-3), 4.88 (t, *J* = 9.7 Hz, 1H, H-4), 4.79 (d, *J* = 10.4 Hz, 1H, H-1), 4.17 (dd, *J* = 12.3, 4.8 Hz, 1H, H-6b), 4.09 – 3.98 (m, 3H, CH_2_Ar, H-6a), 3.97 – 3.87 (m, 1H, H-2), 3.87 – 3.78 (m, 1H, H-5), 3.33 – 3.23 (m, 2H, SCH_2_), 3.23 – 3.08 (m, 6H, 3 CH_2_), 1.98 (s, 3H, OAc), 1.97 (s, 3H, OAc), 1.92 (s, 3H, OAc), 1.89 – 1.77 (m, 6H, 3 CH_2_), 1.77 (s, 3H, NAc), 1.72 – 1.59 (m, 2H, CH_2_); ^13^C NMR (75 MHz, DMSO-*d*_6_) δ 170.13, 169.72, 169.37, 168.32, 163.80, 163.25, 156.89, 132.44, 130.75, 129.34, 125.93, 125.58, 122.65, 115.12, 115.03, 82.99, 74.81, 73.70, 68.67, 62.06, 54.10, 52.19, 37.65, 37.00, 32.87, 28.05, 25.84, 23.97, 22.73, 20.56, 20.52, 20.45; HRMS (ESI) calcd for C_36_H_45_N_4_O_11_S (M+H^+^) 741.2806, found 741.2818.

A solution of **9a-9d** (1.0 mmol) was suspended in anhydrous MeOH (15 mL), and saturated solution of CH_3_NH_2_ in MeOH (10 mL) was then added. The reaction was stirred for 48 h at room temperature, until TLC (EtOAc/MeOH/H_2_O, 8:1:1 v/v/v) indicated that the reaction was complete. The mixture was concentrated *in vacuo* and recrystallized from MeOH/ether to obtain **10a-10d**.

*N*-[3-(6-bromo-1,3-dioxo-1*H*-benzo[*de*]isoquinolin-2(3*H*)-yl)propyl]-2-[(2-acetamido-β-D-glucopyranosyl)thio]acetamide (**10a**): yellow solid; (0.51 g, 83.6 %) yield; [α]_D_^25^ -30.1 (c=0.2, DMSO); ^1^H NMR (300 MHz, DMSO-*d*_6_) δ 8.52 – 8.37 (m, 2H, ArH), 8.22 (d, *J* = 7.9 Hz, 1H, ArH), 8.11 (d, *J* = 7.9 Hz, 1H, ArH), 7.98 – 7.87 (m, 2H, ArH, NH), 7.79 (d, *J* = 9.3 Hz, 1H, NH), 4.49 (d, *J* = 10.3 Hz, 1H, H-1), 4.03 (t, *J* = 7.1 Hz, 2H, H-3, H-4), 3.72 (d, *J* = 11.0 Hz, 1H, H-6b), 3.58 (dd, *J* = 19.6, 9.8 Hz, 1H, H-2), 3.47 (dd, *J* = 11.6, 5.4 Hz, 1H, H-6a), 3.42-3.22 (m, 3H, H-5, SCH_2_), 3.21 – 3.12 (m, 4H, 2 CH_2_), 1.88 – 1.73 (m, 5H, NAc, CH_2_); ^13^C NMR (75 MHz, DMSO-*d*_6_) δ 169.32, 169.10, 162.94, 162.88, 132.59, 131.59, 131.34, 130.96, 129.74, 129.19, 128.77, 128.22, 122.70, 121.92, 83.83, 81.25, 75.45, 70.70, 61.44, 54.59, 38.06, 37.08, 33.14, 27.91, 23.13; HRMS (ESI) calcd for C_25_H_29_BrN_3_O_8_S (M+H^+^) 610.0859, found 610.0842.

*N*-[3-(6-methoxy-1,3-dioxo-1*H*-benzo[*de*]isoquinolin-2(3*H*)-yl)propyl]-2-[(2-acetamido-β-D-glucopyranosyl)thio]acetamide (**10b**): yellow solid; (0.50 g, 89.3 %) yield; [α]_D_^25^ -47.3 (c=0.2, DMSO); ^1^H NMR (300 MHz, DMSO-*d*_6_) δ 8.38 – 8.32 (m, 2H, ArH), 8.29 (d, *J* = 8.3 Hz, 1H, ArH), 7.90 (t, *J* = 5.7 Hz, 1H, NH), 7.82 – 7.60 (m, 2H, ArH), 7.17 (d, *J* = 8.5 Hz, 1H, NH), 5.05 (d, *J* = 4.6 Hz, 1H, OH), 5.01 (d, *J* = 5.3 Hz, 1H, OH), 4.56 (t, *J* = 5.7 Hz, 1H, OH), 4.45 (d, *J* = 10.3 Hz, 1H, H-1), 4.06 (s, 3H, OCH_3_), 3.98 (t, *J* = 7.1 Hz, 2H, H-3, H-4), 3.69 (dd, *J* = 11.0, 6.1 Hz, 1H, H-6b), 3.54 (dd, *J* = 19.6, 9.7 Hz, 1H, H-2), 3.44 (dd, *J* = 11.4, 5.6 Hz, 1H, H-6a), 3.34 – 3.15 (m, 3H, H-5, SCH_2_), 3.15 – 3.04 (m, 4H, 2 CH_2_), 1.78 (s, 3H, NAc), 1.77 – 1.68 (m, 2H, CH_2_); ^13^C NMR (75 MHz, DMSO-*d*_6_) δ 169.35, 169.08, 163.64, 162.99, 160.34, 133.27, 131.01, 128.48, 128.23, 126.30, 122.69, 121.78, 114.15, 106.21, 83.79, 81.24, 75.46, 70.70, 61.44, 56.68, 54.60, 37.70, 37.10, 33.10, 28.08, 23.11; HRMS (ESI) calcd for C_26_H_32_N_3_O_9_S (M+H^+^) 562.1859, found 562.1849.

*N*-[3-(6-dimethylamino-1,3-dioxo-1*H*-benzo[*de*]isoquinolin-2(3*H*)-yl)propyl]-2-[(2-acetamido-β-D-glucopyranosyl)thio]acetamide (**10c**): yellow solid; (0.49 g, 87.5 %) yield; [α]_D_^25^ -39.8 (c=0.2, DMSO); ^1^H NMR (300 MHz, DMSO-*d*_6_) δ 8.49 – 8.31 (m, 2H, ArH), 8.23 (d, *J* = 8.3 Hz, 1H, ArH), 7.89 (t, *J* = 5.7 Hz, 1H, NH), 7.75 (d, *J* = 9.3 Hz, 1H, ArH), 7.66 (dd, *J* = 8.4, 7.4 Hz, 1H, ArH, NH), 7.10 (d, *J* = 8.4 Hz, 1H, ArH), 5.05 (d, *J* = 4.7 Hz, 1H, OH), 5.01 (d, *J* = 5.3 Hz, 1H, OH), 4.57 (t, *J* = 5.7 Hz, 1H, OH), 4.45 (d, *J* = 10.3 Hz, 1H, H-1), 3.98 (t, *J* = 7.1 Hz, 2H, H-3, H-4), 3.69 (dd, *J* = 11.0, 6.0 Hz, 1H, H-6b), 3.55 (dd, *J* = 19.6, 9.7 Hz, 1H, H-2), 3.45 (dd, *J* = 11.5, 5.8 Hz, 1H, H-6a), 3.35 – 3.15 (m, 3H, H-5, SCH_2_), 3.15 – 3.06 (m, 4H, 2 CH_2_), 3.04 (s, 6H, 2 NCH_3_), 1.78 (s, 3H, NAc), 1.76 – 1.69 (m, 2H, CH_2_); ^13^C NMR (75 MHz, DMSO-*d*_6_) δ 169.35, 169.05, 163.75, 163.09, 156.62, 132.35, 131.57, 130.61, 129.65, 124.98, 124.22, 122.29, 113.25, 112.97, 83.77, 81.24, 75.48, 70.70, 61.44, 54.60, 44.47, 37.59, 37.09, 33.10, 28.13, 23.11; HRMS (ESI) calcd for C_27_H_35_N_4_O_8_S (M+H^+^) 575.2176, found 575.2184.

*N*-[3-[6-(piperidin-1-yl)-1,3-dioxo-1*H*-benzo[*de*]isoquinolin-2(3*H*)-yl]propyl]-2-[(2-acetamido-β-D-glucopyranosyl)thio]acetamide (**10d**): yellow solid; (0.53 g, 86.9 %) yield; [α]_D_^25^ -45.4 (c=0.2, DMSO); ^1^H NMR (300 MHz, DMSO-*d*_6_) δ 8.49 – 8.39 (m, 1H, ArH), 8.38 – 8.28 (m, 2H, ArH), 7.94 (t, *J* = 5.7 Hz, 1H, H-1), 7.86 – 7.68 (m, 2H, ArH, NH), 7.24 (d, *J* = 8.2 Hz, 1H, ArH), 4.50 (d, *J* = 10.3 Hz, 1H, H-1), 4.28 (br s, 3H, 3 OH), 4.04 (t, *J* = 7.0 Hz, 2H, H-3, H-4), 3.74 (d, *J* = 11.3 Hz, 1H, H-6b), 3.59 (dd, *J* = 19.6, 9.8 Hz, 1H, H-2), 3.48 (dd, *J* = 11.5, 5.3 Hz, 1H, H-6a), 3.40 – 3.22 (m, 3H, H-5, SCH_2_), 3.20 – 3.07 (m, 8H, 4 CH_2_), 1.89 – 1.76 (m, 9H, NAc, 3 CH_2_), 1.72 – 1.59 (m, 2H, CH_2_); ^13^C NMR (75 MHz, DMSO-*d*_6_) δ 169.31, 169.04, 163.70, 163.15, 156.79, 132.34, 130.65, 129.22, 125.82, 125.48, 122.52, 115.01, 114.93, 83.81, 81.26, 75.46, 70.71, 61.44, 54.60, 54.05, 37.65, 37.07, 33.11, 28.11, 25.82, 23.96, 23.11; HRMS (ESI) calcd for C_30_H_39_N_4_O_8_S (M+H^+^) 615.2489, found 615.2474.

**1.2. Synthesis of thioglycosyl−naphthalimides 16a-16o.**

**14a**: R_2_=Ph; **14b**: R_2_=4-FPh; **14c**: R_2_=BnO; **14d**: R_2_=CH_2_CH_3_; **14e**: R_2_=CH_2_CH_2_CH_3_;

**14f**: R_2_=CH(CH_3_)_2_; **14g**: R_2_=NHCH_3_; **14h**: R_2_=OCH_2_CCl_3_; **14i**: R_2_=CF_3_

**15a**: R_1_=piperidyl, R_2_=Ph; **15b**: R_1_=piperidyl, R_2_=4-FPh; **15c**: R_1_=piperidyl, R_2_=BnO;

**15d**: R_1_=piperidyl, R_2_=C_2_H_5_; **15e**: R_1_=piperidyl, R_2_=n-Pr; **15f**: R_1_=piperidyl, R_2_=i-Pr;

**15g**: R_1_=piperidyl, R_2_=CF_3_; **15h**: R_1_=piperidyl, R_2_=NHCH_3_; **15i**: R_1_=piperidyl, R_2_=OCH_2_CCl_3_;

**15j**: R_1_=Br, R_2_=Ph; **15k**: R_1_=Br, R_2_=4-FPh; **15l**: R_1_=Br, R_2_=Et; **15m**: R_1_=Br, R_2_=n-Pr;

**15n**: R_1_=Br, R_2_=i-Pr; **15o**: R_1_=Br, R_2_=CF_3_; **15p**: R_1_=Br, R_2_=OCH_2_CCl_3_

**16a**: R_1_=piperidyl, R_2_=Ph; **16b**: R_1_=piperidyl, R_2_=4-FPh; **16c**: R_1_=piperidyl, R_2_=BnO;

**16d**: R_1_=piperidyl, R_2_=C_2_H_5_; **16e**: R_1_=piperidyl, R_2_=n-Pr; **16f**: R_1_=piperidyl, R_2_=i-Pr;

**16g**: R_1_=piperidyl, R_2_=CF_3_; **16h**: R_1_=piperidyl, R_2_=NHCH_3_; **16j**: R_1_=Br, R_2_=Ph;

**16k**: R_1_=Br, R_2_=4-FPh; **16l**: R_1_=Br, R_2_=Et; **16m**: R_1_=Br, R_2_=n-Pr; **16n**: R_1_=Br, R_2_=i-Pr;

**16o**: R_1_=Br, R_2_=CF_3_

**Scheme S2.** Synthesis of thioglycosyl−naphthalimides **16a-16o**. (i) p-anisaladehyde, NaOH, H_2_O; (ii) Py, Ac_2_O; (iii) acetone, HCl, H_2_O; (iv) Et_3_N, CH_2_Cl_2,_ R_2_COCl for **13a-13h**; Et_3_N, CH_2_Cl_2,_ TFAA for **13i**; (v) HBr, CH_3_COOH, CH_2_Cl_2_; (vi) thiourea, acetone; (vii) Na_2_S_2_O_5_, CH_2_Cl_2_, H_2_O; (viii) K_2_CO_3_, acetone, H_2_O, **6a** or **6d**; (viii) CH_3_NH_2_, MeOH.

Compound **12** was synthesized according to procedures described in the literature.^3^

Compounds **13a-13i** were prepared according to published methods.^4,5^

A solution of compound **13a-13i** (10 mmol) in anhydrous CH_2_Cl_2_ (30 mL) at 0°C, then 33% HBr/HOAc was added (8 mL) was added dropwise. The solution was stirred for 3 h at room temperature and the completion of the reaction was confirmed by TLC (petroleum ether/EtOAc, 1:1 v/v) analysis. The reaction was then quenched with chilled H_2_O (200 mL), and the mixture was extracted with CH_2_Cl_2_ (3×100 ml). The organic phase was washed with water (2×40 mL), sat. NaHCO_3_ (50 mL) and dried over Na_2_SO_4_. The resulting solution was concentrated to a white foam and carried on without further purification. The resulting white foam was dissolved in 80 mL acetone, and thiourea (0.91 g, 12 mmol) was then added. The mixture was heated to reflux for 4 h until a white precipitate was observed. The solid precipitate was then filtered and washed with acetone (2×10 mL). Subsequently, the precipitate was dissolved in CH_2_Cl_2_ (60 mL) and H_2_O (40 mL), then Na_2_S_2_O_5_ (2.1 g, 11.0 mmol) was added. The resulting mixture was stirred vigorously and heated to reflux for 5 h, until TLC (EtOAc) indicated that the reaction was complete. The mixture was extracted with CH_2_Cl_2_ (3×30 mL). The combined organic layers were dried over Na_2_SO_4_ and concentrated. Finally, the resulting compounds **14a-14i** were obtained by recrystallized from petroleum ether/EtOAc.

2-benzamido-3,4,6-tri-O-acetyl-2-deoxy-1-thio-β-D-glucopyranose (**14a**): white solid; (2.8 g, 65.9 % over three steps) yield; [α]_D_^25^ -15.8 (c=1.0, CHCl_3_); ^1^H NMR (300 MHz, DMSO-*d*_6_) δ 8.60 (d, *J* = 9.4 Hz, 1H, NH), 7.88 – 7.70 (m, 2H, ArH), 7.62 – 7.41 (m, 3H, ArH), 5.27 (t, *J* = 9.8 Hz, 1H, H-3), 4.97 (t, *J* = 9.7 Hz, 2H, H-4, H-1), 4.21 (dd, *J* = 12.4, 4.8 Hz, 1H, H-6b), 4.17 – 4.01 (m, 2H, H-6a, H-2), 3.93 – 3.84 (m, 1H, H-5), 3.47 (d, *J* = 8.3 Hz, 1H, SH), 2.05 (s, 3H, OAc), 2.00 (s, 3H, OAc), 1.87 (s, 3H, OAc); ^13^C NMR (75 MHz, DMSO-*d*_6_) δ 170.19, 169.80, 169.36, 166.67, 134.43, 131.55, 128.48, 127.68, 127.33, 127.24, 78.91, 75.40, 73.65, 68.60, 62.06, 56.21, 20.64, 20.51, 20.37; HRMS (ESI) calcd for C_19_H_24_NO_8_S (M+H^+^) 426.1223, found 426.1229.

2-(4-fluorobenzamido)-3,4,6-tri-O-acetyl-2-deoxy-1-thio-β-D-glucopyranose (**14b**): white solid; (2.9 g, 65.5 % over three steps) yield; [α]_D_^25^ -19.6 (c=1.0, CHCl_3_); ^1^H NMR (300 MHz, DMSO-*d*_6_) δ 8.65 (d, *J* = 9.3 Hz, 1H, NH), 7.95 – 7.73 (m, 2H, ArH), 7.45 – 7.24 (m, 2H, ArH), 5.27 (t, *J* = 9.8 Hz, 1H, H-3), 5.05 – 4.92 (m, 2H, H-4, H-1), 4.22 (dd, *J* = 12.4, 4.8 Hz, 1H, H-6b), 4.17 – 4.01 (m, 2H, H-2, H-6a), 3.96 – 3.85 (m, 1H, H-5), 3.49 (d, *J* = 8.4 Hz, 1H, SH), 2.06 (s, 3H, OAc), 2.01 (s, 3H, OAc), 1.88 (s, 3H, OAc); ^13^C NMR (75 MHz, DMSO-*d*_6_) δ 170.19, 169.81, 169.37, 165.50, 130.84, 130.81, 129.95, 129.83, 115.59, 115.30, 78.86, 75.18, 73.62, 68.49, 62.15, 56.28, 20.63, 20.48, 20.35; HRMS (ESI) calcd for C_19_H_23_FNO_8_S (M+H^+^) 444.1128, found 444.1117.

2-[[(phenylmethoxy) carbonyl] amino]-3,4,6-tri-O-acetyl-2-deoxy-1-thio-β-D-glucopyranose (**14c**): white solid; (2.3g, 61.5 % over three steps) yield; [α]_D_^25^ -22.1 (c=1.0, CHCl_3_); ^1^H NMR (300 MHz, CDCl_3_) δ 7.41 – 7.29 (m, 5H, ArH), 5.25 – 5.03 (m, 5H, H-3, PhCH_2_, H-4, H-1), 4.61 (t, *J* = 9.5 Hz, 1H, NH), 4.24 (dd, *J* = 12.4, 4.9 Hz, 1H, H-6b), 4.11 (dd, *J* = 12.4, 1.8 Hz, 1H, H-6a), 3.83 – 3.62 (m, 2H, H-2, H-5), 2.49 (d, *J* = 9.2 Hz, 1H, SH), 2.09 (s, 3H, OAc), 2.01 (s, 3H, OAc), 1.92 (s, 3H, OAc).

2-propionamido-3,4,6-tri-O-acetyl-2-deoxy-1-thio-β-D-glucopyranose (**14d**): white solid; (2.3g, 61.5 % over three steps) yield; [α]_D_^25^ -20.4 (c=1.0, CHCl_3_); ^1^H NMR (300 MHz, DMSO-*d*_6_) δ 7.94 (d, *J* = 9.4 Hz, 1H, NH), 5.06 (t, *J* = 9.8 Hz, 1H, H-3), 4.91 – 4.74 (m, 2H, H-4, H-1), 4.14 (dd, *J* = 12.4, 4.9 Hz, 1H, H-6b), 3.99 (dd, *J* = 12.4, 2.0 Hz, 1H, H-6a), 3.88 – 3.74 (m, 2H, H-5, H-2), 3.35 (d, J = 7.9 Hz, 1H, SH), 2.09 – 1.99 (m, 5H, CH_2_, OAc), 1.97 (d, *J* = 7.0 Hz, 3H, OAc), 1.89 (s, 3H, OAc), 0.96 (t, *J* = 7.6 Hz, 3H, CH_3_); ^13^C NMR (75 MHz, DMSO-*d*_6_) δ 173.18, 170.16, 169.69, 169.34, 78.84, 74.96, 73.51, 68.53, 62.14, 55.53, 28.95, 20.64, 20.49, 20.41, 10.02.

2-butyramido-3,4,6-tri-O-acetyl-2-deoxy-1-thio-β-D-glucopyranose (**14e**): white solid; (2.2 g, 56.2 % over three steps) yield; [α]_D_^25^ -28.3 (c=1.0, CHCl_3_); ^1^H NMR (300 MHz, DMSO-*d*_6_) δ 7.98 (d, *J* = 9.4 Hz, 1H, NH), 5.06 (t, *J* = 9.8 Hz, 1H, H-3), 4.93 – 4.70 (m, 2H, H-4, H-1), 4.14 (dd, *J* = 12.4, 4.9 Hz, 1H, H-6b), 3.99 (dd, *J* = 12.4, 2.0 Hz, 1H, H-6a), 3.91 – 3.76 (m, 2H, H-5, H-2), 3.33 (s, 1H, SH), 2.01 (s, 3H, OAc), 2.01 – 1.97 (m, 2H, CH_2_), 1.95 (s, 3H, OAc), 1.89 (s, 3H, OAc), 1.61 – 1.36 (m, 2H, CH_2_), 0.82 (t, *J* = 7.4 Hz, 3H, CH_3_); ^13^C NMR (75 MHz, DMSO-*d*_6_) δ 172.22, 170.17, 169.65, 169.34, 87.58, 78.93, 74.94, 73.46, 68.62, 62.14, 37.66, 20.63, 20.54, 20.47, 18.74, 13.54; HRMS (ESI) calcd for C_16_H_26_NO_8_S (M+H^+^) 392.1379, found 392.1371.

2-isobutyramido-3,4,6-tri-O-acetyl-2-deoxy-1-thio-β-D-glucopyranose (**14f**): white solid; (2.5 g, 63.9 % over three steps) yield; [α]_D_^25^ -25.9 (c=1.0, CHCl_3_);^1^H NMR (300 MHz, CDCl_3_) δ 6.10 (d, *J* = 9.5 Hz, 1H, NH), 5.24 – 5.03 (m, 2H, H-3, H-4), 4.66 (t, *J* = 9.7 Hz, 1H, H-1), 4.25 (dd, *J* = 12.4, 4.9 Hz, 1H, H-6b), 4.20 – 4.03 (m, 2H, H-2, H-6a), 3.74 (ddd, *J* = 9.5, 4.8, 2.2 Hz, 1H, H-5), 2.59 (d, *J* = 9.4 Hz, 1H, SH), 2.44 – 2.29 (m, 1H, CHCH_3_), 2.11 (s, 3H, OAc), 2.03 (s, 6H, 2 OAc), 1.14 (t, *J* = 6.6 Hz, 6H, 2 CH_3_); ^13^C NMR (75 MHz, CDCl_3_) δ 176.97, 170.63, 170.36, 168.87, 79.86, 75.87, 73.14, 67.98, 61.93, 56.04, 35.35, 20.40, 20.26, 20.22, 19.04, 18.87; HRMS (ESI) calcd for C_16_H_26_NO_8_S (M+H^+^) 392.1379, found 392.1363.

2-[[(methylamino)carbonyl]amino]- 3,4,6- tri- O- acetyl-2- deoxy -1- thio- β-D- glucopyranose (**14g**): white solid; (2.0 g, 52.9 % over three steps) yield; [α]_D_^25^ -17.9 (c=1.0, CHCl_3_); ^1^H NMR (300 MHz, DMSO-*d*_6_) δ 5.96 (s, 1H, NH), 5.89 (d, *J* = 5.1 Hz, 1H, NH), 5.20 – 5.03 (m, 3H, H-3, H-4, H-1), 4.15 (dd, *J* = 12.2, 4.3 Hz, 1H, H-6b), 4.05 – 3.76 (m, 3H, H-6a, H-2, H-5), 3.26 (s, 1H, SH), 2.48 (d, *J* = 2.1 Hz, 3H, NCH_3_), 1.98 (s, 3H, OAc), 1.94 (s, 3H, OAc), 1.88 (s, 3H, OAc); ^13^C NMR (75 MHz, DMSO-*d*_6_) δ 172.78, 170.11, 169.60, 161.24, 78.81, 75.03, 73.44, 68.51, 62.07, 55.42, 26.37, 20.62, 20.48, 20.43; HRMS (ESI) calcd for C_14_H_23_N_2_O_8_S (M+H^+^) 379.1175, found 379.1187.

2-[[(2,2,2-trichloroethoxy)carbonyl]amino]-3,4,6-tri-O-acetyl-2-deoxy-1-thio-β-D-glucopyranose (**14h**): white solid; (3.0 g, 60.4 % over three steps) yield; [α]_D_^25^ -20.4 (c=1.0, CHCl_3_); ^1^H NMR (300 MHz, CDCl_3_) δ 5.59 (d, *J* = 9.5 Hz, 1H, NH), 5.20 (t, *J* = 9.7 Hz, 1H, H-3), 5.10 (t, *J* = 9.6 Hz, 1H, H-4), 4.84 – 4.60 (m, 3H, H-1, CCl_3_CH_2_), 4.26 (dd, *J* = 12.4, 4.8 Hz, 1H, H-6b), 4.14 (dd, *J* = 12.4, 2.1 Hz, 1H, H-6a), 3.87 – 3.68 (m, 2H, H-2, H-5), 2.49 (br s, 1H, SH), 2.11 (s, 3H, OAc), 2.04 (s, 6H, 2 OAc); ^13^C NMR (75 MHz, CDCl_3_) δ 169.85, 169.76, 168.39, 153.48, 94.35, 79.08, 75.26, 73.57, 72.07, 67.42, 61.22, 58.00, 19.78, 19.64, 19.61.

2-trifluoroacetamido-3,4,6-tri-O-acetyl-2-deoxy-1-thio-β-D-glucopyranose (**14i**): white solid; (2.6 g, 62.3 % over three steps) yield; [α]_D_^25^ -63.5 (c=1.0, CHCl_3_); ^1^H NMR (300 MHz, DMSO-*d*_6_) δ 9.67 (d, *J* = 9.2 Hz, 1H, NH), 5.15 (t, *J* = 9.8 Hz, 1H, H-3), 4.98 – 4.81 (m, 2H, H-4, H-1), 4.16 (dd, *J* = 12.5, 4.9 Hz, 1H, H-6b), 4.01 (dd, *J* = 12.5, 1.7 Hz, 1H, H-6a), 3.97 – 3.82 (m, 2H, H-2, H-5), 3.75 (d, *J* = 7.5 Hz, 1H, SH), 2.02 (s, 3H, OAc), 1.97 (s, 3H, OAc), 1.91 (s, 3H, OAc); ^13^C NMR (75 MHz, DMSO-*d*_6_) δ 170.15, 169.70, 169.30, 156.62, 117.67, 113.85, 85.35, 75.43, 72.74, 68.25, 62.01, 52.58, 20.58, 20.42, 20.12.

A solution of **14a-14i** (2 mmol, 1.0 eq) in acetone (60 mL) and H_2_O (30 mL) was mixed with solid potassium carbonate (2.4 mmol, 1.2 eq), then **6a** (or **6d**) (2 mmol, 1 eq) was added. The mixture was stirred for 5 h at room temperature until TLC (petroleum ether/EtOAc, 1:2 v/v) indicated that the reaction was complete. After the solution was concentrated *in vacuo*, the residue was diluted with DCM (80 mL), washed with H_2_O (100 mL), brine (100 mL), dried over Na_2_SO_4_, and concentrated. Finally, the residue was purified by flash column chromatography (petroleum ether/EtOAc, 2:1 v/v) to afford **15a-15p**.

*N*-[3-[6-(piperidin-1-yl)-1,3-dioxo-1*H*-benzo[*de*]isoquinolin-2(3*H*)-yl]propyl]-2-[(2-benzamido-3,4,6- tri-O-acetyl-β-D-glucopyranosyl)thio]acetamide (**15a**): yellow solid; (1.4 g, 87.5 %) yield; [α]_D_^25^ -55.7 (c=0.2, DMSO); ^1^H NMR (300 MHz, DMSO-*d*_6_) δ 8.56 (d, *J* = 9.3 Hz, 1H, ArH), 8.47 – 8.28 (m, 3H, ArH), 7.99 (t, *J* = 5.7 Hz, 1H, NH), 7.86 – 7.68 (m, 3H, ArH, NH), 7.55 – 7.36 (m, 3H, ArH), 7.26 (d, *J* = 8.2 Hz, 1H, ArH), 5.32 (t, *J* = 9.7 Hz, 1H, H-3), 5.09 – 4.89 (m, 2H, H-4, H-1), 4.34 – 4.14 (m, 2H, H-6b, H-2), 4.12 – 3.95 (m, 3H, CH_2_Ar, H-6a), 3.92 – 3.81 (m, 1H, H-5), 3.37 – 3.26 (m, 2H, SCH_2_), 3.26 – 3.06 (m, 6H, 3 CH_2_), 2.02 (s, 3H, OAc), 2.00 (s, 3H, OAc), 1.87 (s, 3H, OAc), 1.86 – 1.71 (m, 6H, 3 CH_2_), 1.71 – 1.59 (m, 2H, CH_2_); ^13^C NMR (75 MHz, DMSO-*d*_6_) δ 170.15, 169.78, 169.39, 168.35, 166.52, 163.74, 163.19, 156.84, 134.32, 132.39, 131.44, 130.69, 129.29, 128.38, 127.24, 125.85, 125.53, 122.59, 115.08, 114.97, 83.10, 75.03, 73.80, 68.65, 62.09, 54.08, 52.79, 37.59, 37.03, 32.90, 28.03, 25.83, 23.97, 20.58, 20.53, 20.40; HRMS (ESI) calcd for C_41_H_47_N_4_O_11_S (M+H^+^) 803.2962, found 803.2968.

*N*-[3-[6-(piperidin-1-yl)-1,3-dioxo-1*H*-benzo[*de*]isoquinolin-2(3*H*)-yl]propyl]-2-[(2-(4-fluorobenzamido)-3,4,6- tri-O-acetyl-β-D-glucopyranosyl)thio]acetamide (**15b**): yellow solid; (1.5 g, 91.4 %) yield; [α]_D_^25^ -60.4 (c=0.2, DMSO); ^1^H NMR (300 MHz, DMSO-*d*_6_) δ 8.59 (d, *J* = 9.4 Hz, 1H, ArH), 8.49 – 8.33 (m, 3H, ArH), 7.97 (t, *J* = 5.5 Hz, 1H, NH), 7.85 – 7.73 (m, 3H, ArH, NH), 7.34 – 7.16 (m, 3H, ArH), 5.29 (t, *J* = 9.8 Hz, 1H, H-3), 5.03 – 4.91 (m, 2H, H-4, H-1), 4.32 – 4.11 (m, 2H, H-6b, H-2), 4.09 – 3.96 (m, 3H, CH_2_Ar, H-6a), 3.93 – 3.79 (m, 1H, H-5), 3.34 – 3.25 (m, 2H, SCH_2_), 3.24 – 3.17 (m, 4H, 2 CH_2_), 3.12 (dd, *J* = 12.8, 6.7 Hz, 2H, CH_2_), 2.00 (s, 3H, OAc), 1.99 (s, 3H, OAc), 1.86 (s, 3H, OAc), 1.85 – 1.71 (m, 6H, 3 CH_2_), 1.70 – 1.60 (m, 2H, CH_2_); ^13^C NMR (75 MHz, DMSO-*d*_6_) δ 170.16, 169.80, 169.39, 168.34, 165.44, 163.78, 163.23, 156.90, 132.42, 130.74, 129.94, 129.82, 129.33, 125.91, 125.58, 122.63, 115.53, 115.24, 115.11, 115.02, 83.03, 75.01, 73.75, 68.60, 62.08, 54.09, 52.84, 37.59, 37.01, 32.85, 28.03, 25.83, 23.97, 20.58, 20.54, 20.40; HRMS (ESI) calcd for C_41_H_46_FN_4_O_11_S (M+H^+^) 821.2868, found 821.2879.

*N*-[3-[6-(piperidin-1-yl)-1,3-dioxo-1*H*-benzo[*de*]isoquinolin-2(3*H*)-yl]propyl]-2-[[2-[[(phenylmethoxy)carbonyl]amino]-3,4,6- tri-O-acetyl-β-D-glucopyranosyl]thio]acetamide (**15c**): yellow solid; (1.4 g, 84.3 %) yield; [α]_D_^25^ -47.1 (c=0.2, DMSO); ^1^H NMR (300 MHz, DMSO-*d*_6_) δ 8.45 – 8.36 (m, 1H, ArH), 8.31 (d, *J* = 8.1 Hz, 2H, ArH), 7.96 (t, *J* = 5.6 Hz, 1H, NH), 7.77 – 7.66 (m, 1H, ArH), 7.51 (d, *J* = 9.6 Hz, 1H, NH), 7.38 – 7.22 (m, 5H, ArH), 7.20 (d, *J* = 8.2 Hz, 1H, ArH), 5.12 (t, *J* = 8.3 Hz, 1H, H-3), 5.09 – 4.85 (m, 3H, H-4, OCH_2_), 4.80 (d, *J* = 10.4 Hz, 1H, H-1), 4.18 (dd, *J* = 12.3, 4.7 Hz, 1H, H-6b), 4.08 – 3.96 (m, 3H, CH_2_Ar, H-6a), 3.86 – 3.75 (m, 1H, H-5), 3.66 (q, *J* = 10.0 Hz, 1H, H-2), 3.33 – 3.21 (m, 2H, SCH_2_), 3.20 – 3.05 (m, 6H, 3 CH_2_), 1.97 (s, 3H, OAc), 1.95 (s, 3H, OAc), 1.84 (s, 3H, OAc), 1.83 – 1.69 (m, 6H, 3 CH_2_), 1.68 – 1.55 (m, 2H, CH_2_); ^13^C NMR (75 MHz, DMSO-*d*_6_) δ 170.12, 169.61, 169.36, 168.28, 163.74, 163.19, 156.80, 155.91, 137.16, 132.37, 130.68, 130.63, 129.25, 128.36, 127.77, 127.41, 125.80, 125.49, 122.54, 115.04, 114.90, 83.11, 74.87, 73.86, 68.67, 65.49, 62.03, 54.52, 54.06, 37.63, 37.02, 32.87, 28.04, 25.83, 23.97, 20.55, 20.50, 20.36; HRMS (ESI) calcd for C_42_H_49_N_4_O_12_S (M+H^+^) 833.3068, found 833.3056.

*N*-[3-[6-(piperidin-1-yl)-1,3-dioxo-1*H*-benzo[*de*]isoquinolin-2(3*H*)-yl]propyl]-2-[(2-propionamido-3,4,6- tri-O-acetyl-β-D-glucopyranosyl)thio]acetamide (**15d**): yellow solid; (1.3 g, 86.1 %) yield; [α]_D_^25^ -40.5 (c=0.2, DMSO); ^1^H NMR (300 MHz, DMSO-*d*_6_) δ 8.43 (d, *J* = 7.1 Hz, 1H, ArH), 8.34 (dd, *J* = 8.2, 2.5 Hz, 2H, ArH), 8.05 – 7.89 (m, 2H, 2 NH), 7.81 – 7.70 (m, 1H, ArH), 7.25 (d, *J* = 8.2 Hz, 1H, ArH), 5.13 (t, *J* = 9.7 Hz, 1H, H-3), 4.91 (t, *J* = 9.7 Hz, 1H, H-4), 4.82 (d, *J* = 10.4 Hz, 1H, H-1), 4.20 (dd, *J* = 12.3, 4.7 Hz, 1H, H-6b), 4.10 – 4.00 (m, 3H, CH_2_Ar, H-6a), 4.00 – 3.89 (m, 1H, H-2), 3.88 – 3.80 (m, 1H, H-5), 3.35 – 3.21 (m, 2H, SCH_2_), 3.20 – 3.05 (m, 6H, 3 CH_2_), 2.09 – 2.01 (m, 2H, CH_2_), 2.00 (s, 3H, OAc), 1.98 (s, 3H, OAc), 1.93 (s, 3H, OAc), 1.88 – 1.72 (m, 6H, 3 CH_2_), 1.71 – 1.59 (m, 2H, CH_2_), 0.95 (t, *J* = 7.6 Hz, 3H, CH_3_); ^13^C NMR (75 MHz, DMSO-*d*_6_) δ 173.14, 170.13, 169.71, 169.36, 168.34, 163.73, 163.18, 156.82, 132.36, 130.67, 129.26, 125.83, 125.50, 122.56, 115.05, 114.94, 83.00, 74.85, 73.75, 68.64, 62.06, 54.07, 52.11, 37.62, 37.00, 32.84, 28.90, 28.05, 25.82, 23.96, 20.56, 20.51, 20.42, 9.99; HRMS (ESI) calcd for C_37_H_47_N_4_O_11_S (M+H^+^) 755.2962, found 755.2971.

*N*-[3-[6-(piperidin-1-yl)-1,3-dioxo-1*H*-benzo[*de*]isoquinolin-2(3*H*)-yl]propyl]-2-[(2-butyramido-3,4,6- tri-O-acetyl-β-D-glucopyranosyl)thio]acetamide (**15e**): yellow solid; (1.3 g, 84.6 %) yield; [α]_D_^25^ -49.3 (c=0.2, DMSO); ^1^H NMR (300 MHz, DMSO-*d*_6_) δ 8.41 (d, *J* = 7.2 Hz, 1H, ArH), 8.33 (dd, *J* = 8.2, 3.0 Hz, 2H, ArH), 7.95 – 7.81 (m, 2H, 2 NH), 7.78 – 7.68 (m, 1H, ArH), 7.23 (d, *J* = 8.2 Hz, 1H, ArH), 5.11 (t, *J* = 9.7 Hz, 1H, H-3), 4.87 (t, *J* = 9.7 Hz, 1H, H-4), 4.80 (d, *J* = 10.4 Hz, 1H, H-1), 4.16 (dd, *J* = 12.4, 4.8 Hz, 1H, H-6b), 4.06 – 3.87 (m, 4H, CH_2_Ar, H-6a, H-2), 3.85 – 3.74 (m, 1H, H-5), 3.29 – 3.21 (m, 2H, SCH_2_), 3.20 – 3.06 (m, 6H, 3 CH_2_), 2.01 – 1.95 (m, 5H, CH_2_, OAc), 1.94 (s, 3H, OAc), 1.88 (s, 3H, OAc), 1.83 – 1.69 (m, 6H, 3 CH_2_), 1.68 – 1.55 (m, 2H, CH_2_), 1.53 – 1.31 (m, 2H, CH_2_), 0.76 (t, *J* = 7.4 Hz, 3H, CH_3_); ^13^C NMR (75 MHz, DMSO-*d*_6_) δ 172.16, 170.13, 169.64, 169.37, 168.37, 163.74, 163.19, 156.83, 132.38, 130.68, 129.27, 125.84, 125.51, 122.56, 115.05, 114.95, 83.07, 74.83, 73.70, 68.72, 62.06, 54.07, 51.97, 37.62, 37.00, 32.83, 28.05, 25.83, 23.96, 20.56, 20.51, 20.46, 18.70, 13.50; HRMS (ESI) calcd for C_38_H_49_N_4_O_11_S (M+H^+^) 769.3119, found 769.3112.

*N*-[3-[6-(piperidin-1-yl)-1,3-dioxo-1*H*-benzo[*de*]isoquinolin-2(3*H*)-yl]propyl]-2-[(2-isobutyramido-3,4,6- tri-O-acetyl-β-D-glucopyranosyl)thio]acetamide (**15f**): yellow solid; (1.4 g, 91.1 %) yield; [α]_D_^25^ -68.8 (c=0.2, DMSO); ^1^H NMR (300 MHz, DMSO-*d*_6_) δ 8.40 (d, *J* = 7.2 Hz, 1H, ArH), 8.31 (dd, *J* = 8.2, 2.9 Hz, 2H, ArH), 7.93 (t, *J* = 5.6 Hz, 1H, NH), 7.85 (d, *J* = 9.5 Hz, 1H, NH), 7.77 – 7.66 (m, 1H, ArH), 7.21 (d, *J* = 8.2 Hz, 1H, ArH), 5.11 (t, *J* = 9.7 Hz, 1H, H-3), 4.87 (t, *J* = 9.8 Hz, 1H, H-4), 4.80 (d, *J* = 10.4 Hz, 1H, H-1), 4.16 (dd, *J* = 12.2, 4.6 Hz, 1H, H-6b), 4.07 – 3.96 (m, 3H, CH_2_Ar, H-6a), 3.95 – 3.84 (m, 1H, H-2), 3.84 – 3.73 (m, 1H, H-5), 3.32 – 3.17 (m, 2H, SCH_2_), 3.17 – 3.04 (m, 6H, 3 CH_2_), 2.30 – 2.14 (m, 1H, CHCH_3_), 1.96 (s, 3H, OAc), 1.94 (s, 3H, OAc), 1.87 (s, 3H, OAc), 1.83 – 1.68 (m, 6H, 3 CH_2_), 1.67 – 1.54 (m, 2H, CH_2_), 0.90 (dd, *J* = 6.8, 4.4 Hz, 6H, 2 CH_3_); ^13^C NMR (75 MHz, DMSO-*d*_6_) δ 176.16, 170.13, 169.67, 169.35, 168.38, 163.73, 163.18, 156.82, 132.37, 130.68, 129.26, 125.83, 125.51, 122.56, 115.05, 114.94, 83.02, 74.89, 73.74, 68.63, 62.07, 54.07, 51.93, 37.61, 37.00, 34.39, 32.80, 28.05, 25.83, 23.96, 20.55, 20.51, 20.41, 19.54, 19.08; HRMS (ESI) calcd for C_38_H_49_N_4_O_11_S (M+H^+^) 769.3119, found 769.3132.

*N*-[3-[6-(piperidin-1-yl)-1,3-dioxo-1*H*-benzo[*de*]isoquinolin-2(3*H*)-yl]propyl]-2-[(2-trifluoroacetamido-3,4,6- tri-O-acetyl-β-D-glucopyranosyl)thio]acetamide (**15g**): yellow solid; (1.3 g, 82.3 %) yield; [α]_D_^25^ -62.1 (c=0.2, DMSO); ^1^H NMR (300 MHz, DMSO-*d*_6_) δ 9.68 (d, *J* = 9.1 Hz, 1H, NH), 8.48 – 8.25 (m, 3H, ArH), 7.99 (t, *J* = 5.6 Hz, 1H, NH), 7.73 (dd, *J* = 8.4, 7.4 Hz, 1H, ArH), 7.21 (d, *J* = 8.2 Hz, 1H, ArH), 5.20 (t, *J* = 9.7 Hz, 1H, H-3), 5.02 – 4.82 (m, 2H, H-4, H-1), 4.17 (dd, *J* = 12.4, 4.8 Hz, 1H, H-6b), 4.08 – 3.91 (m, 4H, CH_2_Ar, H-6a, H-2), 3.89 – 3.75 (m, 1H, H-5), 3.27 (dd, *J* = 37.7, 10.1 Hz, 2H, SCH_2_), 3.17 – 3.05 (m, 6H, 3 CH_2_), 1.96 (s, 6H, 2 OAc), 1.90 (s, 3H, OAc), 1.84 – 1.68 (m, 6H, 3 CH_2_), 1.67 – 1.55 (m, 2H, CH_2_); ^13^C NMR (75 MHz, DMSO-*d*_6_) δ 170.10, 169.62, 169.35, 168.18, 163.73, 163.18, 156.81, 132.34, 130.65, 129.26, 125.82, 125.52, 122.57, 117.70, 115.07, 114.93, 113.88, 81.84, 75.03, 73.03, 68.33, 61.98, 54.06, 52.90, 37.58, 37.05, 32.70, 28.00, 25.82, 23.96, 20.53, 20.48, 20.18; HRMS (ESI) calcd for C_36_H_42_F_3_N_4_O_11_S (M+H^+^) 795.2523, found 795.2540.

*N*-[3-[6-(piperidin-1-yl)-1,3-dioxo-1*H*-benzo[*de*]isoquinolin-2(3*H*)-yl]propyl]-2-[[2-[[(methylamino)carbonyl]amino]-3,4,6- tri-O-acetyl-β-D-glucopyranosyl]thio]acetamide (**15h**): yellow solid; (1.2 g, 79.5 %) yield; [α]_D_^25^ -69.2 (c=0.2, DMSO); ^1^H NMR (300 MHz, DMSO-*d*_6_) δ 8.41 (d, *J* = 7.2 Hz, 1H, ArH), 8.32 (d, *J* = 8.2 Hz, 2H, ArH), 7.96 (t, *J* = 5.6 Hz, 1H, NH), 7.77 – 7.67 (m, 1H, ArH), 7.22 (d, *J* = 8.2 Hz, 1H, ArH), 6.01 – 5.84 (m, 2H, 2 NH), 5.13 (t, *J* = 9.7 Hz, 1H, H-3), 4.93 – 4.70 (m, 2H, H-4, H-1), 4.15 (dd, *J* = 12.3, 4.8 Hz, 1H, H-6b), 4.04 – 3.94 (m, 3H, CH_2_Ar, H-6a), 3.86 – 3.71 (m, 2H, H-2, H-5), 3.33 – 3.18 (m, 2H, SCH_2_), 3.18 – 3.04 (m, 6H, 3 CH_2_), 2.49 (d, *J* = 4.6 Hz, 3H, NCH_3_), 1.96 (s, 3H, OAc), 1.94 (s, 3H, OAc), 1.89 (s, 3H, OAc), 1.84 – 1.68 (m, 6H, 3 CH_2_), 1.67 – 1.54 (m, 2H, CH_2_); ^13^C NMR (75 MHz, DMSO-*d*_6_) δ 170.15, 169.80, 169.38, 168.45, 163.75, 163.20, 157.94, 156.83, 132.41, 130.72, 130.66, 129.27, 125.85, 125.51, 122.57, 115.07, 114.95, 83.74, 74.67, 74.14, 68.90, 62.12, 54.07, 53.24, 37.65, 36.99, 32.78, 28.07, 26.49, 25.83, 23.97, 20.56, 20.55, 20.53; HRMS (ESI) calcd for C_36_H_46_N_5_O_11_S (M+H^+^) 756.2915, found 756.2918.

*N*-[3-[6-(piperidin-1-yl)-1,3-dioxo-1*H*-benzo[*de*]isoquinolin-2(3*H*)-yl]propyl]-2-[[2-[[(2,2,2-trichloroethoxy)carbonyl]amino]-3,4,6- tri-O-acetyl-β-D-glucopyranosyl]thio]acetamide (**15i**): yellow solid; (1.4 g, 80.1 %) yield; [α]_D_^25^ -58.6 (c=0.2, DMSO); ^1^H NMR (300 MHz, DMSO-*d*_6_) δ 8.38 (dd, *J* = 7.2, 0.9 Hz, 1H, ArH), 8.29 (d, *J* = 8.1 Hz, 2H, ArH), 8.02 – 7.88 (m, 2H, 2 NH), 7.71 (dd, *J* = 8.4, 7.4 Hz, 1H, ArH), 7.19 (d, *J* = 8.2 Hz, 1H, ArH), 5.13 (t, *J* = 9.7 Hz, 1H, H-3), 4.95 – 4.66 (m, 4H, H-4, H-1, CH_2_O), 4.16 (dd, *J* = 12.3, 4.7 Hz, 1H, H-6b), 4.06 – 3.93 (m, 3H, CH_2_Ar, H-6a), 3.86 – 3.74 (m, 1H, H-5), 3.66 (dd, *J* = 19.9, 10.0 Hz, 1H, H-2), 3.32 – 3.18 (m, 2H, SCH_2_), 3.17 – 3.04 (m, 6H, 3 CH_2_), 1.96 (s, 3H, OAc), 1.95 (s, 3H, OAc), 1.88 (s, 3H, OAc), 1.82 – 1.70 (m, 6H, 3 CH_2_), 1.67 – 1.53 (m, 2H, CH_2_); ^13^C NMR (75 MHz, DMSO-*d*_6_) δ 170.11, 169.55, 169.37, 168.25, 163.73, 163.18, 156.80, 154.35, 132.37, 130.67, 130.63, 129.25, 125.79, 125.49, 122.53, 115.03, 114.90, 96.22, 82.90, 74.89, 73.62, 73.52, 68.64, 62.00, 54.64, 54.06, 37.63, 37.03, 32.81, 28.03, 25.82, 23.97, 20.54, 20.49, 20.45; HRMS (ESI) calcd for C_37_H_44_Cl_3_N_4_O_12_S (M+H^+^) 873.1742, found 873.1726.

*N*-[3-[6-bromo-1,3-dioxo-1*H*-benzo[*de*]isoquinolin-2(3*H*)-yl]propyl]-2-[(2-benzamido-3,4,6-tri-O-acetyl-β-D-glucopyranosyl)thio]acetamide (**15j**): yellow solid; (1.3 g, 81.8 %) yield; [α]_D_^25^ -43.9 (c=0.2, DMSO); ^1^H NMR (300 MHz, DMSO-*d*_6_) δ 8.61 – 8.42 (m, 3H, ArH), 8.25 (d, *J* = 7.9 Hz, 1H, ArH), 8.14 (d, *J* = 7.9 Hz, 1H, ArH), 8.02 – 7.88 (m, 2H, 2 NH), 7.75 – 7.67 (m, 2H, ArH), 7.45 – 7.35 (m, 3H, ArH), 5.30 (t, *J* = 9.7 Hz, 1H, H-3), 5.03 – 4.90 (m, 2H, H-4, H-1), 4.30 – 4.15 (m, 2H, H-6b, H-2), 4.09 – 3.95 (m, 3H, CH_2_Ar, H-6a), 3.89 – 3.78 (m, 1H, H-5), 3.38 – 3.19 (m, 2H, SCH_2_), 3.13 (dd, *J* = 12.9, 6.6 Hz, 2H, CH_2_), 1.99 (s, 3H, OAc), 1.98 (s, 3H, OAc), 1.85 (s, 3H, OAc), 1.82 – 1.70 (m, 2H, CH_2_); ^13^C NMR (75 MHz, DMSO-*d*_6_ ) δ 170.16, 169.79, 169.39, 168.39, 166.52, 163.01, 162.97, 134.32, 132.69, 131.66, 131.45, 131.41, 131.04, 129.87, 129.22, 128.84, 128.40, 127.24, 122.84, 122.07, 83.10, 75.02, 73.78, 68.66, 62.12, 52.77, 38.00, 37.02, 32.90, 27.84, 20.59, 20.54, 20.40; HRMS (ESI) calcd for C_36_H_37_BrN_3_O_11_S (M+H^+^) 798.1332, found 798.1345.

*N*-[3-[6-bromo-1,3-dioxo-1*H*-benzo[*de*]isoquinolin-2(3*H*)-yl]propyl]-2-[(2-(4-fluorobenzamido)-3,4,6-tri-O-acetyl-β-D-glucopyranosyl)thio]acetamide (**15k**): yellow solid; (1.4 g, 85.9 %) yield; [α]_D_^25^ -45.6 (c=0.2, DMSO); ^1^H NMR (300 MHz, DMSO-*d*_6_) δ 8.59 (d, *J* = 9.4 Hz, 1H, ArH), 8.51 – 8.38 (m, 2H, ArH), 8.23 (d, *J* = 7.9 Hz, 1H, ArH), 8.11 (d, *J* = 7.9 Hz, 1H, ArH), 7.98 (t, *J* = 5.6 Hz, 1H, NH), 7.94 – 7.86 (m, 1H, NH), 7.78 (dd, *J* = 8.7, 5.5 Hz, 2H, ArH), 7.23 (t, *J* = 8.8 Hz, 2H, ArH), 5.29 (t, *J* = 9.7 Hz, 1H, H-3), 5.03 – 4.89 (m, 2H, H-4, H-1), 4.28 – 4.10 (m, 2H, H-6b, H-2), 4.09 – 3.91 (m, 3H, CH_2_Ar, H-6a), 3.91 – 3.79 (m, 1H, H-5), 3.39 – 3.19 (m, 2H, SCH_2_), 3.13 (dd, *J* = 12.6, 6.5 Hz, 2H, CH_2_), 1.99 (s, 3H, OAc), 1.98 (s, 3H, OAc), 1.85 (s, 3H, OAc), 1.81 – 1.70 (m, 2H, CH_2_); ^13^C NMR (75 MHz, DMSO-*d*_6_) δ 170.15, 169.80, 169.39, 168.39, 165.44, 162.98, 162.93, 132.66, 131.62, 131.37, 131.00, 130.70, 129.95, 129.83, 129.21, 128.79, 128.32, 122.78, 122.01, 115.51, 115.22, 83.06, 75.02, 73.75, 68.63, 62.11, 52.85, 38.00, 37.02, 32.88, 27.85, 20.58, 20.53, 20.39; HRMS (ESI) calcd for C_36_H_36_BrFN_3_O_11_S (M+H^+^) 816.1238, found 816.1247.

*N*-[3-[6-bromo-1,3-dioxo-1*H*-benzo[*de*]isoquinolin-2(3*H*)-yl]propyl]-2-[(2-propionamido-3,4,6- tri-O-acetyl-β-D-glucopyranosyl)thio]acetamide (**15l**): yellow solid; (1.3 g, 86.6 %) yield; [α]_D_^25^ -50.7 (c=0.2, DMSO); ^1^H NMR (300 MHz, DMSO-*d*_6_) δ 8.57 – 8.43 (m, 2H, ArH), 8.27 (d, *J* = 7.9 Hz, 1H, ArH), 8.15 (d, *J* = 7.9 Hz, 1H, ArH), 8.03 – 7.86 (m, 3H, ArH, 2 NH), 5.10 (t, *J* = 9.7 Hz, 1H, H-3), 4.88 (t, *J* = 9.7 Hz, 1H, H-4), 4.79 (d, *J* = 10.4 Hz, 1H, H-1), 4.16 (dd, *J* = 12.4, 4.7 Hz, 1H, H-6b), 4.07 – 3.87 (m, 4H, CH_2_Ar, H-6a, H-2), 3.86 – 3.77 (m, 1H, H-5), 3.38 – 3.20 (m, 2H, SCH_2_), 3.14 (dd, *J* = 13.4, 6.5 Hz, 2H, CH_2_), 2.05 – 1.97 (m, 5H, CH_2_CH_3_, OAc), 1.96 (s, 3H, OAc), 1.90 (s, 3H, OAc), 1.86 – 1.71 (m, 2H, CH_2_), 0.93 (t, *J* = 7.6 Hz, 3H, CH_3_); ^13^C NMR (75 MHz, DMSO-*d*_6_) δ 173.16, 170.14, 169.71, 169.37, 168.38, 163.05, 163.00, 132.70, 131.68, 131.43, 131.06, 129.88, 129.22, 128.86, 128.40, 122.87, 122.09, 83.02, 74.85, 73.73, 68.66, 62.10, 52.11, 38.05, 37.01, 32.86, 28.90, 27.87, 20.56, 20.52, 20.43, 10.00; HRMS (ESI) calcd for C_32_H_37_BrN_3_O_11_S (M+H^+^) 750.1332, found 750.1347.

*N*-[3-[6-bromo-1,3-dioxo-1*H*-benzo[*de*]isoquinolin-2(3*H*)-yl]propyl]-2-[(2-butyramido-3,4,6- tri-O-acetyl-β-D-glucopyranosyl)thio]acetamide (**15m**): yellow solid; (1.2 g, 78.4 %) yield; [α]_D_^25^ -53.5 (c=0.2, DMSO); ^1^H NMR (300 MHz, DMSO-*d*_6_) δ 8.52 – 8.39 (m, 2H, ArH), 8.24 (d, *J* = 7.9 Hz, 1H, ArH), 8.12 (d, *J* = 7.9 Hz, 1H, ArH), 8.01 – 7.87 (m, 3H, ArH, 2 NH), 5.11 (t, *J* = 9.8 Hz, 1H, H-3), 4.88 (t, *J* = 9.7 Hz, 1H, H-4), 4.79 (d, *J* = 10.4 Hz, 1H, H-1), 4.17 (dd, *J* = 12.3, 4.7 Hz, 1H, H-6b), 4.08 – 3.98 (m, 3H, CH_2_Ar, H-6a), 3.98 – 3.88 (m, 1H, H-2), 3.87 – 3.75 (m, 1H, H-5), 3.34 – 3.19 (m, 2H, SCH_2_), 3.14 (dd, *J* = 13.2, 6.4 Hz, 2H, CH_2_), 2.01 – 1.96 (m, 5H, OAc, CH_2_), 1.95 (s, 3H, OAc), 1.89 (s, 3H, OAc), 1.85 – 1.72 (m, 2H, CH_2_), 1.44 (dd, *J* = 14.6, 7.3 Hz, 2H, CH_2_), 0.77 (t, *J* = 7.4 Hz, 3H, CH_3_); ^13^C NMR (75 MHz, DMSO-*d*_6_) δ 172.18, 170.13, 169.64, 169.37, 168.40, 163.00, 162.95, 132.66, 131.64, 131.38, 131.02, 129.82, 129.21, 128.81, 128.32, 122.79, 122.01, 83.08, 74.84, 73.69, 68.75, 62.10, 51.97, 38.04, 37.62, 37.01, 32.84, 27.87, 20.56, 20.51, 20.45, 18.70, 13.51; HRMS (ESI) calcd for C_33_H_39_BrN_3_O_11_S (M+H^+^) 764.1489, found 764.1498.

*N*-[3-[6-bromo-1,3-dioxo-1*H*-benzo[*de*]isoquinolin-2(3*H*)-yl]propyl]-2-[(2-isobutyramido-3,4,6- tri-O-acetyl-β-D-glucopyranosyl)thio]acetamide (**15n**): yellow solid; (1.3 g, 85.0 %) yield; [α]_D_^25^ -42.7 (c=0.2, DMSO); ^1^H NMR (300 MHz, DMSO-*d*_6_) δ 8.58 – 8.43 (m, 2H, ArH), 8.27 (d, *J* = 7.9 Hz, 1H, ArH), 8.15 (d, *J* = 7.9 Hz, 1H, ArH), 8.02 – 7.91 (m, 2H, 2 NH), 7.86 (d, *J* = 9.5 Hz, 1H, ArH), 5.12 (t, *J* = 9.8 Hz, 1H, H-3), 4.88 (t, *J* = 9.7 Hz, 1H, H-4), 4.80 (d, *J* = 10.4 Hz, 1H, H-1), 4.17 (dd, *J* = 12.4, 4.9 Hz, 1H, H-6b), 4.08 – 3.98 (m, 3H, CH_2_Ar, H-6a), 3.97 – 3.86 (m, 1H, H-2), 3.85 – 3.77 (m, 1H, H-5), 3.33 – 3.18 (m, 2H, SCH_2_), 3.18 – 3.07 (m, 2H, CH_2_), 2.32 – 2.16 (m, 1H, CHCH_3_), 1.97 (s, 3H, OAc), 1.96 (s, 3H, OAc), 1.89 (s, 3H, OAc), 1.78 (dt, *J* = 13.4, 6.8 Hz, 2H, CH_2_), 0.97 – 0.87 (m, 6H, 2 CH_3_); ^13^C NMR (75 MHz, DMSO-*d*_6_) δ 176.16, 170.13, 169.67, 169.35, 168.40, 163.05, 163.00, 132.70, 131.68, 131.42, 131.06, 129.89, 129.22, 128.86, 128.40, 122.87, 122.09, 83.02, 74.89, 73.73, 68.66, 62.10, 51.93, 38.03, 37.01, 34.38, 32.81, 27.88, 20.57, 20.52, 20.41, 19.55, 19.10; HRMS (ESI) calcd for C_33_H_39_BrN_3_O_11_S (M+H^+^) 764.1489, found 764.1471.

*N*-[3-[6-bromo-1,3-dioxo-1*H*-benzo[*de*]isoquinolin-2(3*H*)-yl]propyl]-2-[(2-trifluoroacetamido-3,4,6- tri-O-acetyl-β-D-glucopyranosyl)thio]acetamide (**15o**): yellow solid; (1.3 g, 82.3 %) yield; [α]_D_^25^ -47.4 (c=0.2, DMSO); ^1^H NMR (300 MHz, DMSO-*d*_6_) δ 9.67 (d, *J* = 9.0 Hz, 1H, NH), 8.54 – 8.39 (m, 2H, ArH), 8.24 (d, *J* = 7.9 Hz, 1H, ArH), 8.13 (d, *J* = 7.9 Hz, 1H, ArH), 8.00 (t, *J* = 5.6 Hz, 1H, NH), 7.92 (dd, *J* = 8.5, 7.4 Hz, 1H, ArH), 5.21 (t, *J* = 9.7 Hz, 1H, H-3), 5.03 – 4.83 (m, 2H, H-4, H-1), 4.17 (dd, *J* = 12.4, 4.8 Hz, 1H, H-6b), 4.08 – 3.95 (m, 4H, CH_2_Ar, H-6a, H-2), 3.90 – 3.78 (m, 1H, H-5), 3.40 – 3.20 (m, 2H, SCH_2_), 3.14 (dd, *J* = 13.0, 6.8 Hz, 2H, CH_2_), 1.97 (s, 3H, OAc), 1.97 (s, 3H, OAc), 1.91 (s, 3H, OAc), 1.85 – 1.69 (m, 2H, CH_2_); ^13^C NMR (75 MHz, DMSO-*d*_6_) δ 170.11, 169.62, 169.35, 168.22, 163.00, 162.95, 156.71, 156.22, 132.65, 131.62, 131.38, 131.00, 129.84, 129.20, 128.81, 128.34, 122.81, 122.04, 117.70, 113.87, 81.85, 75.03, 73.02, 68.33, 62.01, 52.89, 38.00, 37.05, 32.70, 27.82, 20.54, 20.48, 20.18; HRMS (ESI) calcd for C_31_H_32_BrF_3_N_3_O_11_S (M+H^+^) 790.0893, found 790.0876.

*N*-[3-[6-bromo-1,3-dioxo-1*H*-benzo[*de*]isoquinolin-2(3*H*)-yl]propyl]-2-[[2-[[(2,2,2-trichloroethoxy)carbonyl]amino]-3,4,6- tri-O-acetyl-β-D-glucopyranosyl]thio]acetamide (**15p**): yellow solid; (1.4 g, 80.5 %) yield; [α]_D_^25^ -57.1 (c=0.2, DMSO); ^1^H NMR (300 MHz, DMSO-*d*_6_) δ 8.41 (d, *J* = 7.2 Hz, 1H, ArH), 8.33 (d, *J* = 8.3 Hz, 1H, ArH), 8.15 (d, *J* = 7.9 Hz, 1H, ArH), 8.10 – 7.91 (m, 3H, ArH, 2 NH), 7.84 (t, *J* = 7.9 Hz, 1H, ArH), 5.14 (t, *J* = 9.7 Hz, 1H, H-3), 5.01 – 4.64 (m, 4H, H-4, H-1, CH_2_CCl_3_), 4.17 (dd, *J* = 11.1, 3.2 Hz, 1H, H-6b), 4.06 – 3.94 (m, 3H, H-6a, CH_2_Ar), 3.86 – 3.76 (m, 1H, H-5), 3.68 (q, *J* = 10.1 Hz, 1H, H-2), 3.42 – 3.21 (m, 2H, SCH_2_), 3.20 – 3.06 (m, 2H, CH_2_), 1.97 (s, 3H, OAc), 1.96 (s, 3H, OAc), 1.90 (s, 3H, OAc), 1.85 – 1.69 (m, 2H, CH_2_); ^13^C NMR (75 MHz, DMSO-*d*_6_) δ 170.10, 169.54, 169.35, 168.29, 162.82, 162.76, 154.34, 132.49, 131.51, 131.19, 130.85, 129.60, 129.17, 128.58, 128.05, 122.52, 121.74, 96.22, 82.90, 74.92, 73.63, 73.54, 68.69, 62.05, 54.64, 38.04, 37.06, 32.84, 27.85, 20.54, 20.48, 20.44; HRMS (ESI) calcd for C_32_H_34_BrCl_3_N_3_O_12_S (M+H^+^) 868.0112, found 868.0125.

A solution of **15a-15o** (1.0 mmol) was suspended in anhydrous MeOH (15 mL), and saturated solution of CH_3_NH_2_ in MeOH (10 mL) was then added. The reaction was stirred for 48 h at room temperature, until TLC (EtOAc/MeOH/H_2_O, 8:1:1 v/v/v) indicated that the reaction was complete. The mixture was concentrated *in vacuo* and recrystallized from MeOH/ether to obtain **16a-16o**.

*N*-[3-[6-(piperidin-1-yl)-1,3-dioxo-1*H*-benzo[*de*]isoquinolin-2(3*H*)-yl]propyl]-2-[(2-benzamido-β-D-glucopyranosyl)thio]acetamide (**16a**): yellow solid; (0.55 g, 81.2 %) yield; [α]_D_^25^ -22.5 (c=0.1, DMSO); ^1^H NMR (300 MHz, DMSO-*d*_6_) δ 8.37 (ddd, *J* = 18.0, 15.2, 8.3 Hz, 4H, ArH), 7.93 (t, *J* = 5.7 Hz, 1H, NH), 7.88 – 7.72 (m, 3H, ArH, NH), 7.47 – 7.35 (m, 3H, ArH), 7.27 (d, *J* = 8.2 Hz, 1H, ArH), 4.68 (d, *J* = 10.3 Hz, 1H, H-1), 4.51 (s, 3H, 3 OH), 4.02 (t, *J* = 7.1 Hz, 2H, H-3, H-4), 3.85 (dd, *J* = 19.5, 9.8 Hz, 1H, H-2), 3.76 (d, *J* = 11.4 Hz, 1H, H-6b), 3.60 – 3.46 (m, 2H, H-5, H-6a), 3.40 – 3.21 (m, 2H, SCH_2_), 3.20 – 3.03 (m, 8H, 4 CH_2_), 1.86 – 1.73 (m, 6H, 3 CH_2_), 1.70 – 1.59 (m, 2H, CH_2_); ^13^C NMR (75 MHz, DMSO-*d*_6_) δ 169.02, 166.45, 163.73, 163.18, 156.85, 134.97, 132.40, 131.05, 130.70, 129.28, 128.26, 128.14, 127.43, 125.88, 125.53, 122.58, 115.06, 114.99, 83.74, 81.40, 75.21, 70.80, 61.48, 55.13, 54.08, 37.63, 37.07, 33.09, 28.10, 25.83, 23.97; HRMS (ESI) calcd for C_35_H_41_N_4_O_8_S (M+H^+^) 677.2645, found 677.2629.

*N*-[3-[6-(piperidin-1-yl)-1,3-dioxo-1*H*-benzo[*de*]isoquinolin-2(3*H*)-yl]propyl]-2-[(2-(4-fluorobenzamido)-β-D-glucopyranosyl)thio]acetamide (**16b**): yellow solid; (0.57 g, 83.2 %) yield; [α]_D_^25^ -34.7 (c=0.1, DMSO); ^1^H NMR (300 MHz, DMSO-*d*_6_) δ 8.48 – 8.28 (m, 4H, ArH), 7.99 – 7.86 (m, 3H, ArH, 2 NH), 7.83 – 7.70 (m, 1H, ArH), 7.30 – 7.17 (m, 3H, ArH), 5.15 (s, 2H, 2 OH), 4.68 (d, *J* = 10.3 Hz, 1H, H-1), 4.02 (t, *J* = 7.0 Hz, 2H, H-3, H-4), 3.92 – 3.71 (m, 2H, H-2 H-6b), 3.61 – 3.45 (m, 2H, H-6a, H-5), 3.40 – 3.24 (m, 2H, SCH_2_), 3.22 – 3.08 (m, 8H, 4 CH_2_), 1.90 – 1.72 (m, 6H, 3 CH_2_), 1.71 – 1.59 (m, 2H, CH_2_); ^13^C NMR (75 MHz, DMSO-*d*_6_) δ 169.00, 165.39, 163.71, 163.17, 156.83, 132.37, 131.43, 131.39, 130.67, 130.09, 129.98, 129.26, 125.85, 125.52, 122.55, 115.20, 115.04, 114.96, 114.91, 83.69, 81.40, 75.21, 70.74, 61.47, 55.22, 54.07, 37.62, 37.07, 33.06, 28.11, 25.83, 23.97; HRMS (ESI) calcd for C_35_H_40_FN_4_O_8_S (M+H^+^) 695.2551, found 695.2572.

*N*-[3-[6-(piperidin-1-yl)-1,3-dioxo-1*H*-benzo[*de*]isoquinolin-2(3*H*)-yl]propyl]-2-[[2-[[(phenylmethoxy)carbonyl]amino]-β-D-glucopyranosyl]thio]acetamide (**16c**): yellow solid; (0.62 g, 87.7 %) yield; [α]_D_^25^ -30.9 (c=0.1, DMSO); ^1^H NMR (300 MHz, DMSO-*d*_6_) δ 8.45 – 8.36 (m, 1H, ArH), 8.35 – 8.24 (m, 2H, ArH), 7.90 (t, *J* = 5.6 Hz, 1H, NH), 7.73 (dd, *J* = 8.3, 7.4 Hz, 1H, ArH), 7.42 – 7.15 (m, 7H, ArH, NH), 5.07 (s, 1H, OH), 5.04 – 4.92 (m, 2H, PhCH_2_), 4.56 (s, 1H, OH), 4.48 (d, *J* = 9.9 Hz, 1H, H-1), 4.01 (t, *J* = 7.1 Hz, 2H, H-3, H-4), 3.69 (d, *J* = 11.5 Hz, 1H, H-6b), 3.43 (d, *J* = 11.7 Hz, 1H, H-6a), 3.34 – 3.28 (m, 2H, H-2, H-5), 3.27 – 3.17 (m, 2H, SCH_2_), 3.17 – 3.03 (m, 8H, 4 CH_2_), 1.86 – 1.69 (m, 6H, 3 CH_2_), 1.68 – 1.53 (m, 2H, CH_2_); ^13^C NMR (75 MHz, DMSO-*d*_6_) δ 168.96, 163.73, 163.19, 156.82, 156.19, 137.24, 132.39, 130.69, 129.26, 128.34, 127.71, 127.65, 125.85, 125.51, 122.55, 115.03, 114.96, 83.94, 81.25, 75.35, 70.78, 65.32, 61.42, 56.83, 54.07, 37.65, 37.08, 33.08, 28.10, 25.83, 23.97; HRMS (ESI) calcd for C_36_H_43_N_4_O_9_S (M+H^+^) 707.2751, found 707.2745.

*N*-[3-[6-(piperidin-1-yl)-1,3-dioxo-1*H*-benzo[*de*]isoquinolin-2(3*H*)-yl]propyl]-2-[(2-propionamido-glucopyranosyl)thio]acetamide (**16d**): yellow solid; (0.51 g, 81.1 %) yield; [α]_D_^25^ -28.4 (c=0.1, DMSO); ^1^H NMR (300 MHz, DMSO-*d*_6_) δ 8.39 (d, *J* = 7.2 Hz, 1H, ArH), 8.31 (dd, *J* = 8.1, 3.6 Hz, 2H, ArH), 7.89 (t, *J* = 5.6 Hz, 1H, NH), 7.79 – 7.62 (m, 2H, ArH, NH), 7.22 (d, *J* = 8.2 Hz, 1H, ArH), 4.70 (br s, 3H, 3 OH), 4.46 (d, *J* = 10.3 Hz, 1H, H-1), 4.00 (t, *J* = 7.0 Hz, 2H, H-3, H-4), 3.68 (d, *J* = 11.4 Hz, 1H, H-6b), 3.61 – 3.48 (m, 1H, H-2), 3.43 (dd, *J* = 10.8, 4.5 Hz, 1H, H-6a), 3.34 – 3.16 (m, 3H, H-5, SCH_2_), 3.15 – 3.03 (m, 8H, 4 CH_2_), 2.03 (q, *J* = 7.5 Hz, 2H, CH_2_CH_3_), 1.82 – 1.69 (m, 6H, 3 CH_2_), 1.66 – 1.53 (m, 2H, CH_2_), 0.95 (t, *J* = 7.6 Hz, 3H, CH_3_); ^13^C NMR (75 MHz, DMSO-*d*_6_) δ 172.98, 169.06, 163.72, 163.17, 156.82, 132.37, 130.67, 129.25, 125.86, 125.51, 122.55, 115.04, 114.97, 83.84, 81.29, 75.42, 70.74, 61.45, 54.46, 54.07, 37.65, 37.06, 33.05, 28.89, 28.12, 25.82, 23.97, 9.84; HRMS (ESI) calcd for C_31_H_41_N_4_O_8_S (M+H^+^) 629.2645, found 629.2653.

*N*-[3-[6-(piperidin-1-yl)-1,3-dioxo-1*H*-benzo[*de*]isoquinolin-2(3*H*)-yl]propyl]-2-[(2-butyramido-β-D-glucopyranosyl)thio]acetamide (**16e**): yellow solid; (0.55 g, 85.5 %) yield; [α]_D_^25^ -26.1 (c=0.1, DMSO); ^1^H NMR (300 MHz, DMSO-*d*_6_) δ 8.47 – 8.34 (m, 1H, ArH), 8.34 – 8.23 (m, 2H, ArH), 7.88 (t, *J* = 5.7 Hz, 1H, NH), 7.80 – 7.63 (m, 2H, NH, ArH), 7.21 (d, *J* = 8.2 Hz, 1H, ArH), 4.55 (s, 3H, 3 OH), 4.46 (d, *J* = 10.3 Hz, 1H, H-1), 4.00 (t, *J* = 7.1 Hz, 2H, H-3, H-4), 3.69 (d, *J* = 11.3 Hz, 1H, H-6b), 3.56 (q, *J* = 9.8 Hz, 1H, H-2), 3.43 (dd, *J* = 11.6, 5.4 Hz, 1H, H-6a), 3.33 – 3.15 (m, 3H, H-5, SCH_2_), 3.15 – 3.03 (m, 8H, 4 CH_2_), 1.99 (t, *J* = 7.2 Hz, 2H, CH_2_), 1.84 – 1.70 (m, 6H, 3 CH_2_), 1.67 – 1.55 (m, 2H, CH_2_), 1.53 – 1.37 (m, 2H, CH_2_CH_3_), 0.80 (t, *J* = 7.4 Hz, 3H, CH_3_); ^13^C NMR (75 MHz, DMSO-*d*_6_) δ 172.16, 169.09, 163.70, 163.15, 156.81, 132.36, 130.66, 129.24, 125.84, 125.50, 122.53, 115.02, 114.95, 83.83, 81.29, 75.43, 70.79, 61.45, 54.41, 54.06, 37.85, 37.64, 37.07, 32.99, 28.12, 25.82, 23.96, 18.73, 13.75; HRMS (ESI) calcd for C_32_H_43_N_4_O_8_S (M+H^+^) 643.2802, found 643.2817.

*N*-[3-[6-(piperidin-1-yl)-1,3-dioxo-1*H*-benzo[*de*]isoquinolin-2(3*H*)-yl]propyl]-2-[(2-isobutyramido-β-D-glucopyranosyl)thio]acetamide (**16f**): yellow solid; (0.53 g, 82.4 %) yield; [α]_D_^25^ -20.3 (c=0.1, DMSO); ^1^H NMR (300 MHz, DMSO-*d*_6_) δ 8.37 (d, *J* = 7.3 Hz, 1H, ArH), 8.28 (dd, *J* = 7.9, 2.9 Hz, 2H, ArH), 7.87 (t, *J* = 5.6 Hz, 1H, NH), 7.76 – 7.68 (m, 1H, ArH), 7.62 (d, *J* = 9.4 Hz, 1H, NH), 7.19 (d, *J* = 8.2 Hz, 1H, ArH), 4.48 (d, *J* = 10.3 Hz, 1H, H-1), 4.36 (s, 3H, 3 OH), 3.99 (t, *J* = 7.0 Hz, 2H, H-3, H-4), 3.69 (d, *J* = 11.3 Hz, 1H, H-6b), 3.61 – 3.48 (m, 1H, H-2), 3.43 (dd, *J* = 11.5, 5.3 Hz, 1H, H-6a), 3.34 – 3.15 (m, 3H, H-5, SCH_2_), 3.14 – 3.04 (m, 8H, 4 CH_2_), 2.32 – 2.21 (m, 1H, CHCH_3_), 1.83 – 1.71 (m, 6H, 3 CH_2_), 1.67 – 1.54 (m, 2H, CH_2_), 0.95 (dd, *J* = 6.8, 3.6 Hz, 6H, 2 CH_3_); ^13^C NMR (75 MHz, DMSO-*d*_6_) δ 176.14, 169.13, 163.69, 163.14, 156.80, 132.35, 130.65, 129.22, 125.82, 125.48, 122.51, 115.00, 114.93, 83.88, 81.32, 75.37, 70.81, 61.47, 54.26, 54.05, 37.63, 37.07, 34.41, 32.97, 28.12, 25.82, 23.96, 19.74, 19.35; HRMS (ESI) calcd for C_32_H_43_N_4_O_8_S (M+H^+^) 643.2802, found 643.2786.

*N*-[3-[6-(piperidin-1-yl)-1,3-dioxo-1*H*-benzo[*de*]isoquinolin-2(3*H*)-yl]propyl]-2-[(2-trifluoroacetamido-β-D-glucopyranosyl)thio]acetamide (**16g**): yellow solid; (0.59 g, 88.3 %) yield; [α]_D_^25^ -17.5 (c=0.1, DMSO); ^1^H NMR (300 MHz, DMSO-*d*_6_) δ 9.28 (s, 1H, NH), 8.39 (d, *J* = 7.2 Hz, 1H, ArH), 8.31 (dd, *J* = 7.9, 4.4 Hz, 2H, ArH), 7.93 (t, *J* = 5.7 Hz, 1H, NH), 7.79 – 7.66 (m, 1H, ArH), 7.22 (d, *J* = 8.2 Hz, 1H, ArH), 5.30 (d, *J* = 5.7 Hz, 1H, OH), 5.16 (d, *J* = 5.1 Hz, 1H, OH), 4.69 – 4.52 (m, 2H, OH, H-1), 4.00 (t, *J* = 7.0 Hz, 2H, H-3, H-4), 3.69 (dd, *J* = 11.3, 6.2 Hz, 1H, H-6b), 3.64 – 3.51 (m, 1H, H-5), 3.50 – 3.38 (m, 2H, H-6a, H-2), 3.34 – 3.18 (m, 2H, SCH_2_), 3.18 – 3.04 (m, 8H, 4 CH_2_), 1.85 – 1.69 (m, 6H, 3 CH_2_), 1.68 – 1.54 (m, 2H, CH_2_); ^13^C NMR (75 MHz, DMSO-*d*_6_) δ 168.76, 163.72, 163.18, 156.82, 156.19, 132.37, 130.67, 129.26, 125.86, 125.51, 122.56, 117.99, 115.04, 114.97, 114.17, 82.41, 81.38, 74.45, 70.49, 61.26, 55.44, 54.07, 37.62, 37.08, 32.90, 28.07, 25.82, 23.96; HRMS (ESI) calcd for C_30_H_36_F_3_N_4_O_8_S (M+H^+^) 669.2206, found 669.2189.

*N*-[3-[6-(piperidin-1-yl)-1,3-dioxo-1*H*-benzo[*de*]isoquinolin-2(3*H*)-yl]propyl]-2-[[2-[[(methylamino)carbonyl]amino]-β-D-glucopyranosyl]thio]acetamide (**16h**): yellow solid; (0.55 g, 87.3 %) yield; [α]_D_^25^ -18.8 (c=0.1, DMSO); ^1^H NMR (300 MHz, DMSO-*d*_6_) δ 8.38 (d, *J* = 7.2 Hz, 1H, ArH), 8.29 (dd, *J* = 8.2, 2.6 Hz, 2H, ArH), 7.92 (t, *J* = 5.6 Hz, 1H, NHCH_2_), 7.72 (t, *J* = 7.9 Hz, 1H, ArH), 7.20 (d, *J* = 8.2 Hz, 1H, ArH), 5.85 (d, *J* = 8.8 Hz, 1H, NH, NHCH), 5.75 (q, *J* = 4.4 Hz, 1H, NHCH_3_), 5.13 – 4.91 (m, 2H, 2 OH), 4.56 (t, *J* = 5.6 Hz, 1H, OH), 4.48 (d, *J* = 10.1 Hz, 1H, H-1), 3.99 (t, *J* = 7.0 Hz, 2H, H-3, H-4), 3.68 (dd, *J* = 10.9, 5.7 Hz, 1H, H-6b), 3.49 – 3.36 (m, 2H, H-6a, H-2), 3.33 – 3.17 (m, 3H, H-5, SCH_2_), 3.17 – 3.01 (m, 8H, 4 CH_2_), 2.51 (d, *J* = 4.6 Hz, 3H, NCH_3_), 1.85 – 1.67 (m, 6H, 3 CH_2_), 1.66 – 1.52 (m, 2H, CH_2_); ^13^C NMR (75 MHz, DMSO-*d*_6_) δ 169.13, 163.70, 163.15, 158.63, 156.79, 132.36, 130.67, 129.23, 125.84, 125.49, 122.55, 115.04, 114.95, 84.36, 81.14, 76.25, 70.98, 61.49, 55.57, 54.06, 37.67, 37.08, 32.99, 28.13, 26.51, 25.82, 23.96; HRMS (ESI) calcd for C_30_H_40_N_5_O_8_S (M+H^+^) 630.2598, found 630.2583.

*N*-[3-[6-bromo-1,3-dioxo-1*H*-benzo[*de*]isoquinolin-2(3*H*)-yl]propyl]-2-[(2-benzamido-β-D-glucopyranosyl)thio]acetamide (**16j**): yellow solid; (0.53 g, 79.1 %) yield; [α]_D_^25^ -45.4 (c=0.1, DMSO); ^1^H NMR (300 MHz, DMSO-*d*_6_) δ 8.49 – 8.36 (m, 2H, ArH), 8.32 (d, *J* = 9.2 Hz, 1H, ArH), 8.19 (d, *J* = 7.9 Hz, 1H, ArH), 8.08 (d, *J* = 7.9 Hz, 1H, ArH), 7.99 – 7.85 (m, 2H, 2 NH), 7.85 – 7.78 (m, 2H, ArH), 7.44 – 7.34 (m, 3H, ArH), 4.71 (br s, 3H, 3 OH), 4.67 (d, *J* = 10.3 Hz, 1H, H-1), 3.98 (t, *J* = 7.1 Hz, 2H, H-3, H-4), 3.84 (dd, *J* = 19.5, 9.8 Hz, 1H, H-2), 3.74 (d, *J* = 11.4 Hz, 1H, H-6b), 3.61 – 3.44 (m, 2H, H-6a , H-5), 3.39 – 3.21 (m, 2H, SCH_2_), 3.21 – 3.17 (m, 2H, CH_2_), 3.16 – 3.05 (m, 2H, CH_2_), 1.79 – 1.71 (m, 2H, CH_2_); ^13^C NMR (75 MHz, DMSO-*d*_6_) δ 169.11, 166.48, 162.94, 162.89, 134.94, 132.61, 131.60, 131.34, 131.07, 130.98, 129.78, 129.21, 128.76, 128.26, 128.15, 127.44, 122.71, 121.94, 83.80, 81.37, 75.19, 70.80, 61.49, 55.14, 38.03, 37.08, 33.16, 27.90; HRMS (ESI) calcd for C_30_H_31_BrN_3_O_8_S (M+H^+^) 672.1015, found 672.1028.

*N*-[3-[6-bromo-1,3-dioxo-1*H*-benzo[*de*]isoquinolin-2(3*H*)-yl]propyl]-2-[(2-(4-fluorobenzamido)-β-D-glucopyranosyl)thio]acetamide (**16k**): yellow solid; (0.60 g, 86.9 %) yield; [α]_D_^25^ -40.5 (c=0.1, DMSO); ^1^H NMR (300 MHz, DMSO-*d*_6_) δ 8.49 – 8.32 (m, 3H, ArH), 8.20 (d, *J* = 7.9 Hz, 1H, ArH), 8.09 (d, *J* = 7.9 Hz, 1H, ArH), 7.96 – 7.84 (m, 4H, 2 NH, ArH), 7.21 (t, *J* = 8.8 Hz, 2H, ArH), 4.66 (d, *J* = 10.2 Hz, 1H, H-1), 4.56 (br s, 3H, 3 OH), 3.98 (t, *J* = 7.0 Hz, 2H, H-3, H-4), 3.88 – 3.68 (m, 2H, H-2, H-6b), 3.58 – 3.44 (m, 2H, H-5, H-6a), 3.37 – 3.20 (m, 2H, SCH_2_), 3.18 – 3.04 (m, 4H, 2 CH_2_), 1.77 – 1.65 (m, 2H, CH_2_); ^13^C NMR (75 MHz, DMSO-*d*_6_) δ 169.10, 165.41, 162.95, 162.90, 132.62, 131.60, 131.35, 130.98, 130.10, 129.99, 129.80, 129.21, 128.77, 128.28, 122.72, 121.94, 115.19, 114.91, 83.76, 81.37, 75.17, 70.75, 61.47, 55.22, 38.02, 37.07, 33.13, 27.90; HRMS (ESI) calcd for C_30_H_30_BrFN_3_O_8_S (M+H^+^) 690.0921, found 690.0904.

*N*-[3-[6-bromo-1,3-dioxo-1*H*-benzo[*de*]isoquinolin-2(3*H*)-yl]propyl]-2-[(2-propionamido-β-D-glucopyranosyl)thio]acetamide (**16l**): yellow solid; (0.52 g, 83.3 %) yield; [α]_D_^25^ -37.6 (c=0.1, DMSO); ^1^H NMR (300 MHz, DMSO-*d*_6_) δ 8.50 – 8.36 (m, 2H, ArH), 8.20 (d, *J* = 7.9 Hz, 1H, ArH), 8.09 (d, *J* = 7.9 Hz, 1H, ArH), 7.97 – 7.85 (m, 2H, 2 NH), 7.66 (d, *J* = 9.3 Hz, 1H, ArH), 4.47 (d, *J* = 10.3 Hz, 1H, H-1), 4.23 (br s, 3H, 3 OH), 4.00 (t, *J* = 7.1 Hz, 2H, H-3, H-4), 3.69 (d, *J* = 11.1 Hz, 1H, H-6b), 3.56 (dd, *J* = 19.5, 9.8 Hz, 1H, H-2), 3.44 (dd, *J* = 11.7, 5.4 Hz, 1H, H-6a), 3.36 – 3.18 (m, 3H, H-5, SCH_2_), 3.16 – 3.06 (m, 4H, 2 CH_2_), 2.05 (q, *J* = 7.5 Hz, 2H, CH_2_CH_3_), 1.82 – 1.74 (m, 2H, CH_2_), 0.96 (t, *J* = 7.6 Hz, 3H, CH_3_); ^13^C NMR (75 MHz, DMSO-*d*_6_) δ 173.02, 169.13, 162.96, 162.91, 132.62, 131.61, 131.37, 130.99, 129.80, 129.20, 128.79, 128.28, 122.75, 121.97, 83.88, 81.27, 75.42, 70.76, 61.47, 54.49, 38.06, 37.08, 33.11, 28.90, 27.93, 9.85; HRMS (ESI) calcd for C_26_H_31_BrN_3_O_8_S (M+H^+^) 624.1015, found 624.1003.

*N*-[3-[6-bromo-1,3-dioxo-1*H*-benzo[*de*]isoquinolin-2(3*H*)-yl]propyl]-2-[(2-butyramido-β-D-glucopyranosyl)thio]acetamide (**16m**): yellow solid; (0.55 g, 85.9 %) yield; [α]_D_^25^ -33.7 (c=0.1, DMSO); ^1^H NMR (300 MHz, DMSO-*d*_6_) δ 8.44 (dd, *J* = 16.1, 7.9 Hz, 2H, ArH), 8.23 (d, *J* = 7.9 Hz, 1H, ArH), 8.11 (d, *J* = 7.9 Hz, 1H, ArH), 7.97 – 7.86 (m, 2H, 2 NH), 7.70 (d, *J* = 9.3 Hz, 1H, ArH), 4.48 (d, *J* = 10.3 Hz, 1H, H-1), 4.36 (br s, 3H, 3 OH), 4.01 (t, *J* = 7.0 Hz, 2H, H-3, H-4), 3.69 (d, *J* = 11.5 Hz, 1H, H-6b), 3.57 (dd, *J* = 19.5, 9.7 Hz, 1H, H-2), 3.44 (dd, *J* = 12.0, 5.3 Hz, 1H, H-6a), 3.35 – 3.17 (m, 3H, H-5, SCH_2_), 3.16 – 3.06 (m, 4H, 2 CH_2_), 2.01 (t, *J* = 7.2 Hz, 2H, CH_2_), 1.86 – 1.70 (m, 2H, CH_2_), 1.56 – 1.40 (m, 2H, CH_2_), 0.82 (t, *J* = 7.4 Hz, 3H, CH_3_); ^13^C NMR (75 MHz, DMSO-*d*_6_) δ 172.18, 169.15, 162.99, 162.93, 132.65, 131.63, 131.39, 131.02, 129.83, 129.21, 128.82, 128.32, 122.78, 122.01, 83.86, 81.27, 75.42, 70.81, 61.46, 54.44, 38.06, 37.86, 37.08, 33.05, 27.93, 18.73, 13.75; HRMS (ESI) calcd for C_27_H_33_BrN_3_O_8_S (M+H^+^) 638.1172, found 638.1159.

*N*-[3-[6-bromo-1,3-dioxo-1*H*-benzo[*de*]isoquinolin-2(3*H*)-yl]propyl]-2-[(2-isobutyramido-β-D-glucopyranosyl)thio]acetamide (**16n**): yellow solid; (0.53 g, 82.8 %) yield; [α]_D_^25^ -26.5 (c=0.1, DMSO); ^1^H NMR (300 MHz, DMSO-*d*_6_) δ 8.51 – 8.40 (m, 2H, ArH), 8.23 (d, *J* = 7.9 Hz, 1H, ArH), 8.12 (d, *J* = 7.9 Hz, 1H, ArH), 7.96 – 7.87 (m, 2H, 2 NH), 7.63 (d, *J* = 9.2 Hz, 1H, ArH), 4.49 (d, *J* = 10.3 Hz, 1H, H-1), 4.37 (br s, 3H, 3 OH), 4.01 (t, *J* = 7.1 Hz, 2H, H-3, H-4), 3.69 (d, *J* = 11.2 Hz, 1H, H-6b), 3.55 (dd, *J* = 19.6, 9.8 Hz, 1H, H-2), 3.44 (dd, *J* = 11.5, 5.3 Hz, 1H, H-6a), 3.34 – 3.16 (m, 3H, H-5, SCH_2_), 3.15 – 3.05 (m, 4H, 2 CH_2_), 2.32 – 2.25 (m, 1H, CHCH_3_), 1.83 – 1.72 (m, 2H, CH_2_), 1.00 – 0.91 (m, 6H, 2 CH_3_); ^13^C NMR (75 MHz, DMSO-*d*_6_) δ 176.15, 169.18, 162.99, 162.94, 132.66, 131.64, 131.40, 131.02, 129.84, 129.21, 128.83, 128.34, 122.80, 122.02, 83.92, 81.31, 75.35, 70.82, 61.47, 54.28, 38.05, 37.08, 34.41, 33.02, 27.94, 19.74, 19.36; HRMS (ESI) calcd for C_27_H_33_BrN_3_O_8_S (M+H^+^) 638.1172, found 638.1155.

*N*-[3-[6-bromo-1,3-dioxo-1*H*-benzo[*de*]isoquinolin-2(3*H*)-yl]propyl]-2-[(2-trifluoroacetamido-β-D-glucopyranosyl)thio]acetamide (**16o**): yellow solid; (0.59 g, 88.9 %) yield; [α]_D_^25^ -42.3 (c=0.1, DMSO); ^1^H NMR (300 MHz, DMSO-*d*_6_) δ 8.56 – 8.44 (m, 2H, ArH), 8.27 (d, *J* = 7.9 Hz, 1H, ArH), 8.15 (d, *J* = 7.9 Hz, 1H, ArH), 7.99 – 7.90 (m, 2H, ArH, NH), 4.63 (d, *J* = 10.2 Hz, 1H, H-1), 4.02 (t, *J* = 7.1 Hz, 2H, H-3, H-4), 3.69 (d, *J* = 11.6 Hz, 1H, H-6b), 3.63 – 3.52 (m, 1H, H-2), 3.50 – 3.39 (m, 2H, H-5, H-6a), 3.27 (dd, *J* = 43.0, 14.1 Hz, 2H, SCH_2_), 3.16 – 3.05 (m, 4H, 2 CH_2_), 1.85 – 1.71 (m, 2H, CH_2_); ^13^C NMR (75 MHz, DMSO-*d*_6_) δ 168.82, 163.04, 162.99, 156.67, 156.20, 132.69, 131.67, 131.43, 131.05, 129.89, 129.21, 128.87, 128.41, 122.87, 122.10, 118.00, 114.17, 82.48, 81.38, 74.44, 70.51, 61.28, 55.45, 38.03, 37.09, 32.95, 27.88; HRMS (ESI) calcd for C_25_H_26_BrF_3_N_3_O_8_S (M+H^+^) 664.0576, found 664.0558.

**1.3. New method for the synthesis of thioglycosyl−naphthalimides 16h.**

**Scheme S3.** Synthesis of thioglycosyl−naphthalimides **16h**. (i) CH_3_NH_2_, MeOH.

A solution of **15i** (1.0 mmol) was suspended in anhydrous MeOH (20 mL), and saturated solution of CH_3_NH_2_ in MeOH (10 mL) was then added. The reaction was stirred for 48 h at room temperature, until TLC (EtOAc/MeOH/H_2_O, 8:1:1 v/v/v) indicated that the reaction was complete. The mixture was concentrated *in vacuo* and recrystallized from MeOH/ether to obtain **16h**.

*N*-[3-[6-(piperidin-1-yl)-1,3-dioxo-1*H*-benzo[*de*]isoquinolin-2(3*H*)-yl]propyl]-2-[[2-[[(methylamino)carbonyl]amino]-β-D-glucopyranosyl]thio]acetamide (**16h**): yellow solid; (0.49 g, 77.8 %) yield; [α]_D_^25^ -18.8 (c=0.1, DMSO); ^1^H NMR (300 MHz, DMSO-*d*_6_) δ 8.38 (d, *J* = 7.2 Hz, 1H, ArH), 8.29 (dd, *J* = 8.2, 2.6 Hz, 2H, ArH), 7.92 (t, *J* = 5.6 Hz, 1H, NHCH_2_), 7.72 (t, *J* = 7.9 Hz, 1H, ArH), 7.20 (d, *J* = 8.2 Hz, 1H, ArH), 5.85 (d, *J* = 8.8 Hz, 1H, NH, NHCH), 5.75 (q, *J* = 4.4 Hz, 1H, NHCH_3_), 5.13 – 4.91 (m, 2H, 2 OH), 4.56 (t, *J* = 5.6 Hz, 1H, OH), 4.48 (d, *J* = 10.1 Hz, 1H, H-1), 3.99 (t, *J* = 7.0 Hz, 2H, H-3, H-4), 3.68 (dd, *J* = 10.9, 5.7 Hz, 1H, H-6b), 3.49 – 3.36 (m, 2H, H-6a, H-2), 3.33 – 3.17 (m, 3H, H-5, SCH_2_), 3.17 – 3.01 (m, 8H, 4 CH_2_), 2.51 (d, *J* = 4.6 Hz, 3H, NCH_3_), 1.85 – 1.67 (m, 6H, 3 CH_2_), 1.66 – 1.52 (m, 2H, CH_2_); ^13^C NMR (75 MHz, DMSO-*d*_6_) δ 169.13, 163.70, 163.15, 158.63, 156.79, 132.36, 130.67, 129.23, 125.84, 125.49, 122.55, 115.04, 114.95, 84.36, 81.14, 76.25, 70.98, 61.49, 55.57, 54.06, 37.67, 37.08, 32.99, 28.13, 26.51, 25.82, 23.96; HRMS (ESI) calcd for C_30_H_40_N_5_O_8_S (M+H^+^) 630.2598, found 630.2583.

**1.4. Synthesis of ureido glycosides 17a-17d.**

**17a**: R_1_=piperidyl, R_3_=C_2_H_5_; **17b**: R_1_=piperidyl, R_3_=n-Pr; **17c**: R_1_=Br, R_3_=CH_3_; **17d**: R_1_=Br, R_3_=C_2_H_5_

**Scheme S4.** Synthesis of ureido glycosides **17a-17d**. (i) R_3_NH_2_, MeOH.

A solution of **15i** (1.0 mmol) was suspended in anhydrous MeOH (20 mL), and substituted amine (2 mL) was then added. The reaction was stirred for 48 h at room temperature, until TLC (EtOAc/MeOH/H_2_O, 8:1:1 v/v/v) indicated that the reaction was complete. The mixture was concentrated *in vacuo* and recrystallized from MeOH/ether to obtain **17a-17d**.

*N*-[3-[6-(piperidin-1-yl)-1,3-dioxo-1*H*-benzo[*de*]isoquinolin-2(3*H*)-yl]propyl]-2-[[2-[[(ethylamino)carbonyl]amino]-β-D-glucopyranosyl]thio]acetamide (**17a**): yellow solid; (0.48 g, 74.5 %) yield; [α]_D_^25^ -25.3 (c=0.1, DMSO); ^1^H NMR (300 MHz, DMSO-*d*_6_) δ 8.45 (d, *J* = 7.2 Hz, 1H, ArH), 8.41 – 8.33 (m, 2H, ArH), 7.91 (t, *J* = 5.6 Hz, 1H, NH), 7.84 – 7.74 (m, 1H, ArH), 7.28 (d, *J* = 8.2 Hz, 1H, ArH), 5.93 – 5.73 (m, 2H, 2NH), 4.47 (d, *J* = 10.1 Hz, 1H, H-1), 4.33 (s, 3H, 3 OH), 4.03 (t, *J* = 7.1 Hz, 2H, H-3, H-4), 3.69 (d, *J* = 10.6 Hz, 1H, H-6b), 3.49 – 3.37 (m, 2H, H-6a, H-2), 3.37 – 3.21 (m, 3H, H-5, SCH_2_), 3.20 – 3.03 (m, 8H, 4 CH_2_), 3.01 – 2.94 (m, 2H, CH_2_), 1.86 – 1.71 (m, 6H, 3 CH_2_), 1.69 – 1.56 (m, 2H, CH_2_), 0.95 (t, *J* = 7.2 Hz, 3H, CH_3_); ^13^C NMR (75 MHz, DMSO-*d*_6_) δ 169.15, 163.73, 163.18, 158.06, 156.83, 132.39, 130.70, 129.28, 125.87, 125.54, 122.59, 115.08, 114.99, 84.46, 81.17, 76.27, 71.01, 61.50, 55.49, 54.08, 37.67, 37.08, 34.27, 33.01, 28.14, 25.83, 23.97, 15.70; HRMS (ESI) calcd for C_31_H_42_N_5_O_8_S (M+H^+^) 644.2754, found 644.2767.

*N*-[3-[6-(piperidin-1-yl)-1,3-dioxo-1*H*-benzo[*de*]isoquinolin-2(3*H*)-yl]propyl]-2-[[2-[[(propylamino)carbonyl]amino]-β-D-glucopyranosyl]thio]acetamide (**17b**): yellow solid; (0.49 g, 74.5 %) yield; [α]_D_^25^ -23.9 (c=0.1, DMSO); ^1^H NMR (300 MHz, DMSO-*d*_6_) δ 8.49 – 8.32 (m, 3H, ArH), 7.91 (t, *J* = 5.6 Hz, 1H, NH), 7.77 (dd, *J* = 8.3, 7.4 Hz, 1H, ArH), 7.27 (d, *J* = 8.2 Hz, 1H, ArH), 5.85 (t, *J* = 5.6 Hz, 1H, NH), 5.78 (d, *J* = 8.8 Hz, 1H, NH), 4.99 (s, 2H, 2 OH), 4.48 (d, *J* = 10.1 Hz, 1H, H-1), 4.03 (t, *J* = 7.1 Hz, 2H, H-3, H-4), 3.69 (d, *J* = 10.7 Hz, 1H, H-6b), 3.53 – 3.25 (m, 5H, H-6a, H-2, H-5, SCH_2_), 3.19 – 3.02 (m, 8H, 4 CH_2_), 2.96 – 2.82 (m, 2H, CH_2_), 1.85 – 1.72 (m, 6H, 3 CH_2_), 1.70 – 1.56 (m, 2H, CH_2_), 1.38 – 1.28 (m, 2H, CH_2_), 0.78 (t, *J* = 7.4 Hz, 3H, CH_3_); ^13^C NMR (75 MHz, DMSO-*d*_6_) δ 169.16, 163.69, 163.14, 158.15, 156.79, 132.35, 130.66, 130.62, 129.23, 125.82, 125.49, 122.53, 115.02, 114.93, 84.44, 81.15, 76.31, 71.02, 61.51, 55.50, 54.06, 41.33, 37.66, 37.10, 32.97, 28.13, 25.82, 23.96, 23.29, 11.43; HRMS (ESI) calcd for C_32_H_44_N_5_O_8_S (M+H^+^) 658.2911, found 658.2902.

*N*-[3-[6-bromo-1,3-dioxo-1*H*-benzo[*de*]isoquinolin-2(3*H*)-yl]propyl]-2-[[2-[[(methylamino)carbonyl]amino]-β-D-glucopyranosyl]thio]acetamide (**17c**): yellow solid; (0.45 g, 72.0 %) yield; [α]_D_^25^ -32.5 (c=0.1, DMSO); ^1^H NMR (300 MHz, DMSO-*d*_6_) δ 8.43 (dd, *J* = 16.7, 7.9 Hz, 2H, ArH), 8.21 (d, *J* = 7.9 Hz, 1H, ArH), 8.09 (d, *J* = 7.9 Hz, 1H, ArH), 7.96 (t, *J* = 5.7 Hz, 1H, NH), 7.93 – 7.85 (m, 1H, ArH), 5.96 (d, *J* = 8.7 Hz, 1H, NH), 5.82 (d, *J* = 4.5 Hz, 1H, NH), 4.57 (br s, 3H, 3 OH), 4.48 (d, *J* = 10.1 Hz, 1H, H-1), 4.04 – 3.96 (m, 2H, H-3, H-4), 3.69 (d, *J* = 10.9 Hz, 1H, H-6b), 3.48 – 3.34 (m, 2H, H-2, H-6a), 3.34 – 3.18 (m, 3H, H-5, SCH_2_), 3.16 – 3.05 (m, 4H, 2 CH_2_), 2.56 – 2.52 (m, 3H, NCH_3_), 1.81 – 1.75 (m, 2H, CH_2_); ^13^C NMR (75 MHz, DMSO-*d*_6_) δ 169.22, 162.98, 162.93, 158.68, 132.62, 131.62, 131.37, 131.00, 129.81, 129.19, 128.79, 128.30, 122.77, 121.99, 84.44, 81.12, 76.23, 70.98, 61.50, 55.58, 38.08, 37.09, 33.06, 27.93, 26.51; HRMS (ESI) calcd for C_25_H_30_BrN_4_O_8_S (M+H^+^) 625.0968, found 625.0949.

*N*-[3-[6-bromo-1,3-dioxo-1*H*-benzo[*de*]isoquinolin-2(3*H*)-yl]propyl]-2-[[2-[[(ethylamino)carbonyl]amino]-β-D-glucopyranosyl]thio]acetamide (**17d**): yellow solid; (0.45 g, 70.4 %) yield; [α]_D_^25^ -16.2 (c=0.1, DMSO); ^1^H NMR (300 MHz, DMSO-*d*_6_) δ 8.45 (d, *J* = 7.2 Hz, 1H, ArH), 8.39 (d, *J* = 8.4 Hz, 1H, ArH), 8.20 (d, *J* = 7.9 Hz, 1H, ArH), 8.09 (d, *J* = 7.9 Hz, 1H, ArH), 8.01 – 7.83 (m, 2H, ArH, NH), 5.90 – 5.81 (m, 2H, 2 NH), 4.48 (d, *J* = 10.1 Hz, 1H, H-1), 4.42 (s, 3H, 3 OH), 4.06 – 3.95 (m, 2H, H-3, H-4), 3.69 (d, *J* = 10.8 Hz, 1H, H-6b), 3.50 – 3.34 (m, 3H, H-2, H-6a, H-5), 3.30 – 3.20 (m, 2H, SCH_2_), 3.19 – 3.09 (m, 4H, 2 CH_2_), 3.03 – 2.93 (m, 2H, CH_2_), 1.87 – 1.73 (m, 2H, CH_2_), 0.95 (t, *J* = 7.2 Hz, 3H, CH_3_); ^13^C NMR (75 MHz, DMSO-*d*_6_) δ 169.23, 162.96, 162.91, 158.05, 132.62, 131.62, 131.36, 131.00, 129.80, 129.19, 128.79, 128.28, 122.75, 121.97, 84.49, 81.13, 76.26, 71.02, 61.51, 55.49, 38.07, 37.10, 34.29, 33.05, 27.93, 15.69; HRMS (ESI) calcd for C_26_H_32_BrN_4_O_8_S (M+H^+^) 639.1124, found 639.1112.

**2. The supplementary** **MD simulations studies.**

In order to further investigate the binding modes of **10a**, **10d** and **16j** with hOGA, we extended the MD simulations of the three systems by 20 ns. As shown in **Figure S1a**, 45 ns of MD simulations were implemented, the dynamic convergences of these three systems were all achieved after 22 ns of simulations. Then, the conformations of **10a**, **10d** and **16j** in complex with hOGA at 45 ns of MD simulations were extracted and superposed with the conformations at 25 ns, respectively (**Figures S1b-1d**). These results show that the conformations of **10a**, **10d** and **16j** exhibit small change in the MD simulations of 25 ns and 45 ns.


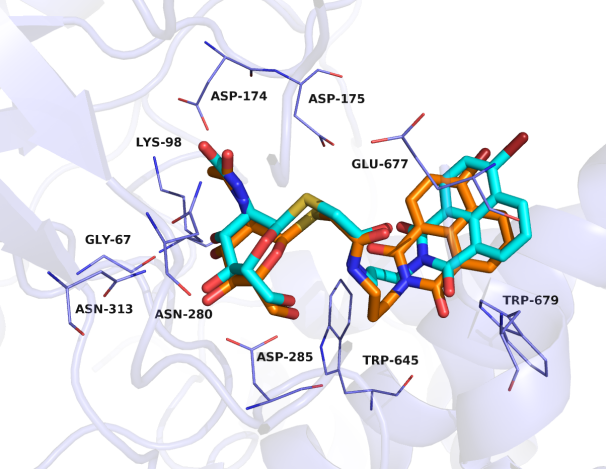


**(b)**

**(a)**


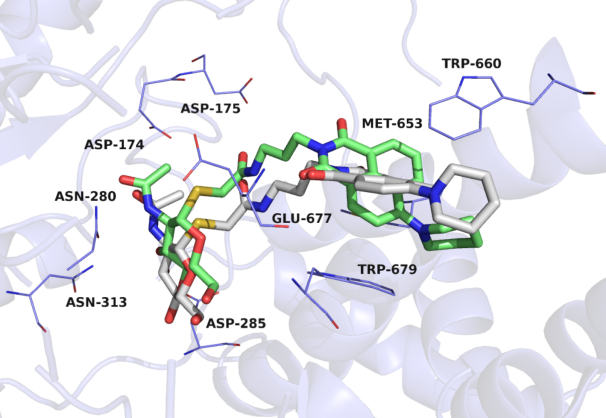

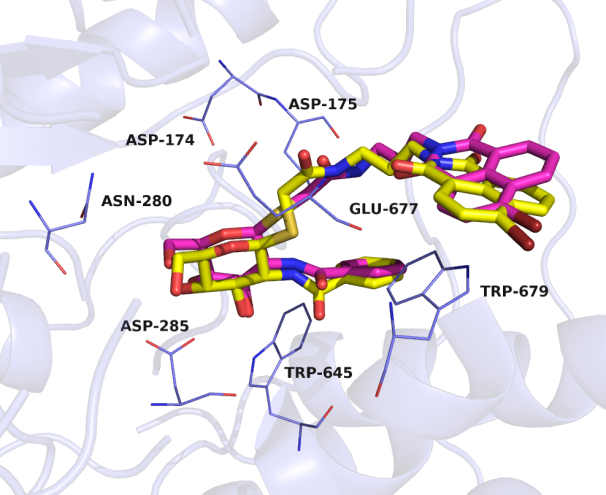


**(d)**

**(c)**

**Figure S1.** (a) RMSD changes of hOGA in complex with **10a**, **10d**, and **16j** during 45 ns. (b) Superimposition of conformations of **10a** in hOGA active pocket at 25 ns (cyan colored carbon atoms) and 45 ns (orange colored carbon atoms) MD simulations. (c) Superimposition of conformations of **10d** in hOGA active pocket at 25 ns (green colored carbon atoms) and 45 ns (white colored carbon atoms) MD simulations. (d) Superimposition of conformations of **16j** in hOGA active pocket at 25 ns (yellow colored carbon atoms) and 45 ns (pink colored carbon atoms) MD simulations.

**References**

1. Shen, S.; Dong, L.; Chen, W.; Zeng, X.; Lu, H.; Yang, Q.; Zhang, J., Modification of the thioglycosyl-naphthalimides as potent and selective human O-GlcNAcase Inhibitors. *ACS Med. Chem. Lett.* **2018,** *9* (12), 1241-1246.

2. Shen, S.; Chen, W.; Dong, L.; Yang, Q.; Lu, H.; Zhang, J., Design and synthesis of naphthalimide group-bearing thioglycosides as novel β-N-acetylhexosaminidases inhibitors. *J. Enzyme Inhib. Med. Chem.* **2018,** *33* (1), 445-452.

3. Kong, H.; Chen, W.; Lu, H.; Yang, Q.; Dong, Y.; Wang, D.; Zhang, J., Synthesis of NAG-thiazoline-derived inhibitors for β-N-acetyl-D-hexosaminidases. *Carbohydr. Res.* **2015,** *413*, 135-144.

4. Macauley, M. S.; Whitworth, G. E.; Debowski, A. W.; Chin, D.; Vocadlo, D. J., O-GlcNAcase Uses Substrate-assisted Catalysis: kinetic analysis and development of highly selective mechanism-inspired inhibitors. *J. Biol. Chem.* **2005,** *280* (27), 25313-25322.

5. Greig, I. R.; Macauley, M. S.; Williams, I. H.; Vocadlo, D. J., Probing synergy between two catalytic strategies in the glycoside hydrolase O-GlcNAcase using multiple linear free energy relationships. *J. Am. Chem. Soc.* **2009,** *131* (37), 13415-13422.

**^1^H NMR and ^13^C NMR spectrum**

**^1^H NMR spectrum of 9a** **(300 MHz, DMSO-*d_6_*)**


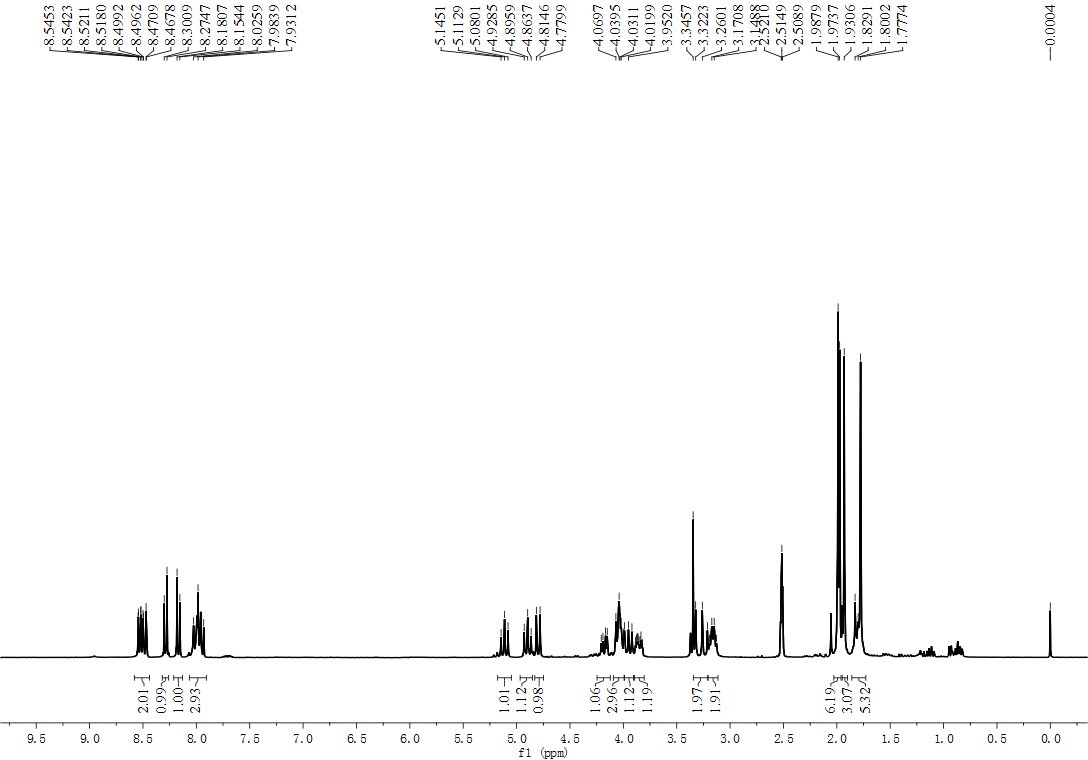


**^13^C NMR spectrum of 9a (300 MHz, DMSO-*d_6_*)**


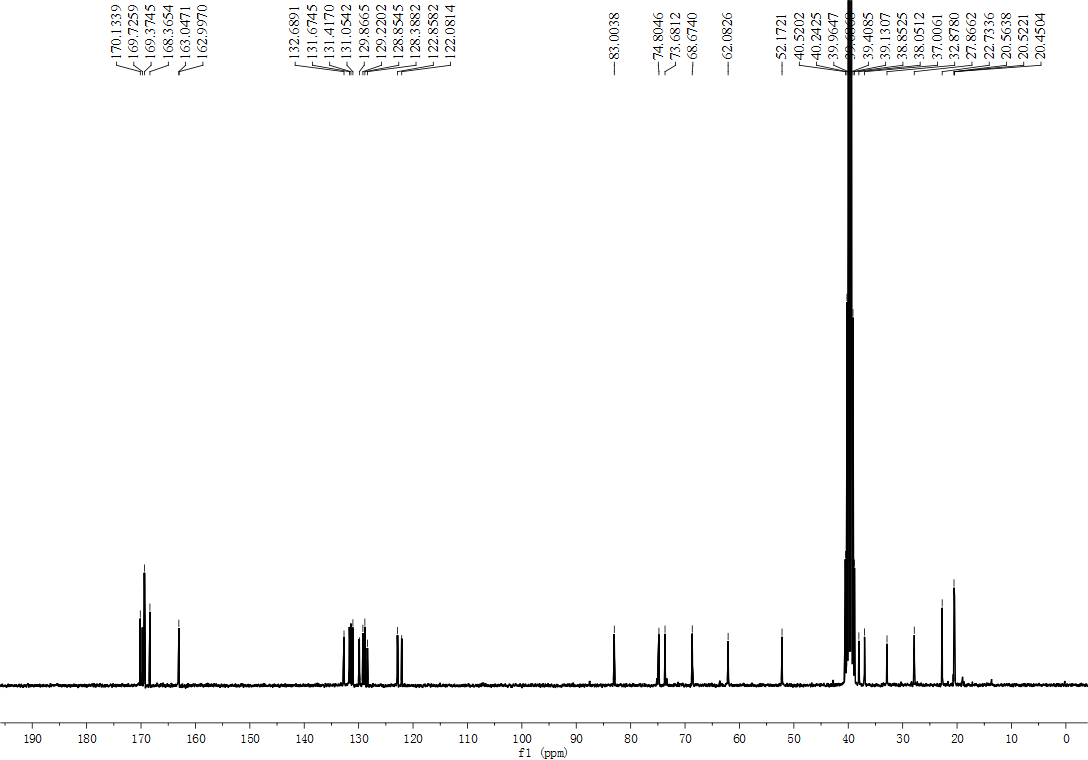


**^1^H NMR spectrum of 9b (300 MHz, DMSO-*d_6_*)**


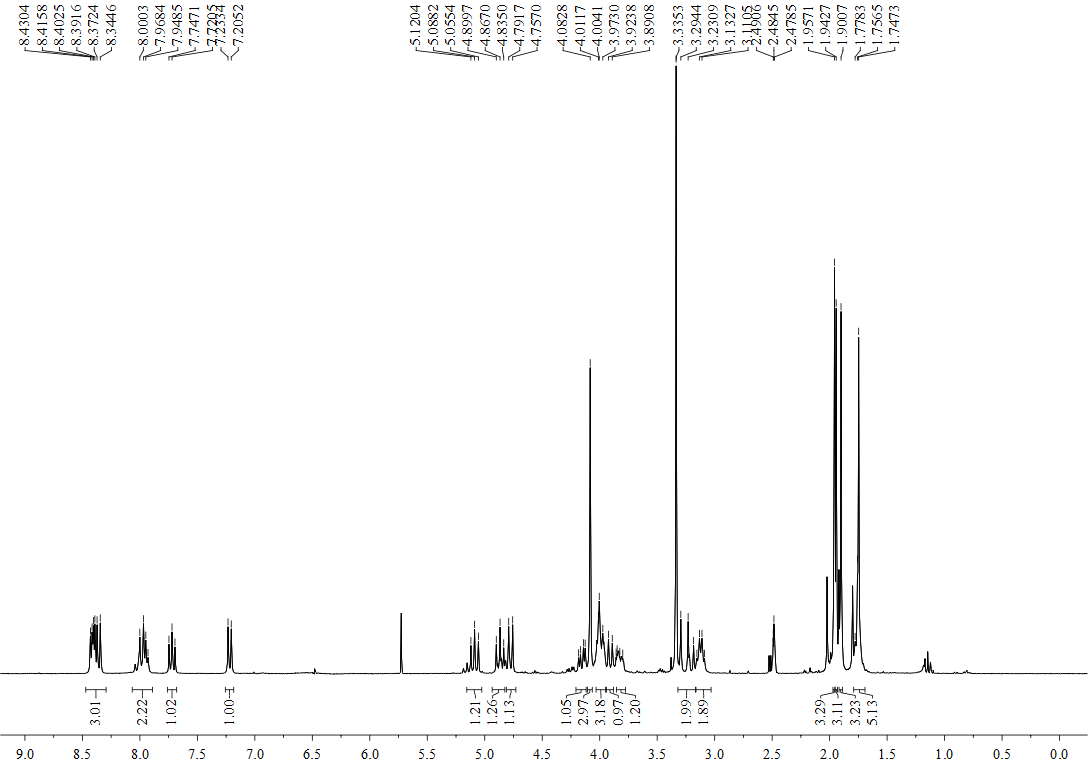


**^13^C NMR spectrum of 9b (300 MHz, DMSO-*d_6_*)**


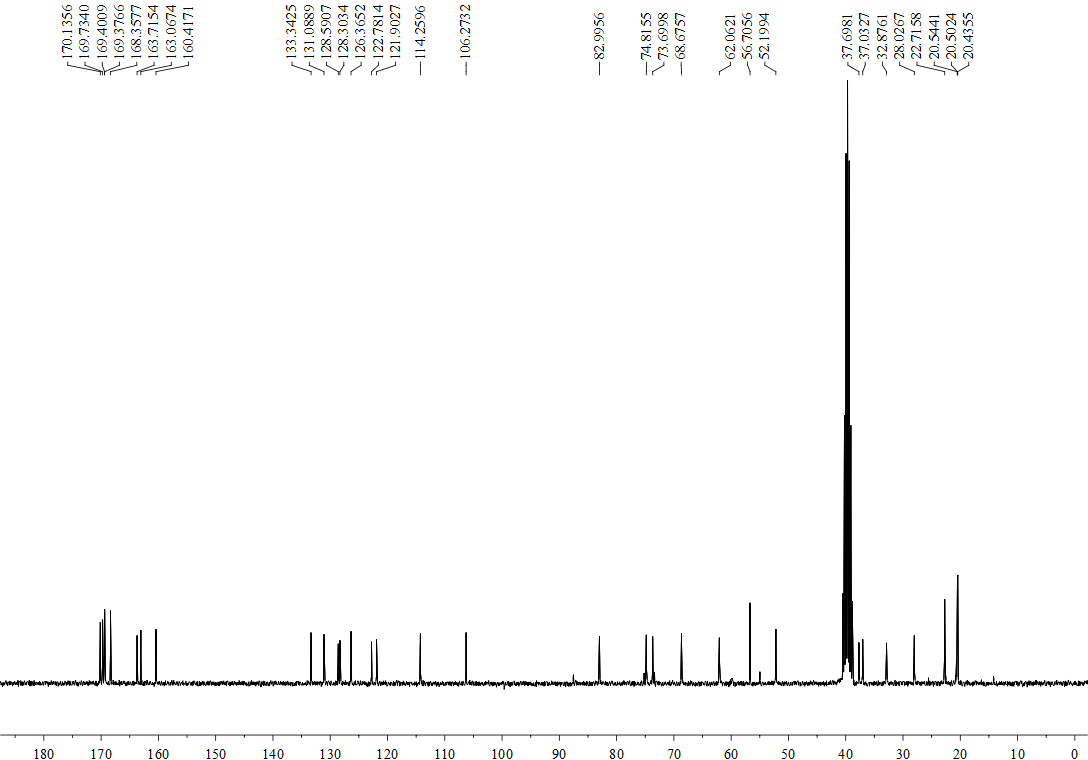


**^1^H NMR spectrum of 9c (300 MHz, DMSO-*d_6_*)**


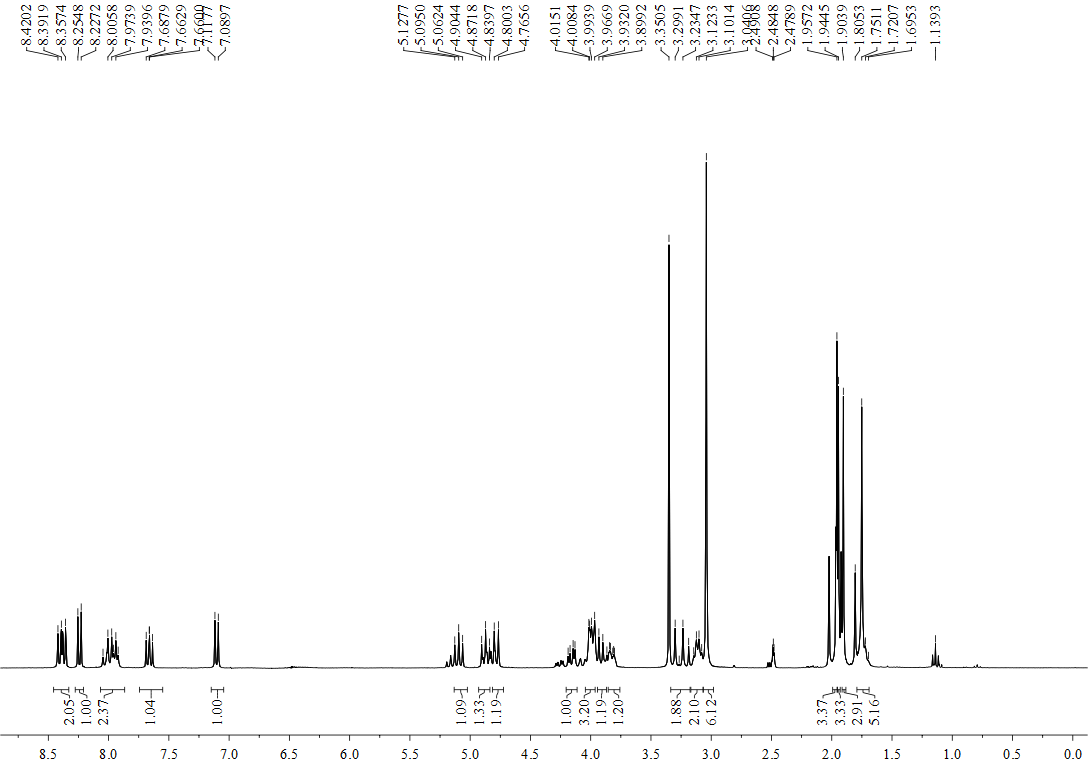


**^13^C NMR spectrum of 9c (300 MHz, DMSO-*d_6_*)**


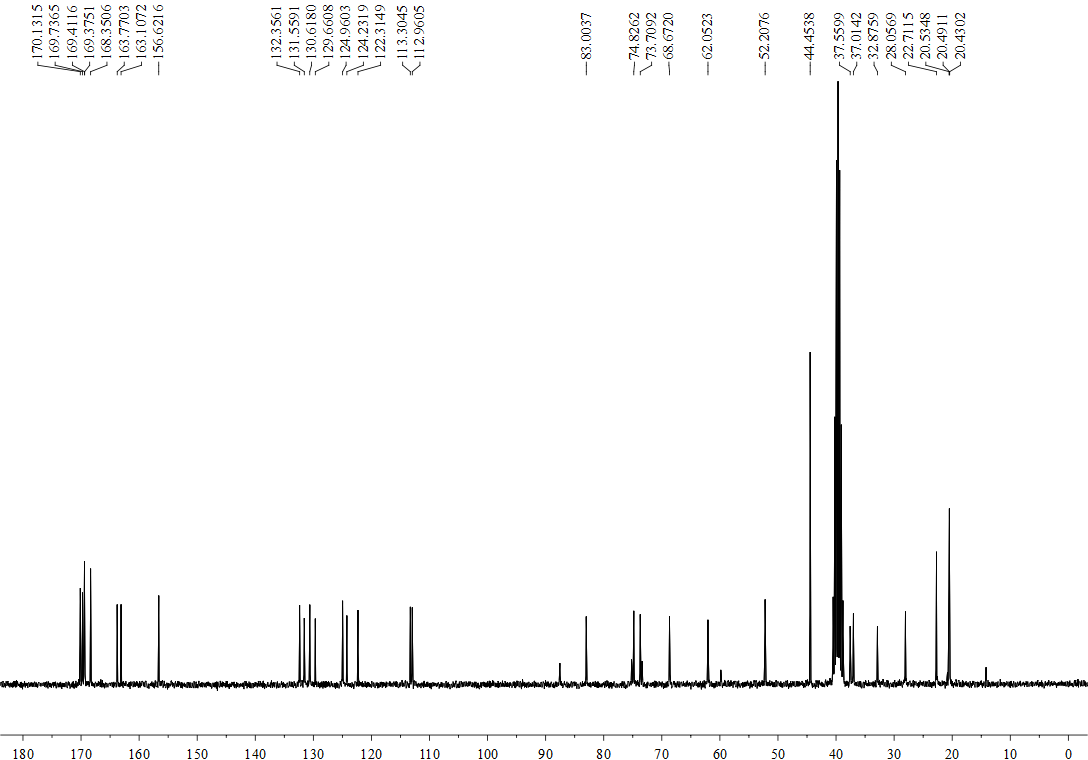


**^1^H NMR spectrum of 9d (300 MHz, DMSO-*d_6_*)**


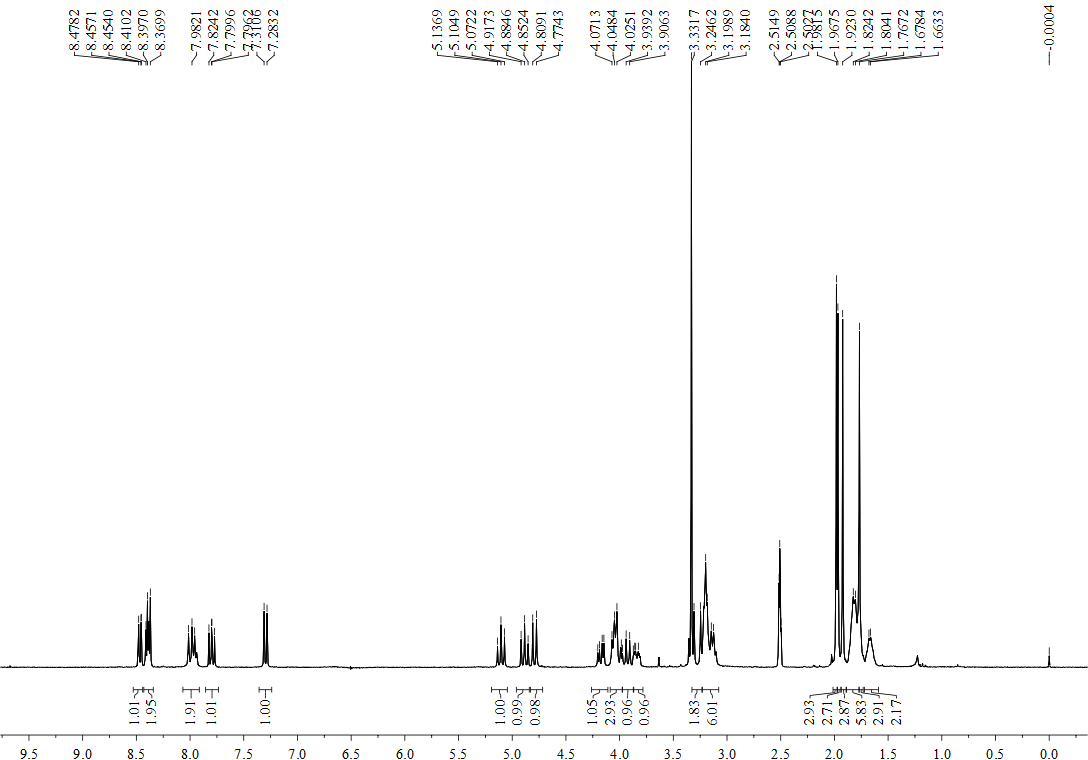


**^13^C NMR spectrum of 9d (300 MHz, DMSO-*d_6_*)**


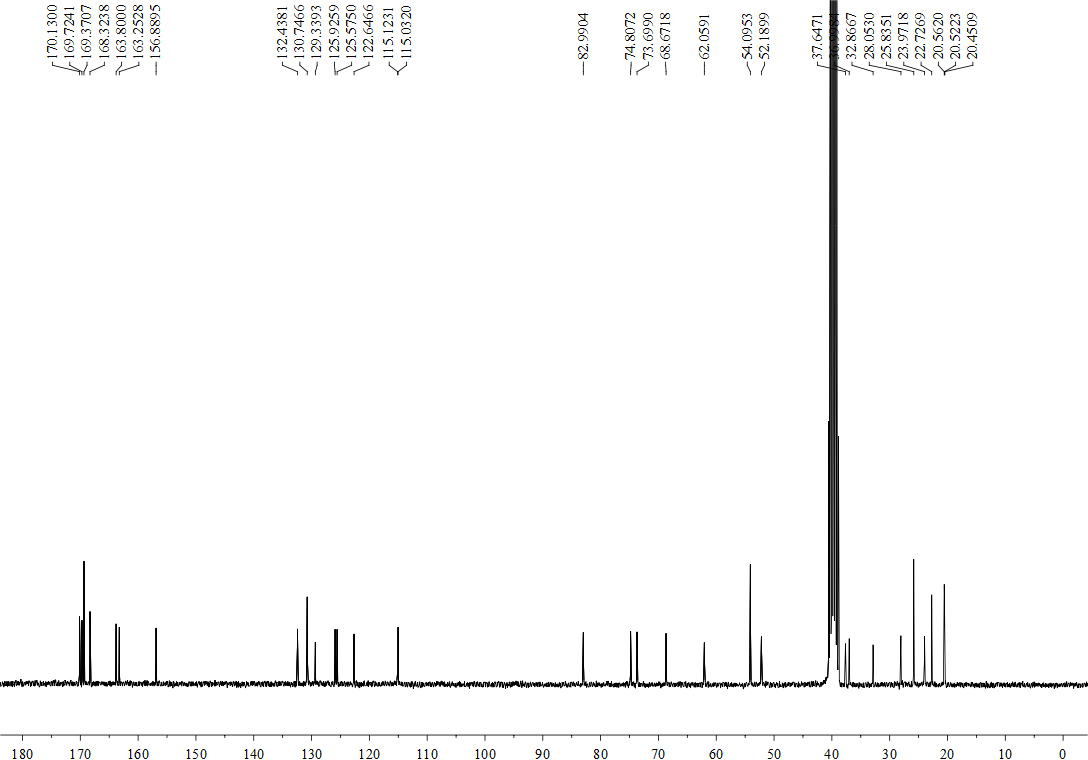


**^1^H NMR spectrum of 10a (300 MHz, DMSO-*d_6_*)**


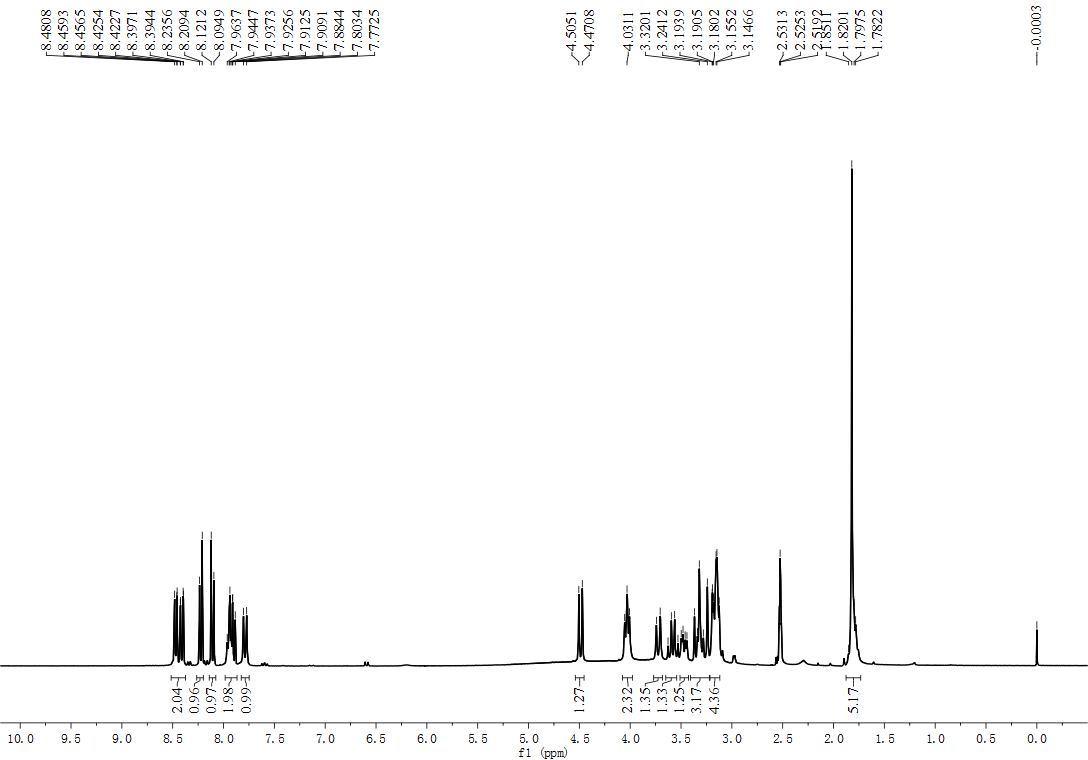


**^13^C NMR spectrum of 10a (300 MHz, DMSO-*d_6_*)**


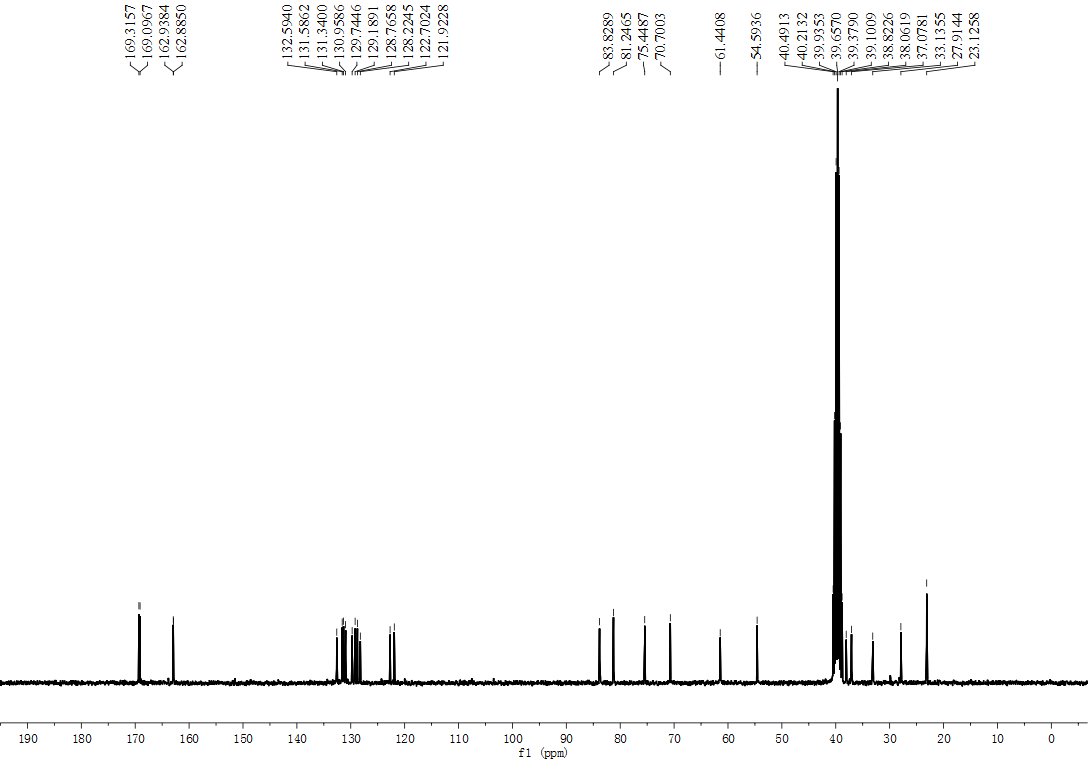


**^1^H NMR spectrum of 10b (300 MHz, DMSO-*d_6_*)**


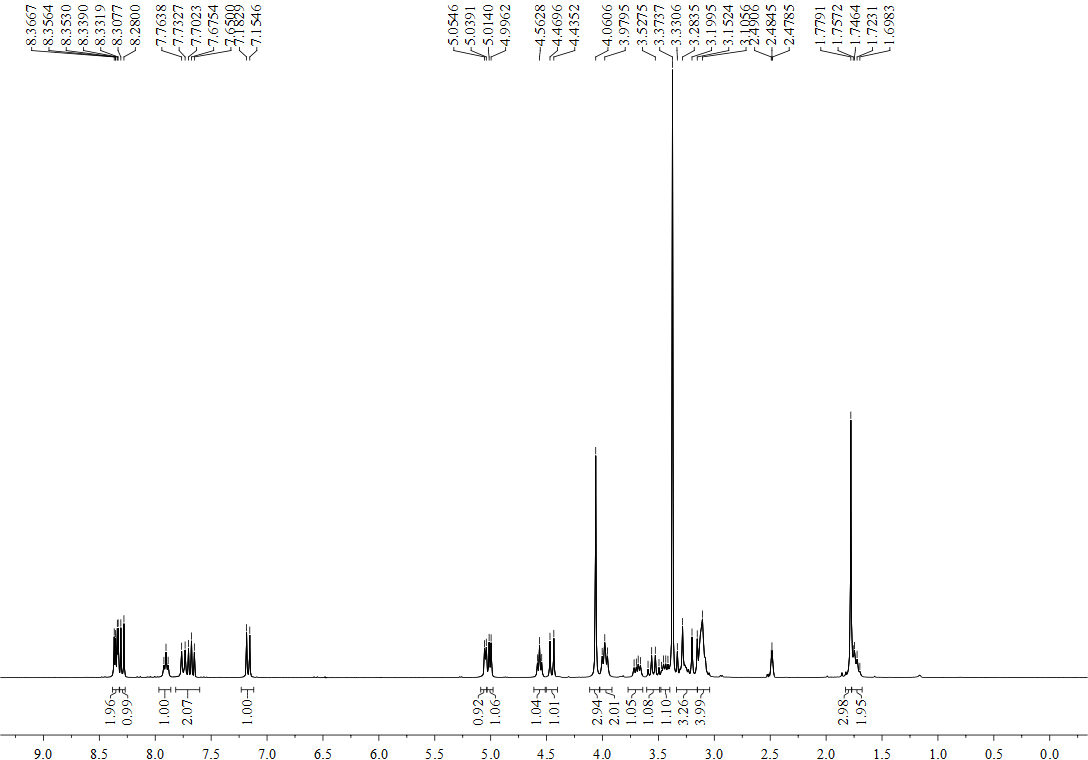


**^13^C NMR spectrum of 10b (300 MHz, DMSO-*d_6_*)**


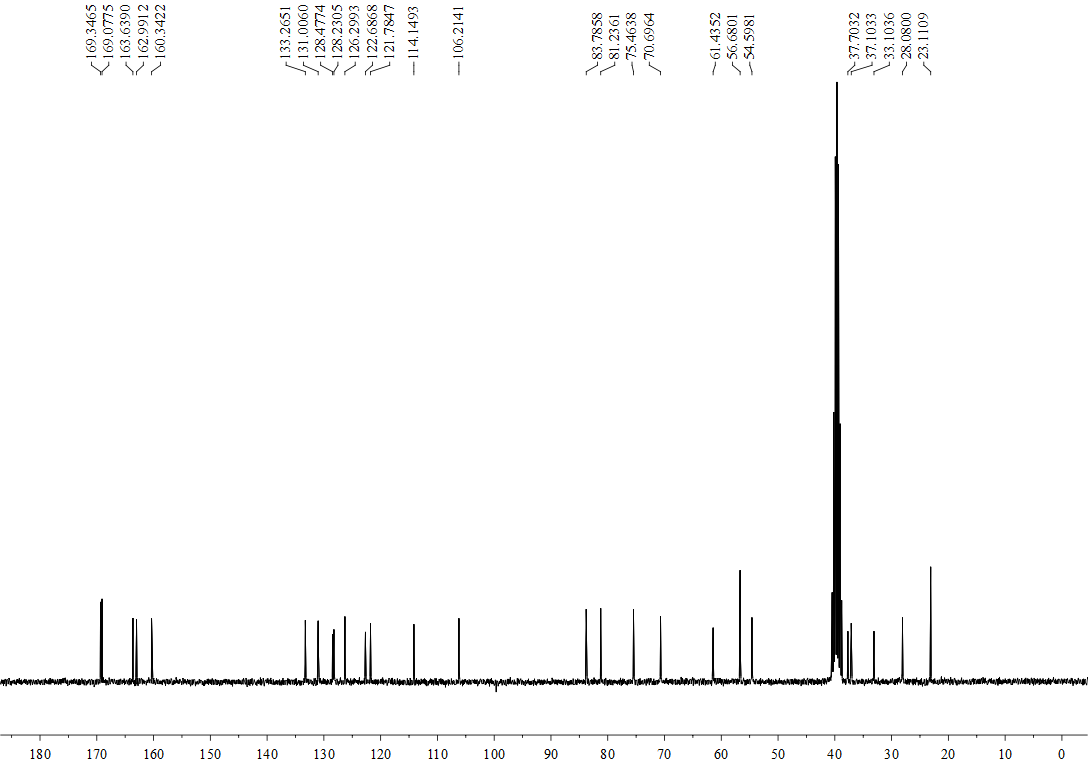


**^1^H NMR spectrum of 10c (300 MHz, DMSO-*d_6_*)**


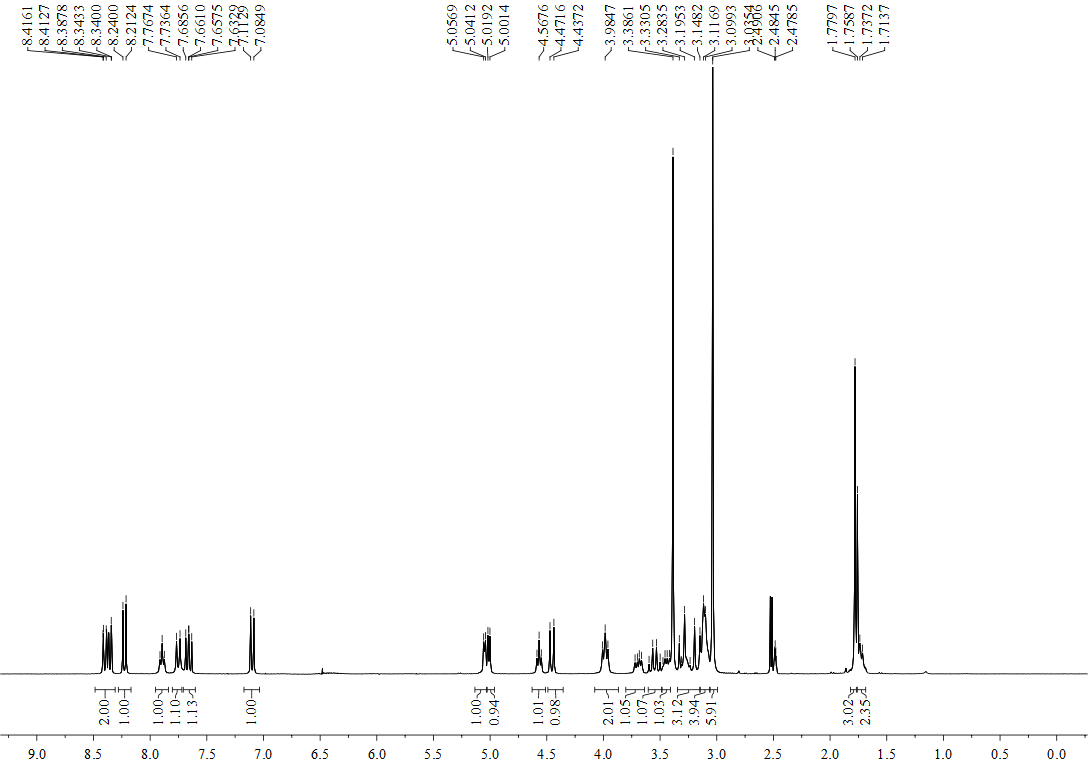


**^13^C NMR spectrum of 10c (300 MHz, DMSO-*d_6_*)**


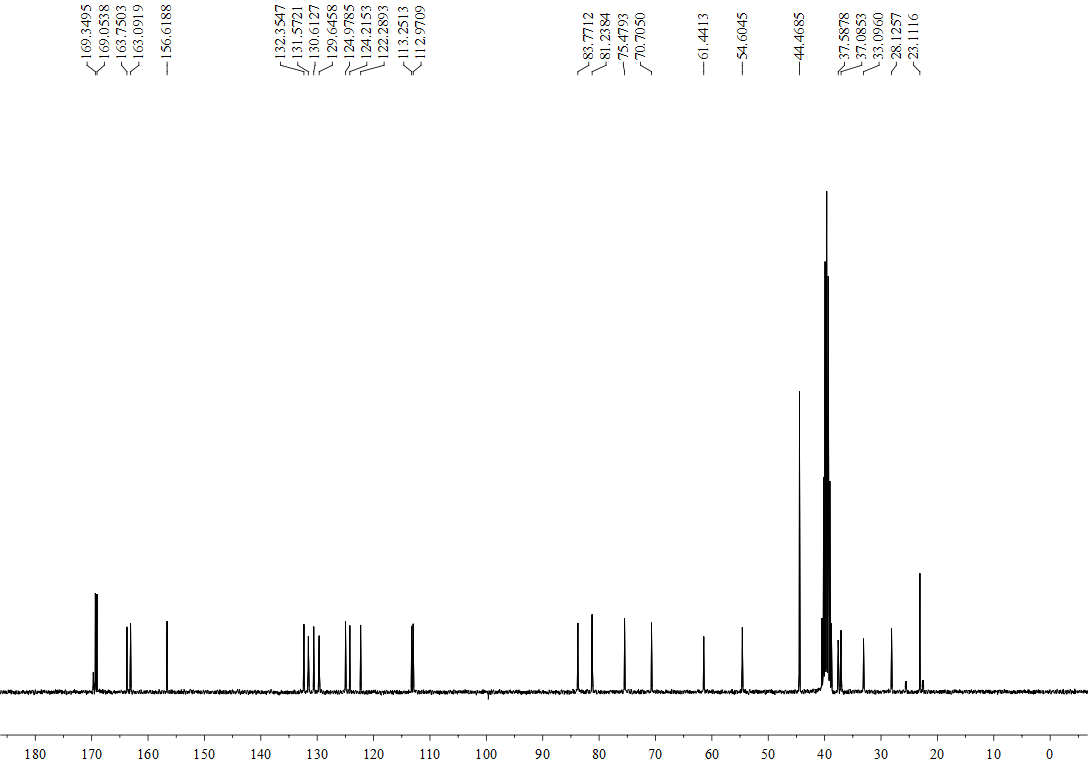


**^1^H NMR spectrum of 10d (300 MHz, DMSO-*d_6_*)**


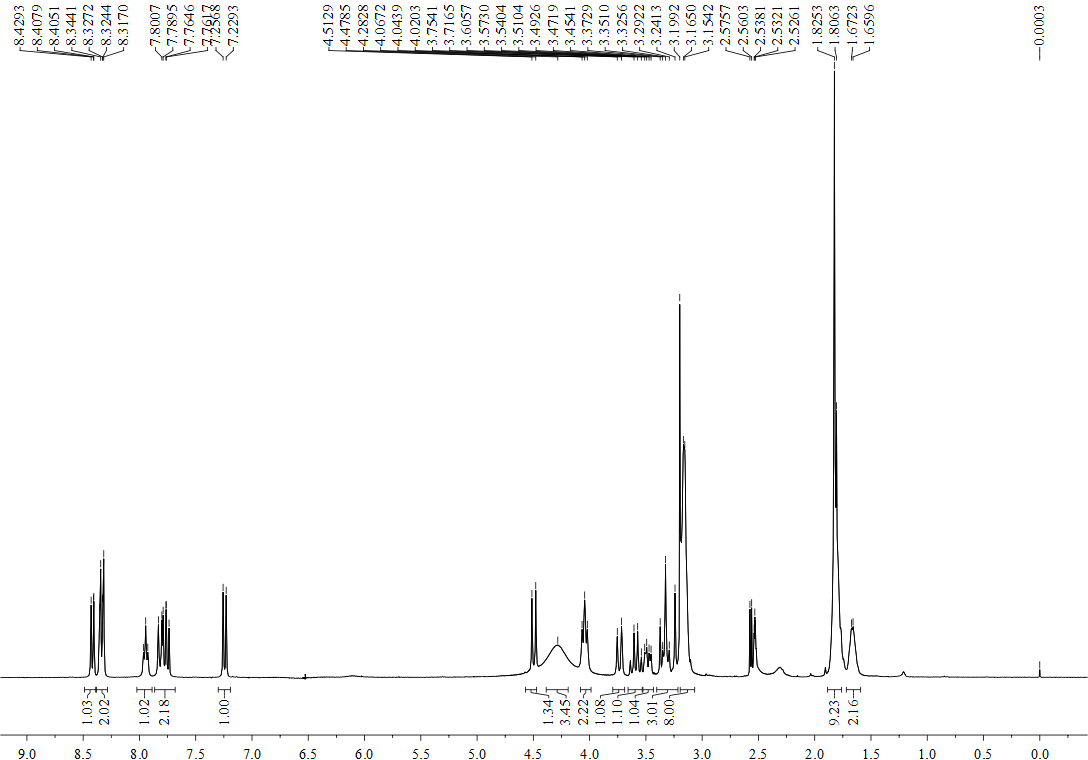


**^13^C NMR spectrum of 10d (300 MHz, DMSO-*d_6_*)**


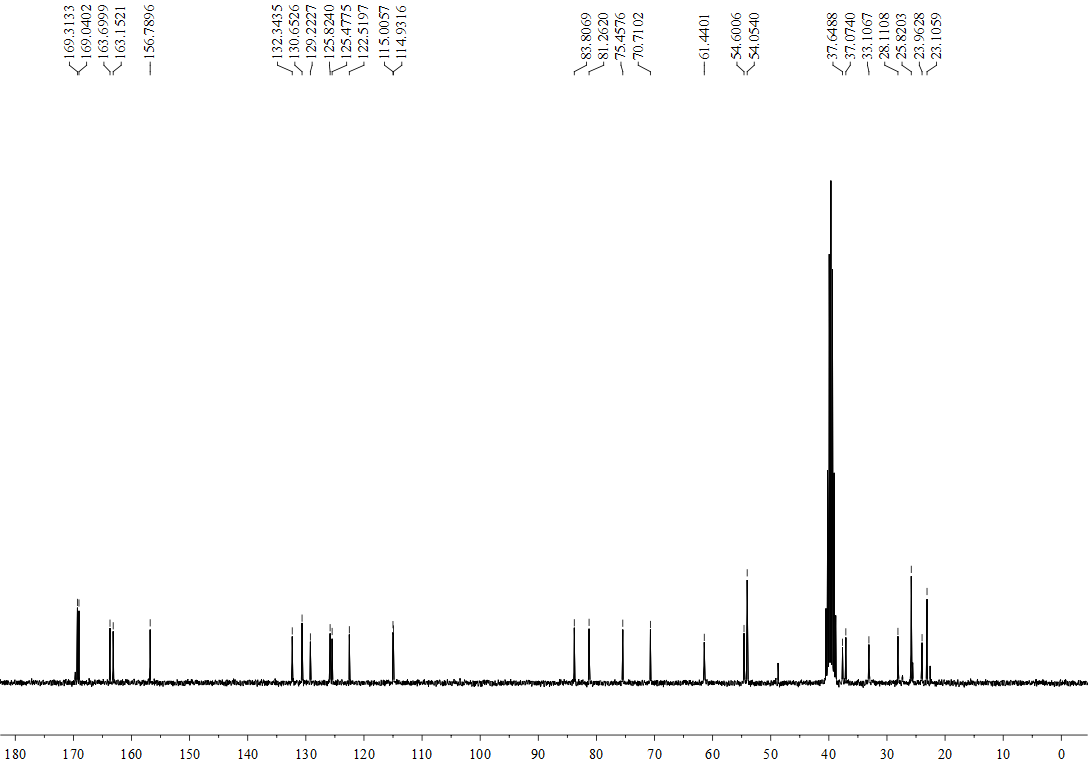


**^1^H NMR spectrum of 15a (300 MHz, DMSO-*d_6_*)**


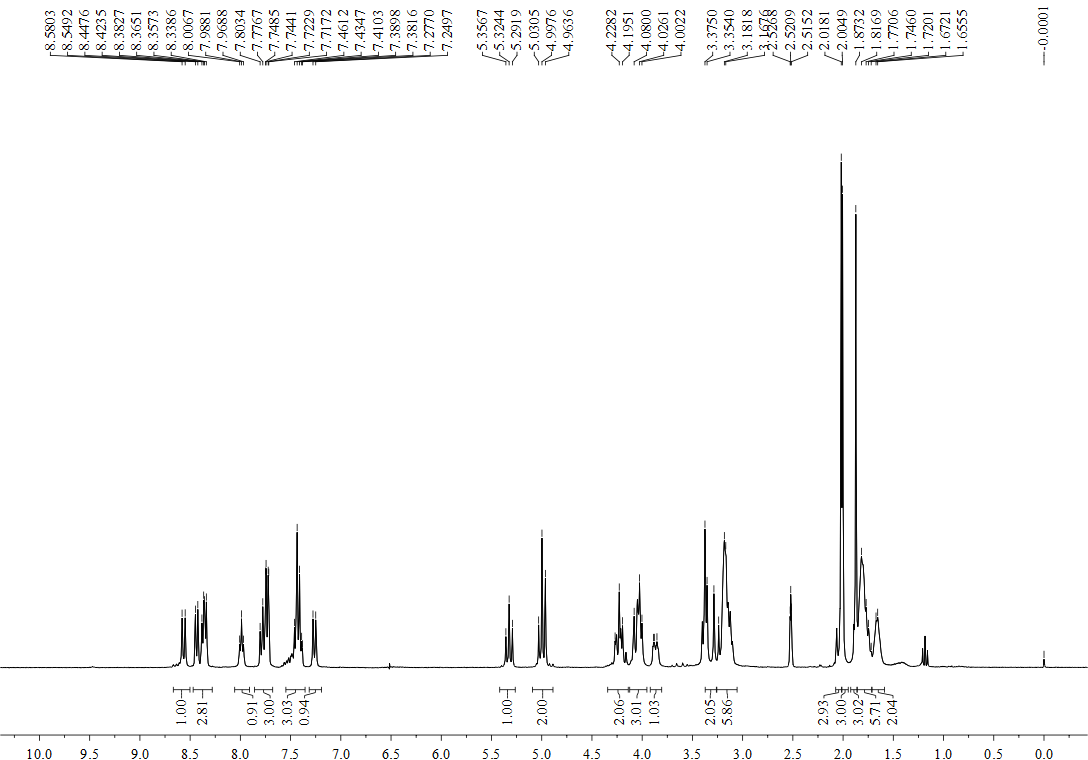


**^13^C NMR spectrum of 15a (300 MHz, DMSO-*d_6_*)**


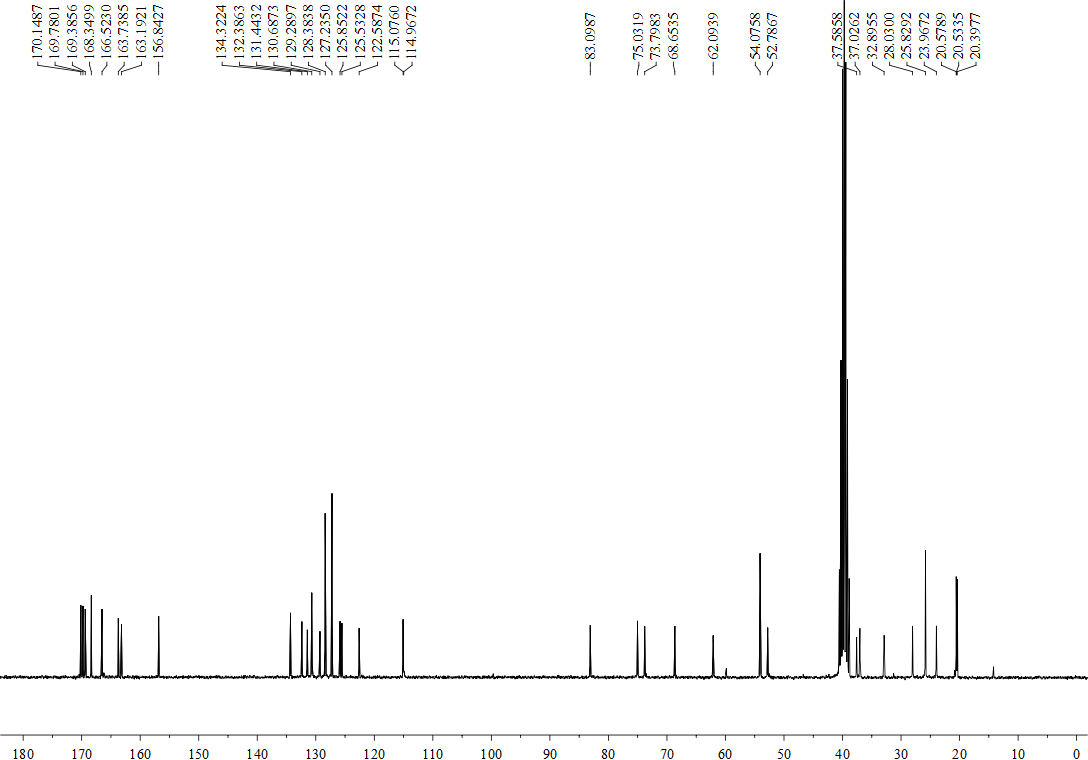


**^1^H NMR spectrum of 15b (300 MHz, DMSO-*d_6_*)**


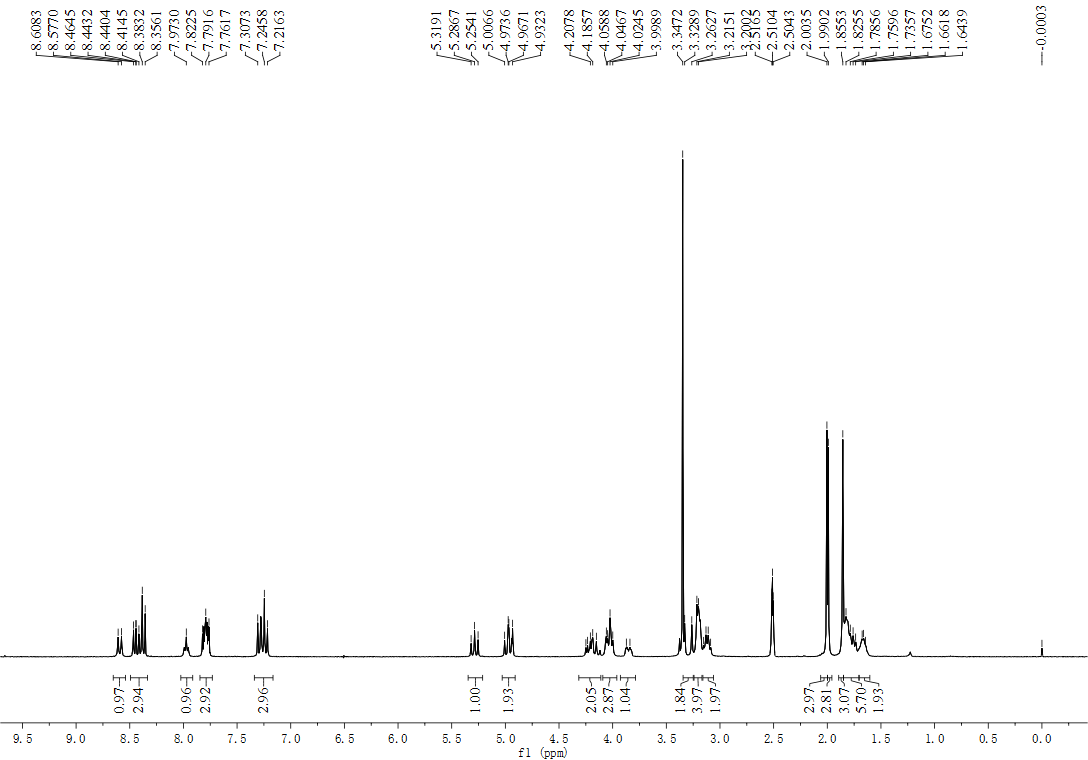


**^13^C NMR spectrum of 15b (300 MHz, DMSO-*d_6_*)**


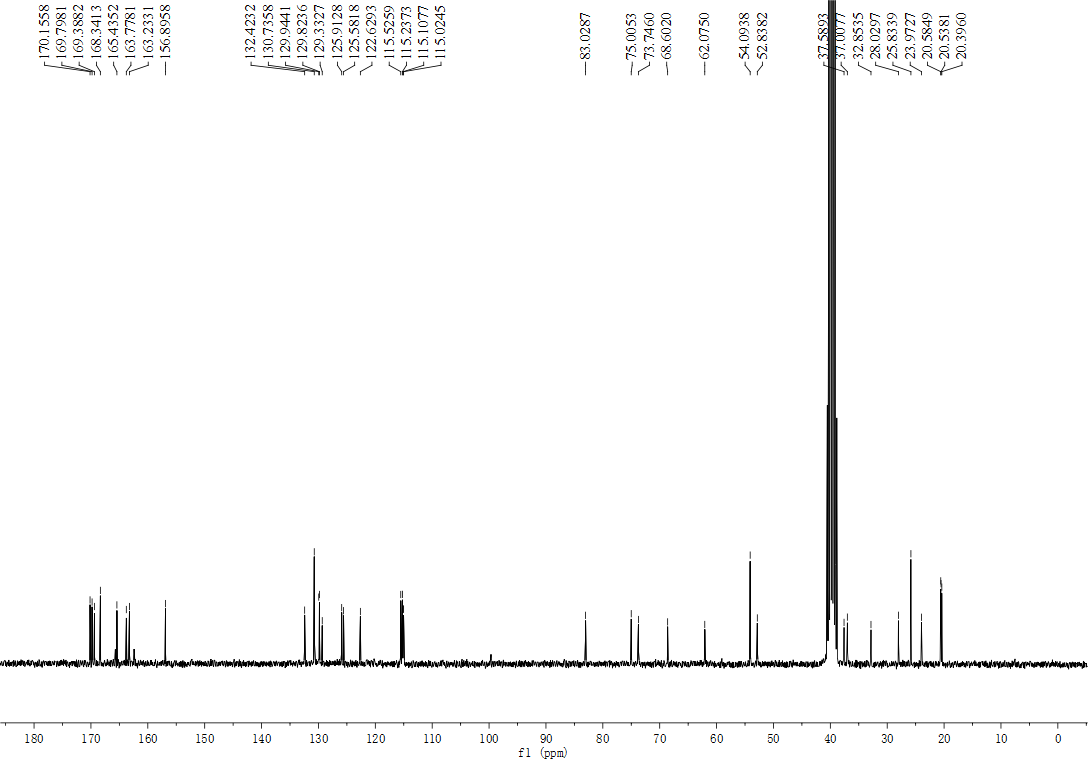


**^1^H NMR spectrum of 15c (300 MHz, DMSO-*d_6_*)**


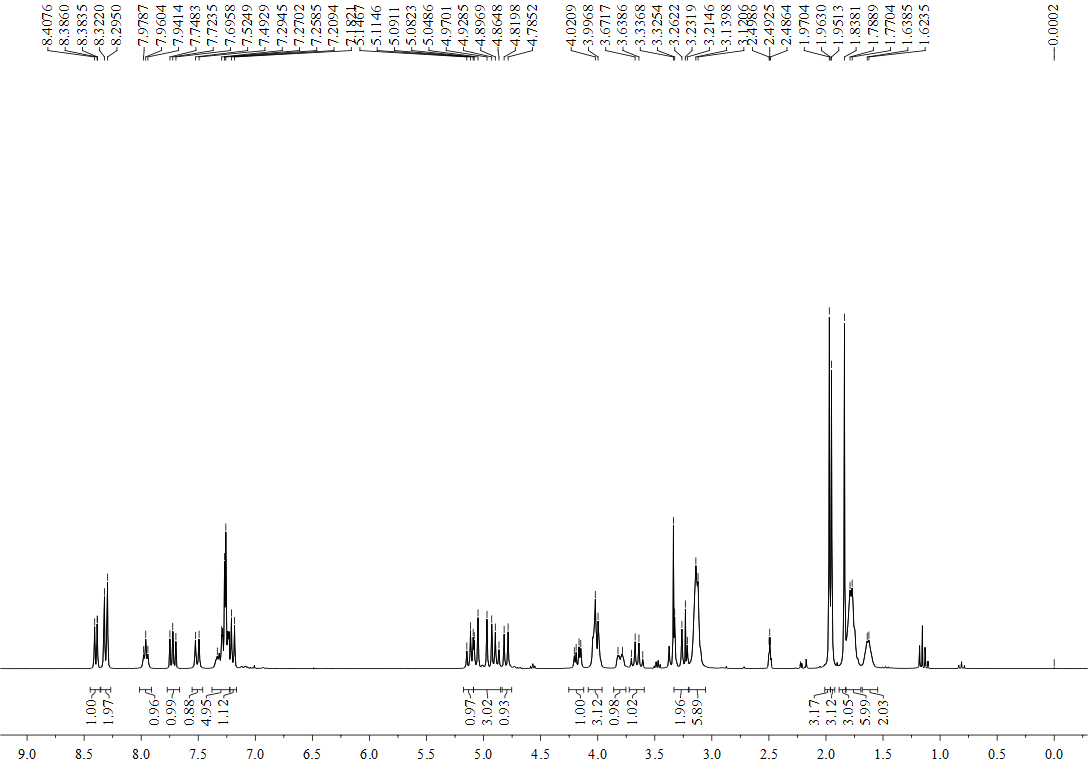


**^13^C NMR spectrum of 15c (300 MHz, DMSO-*d_6_*)**


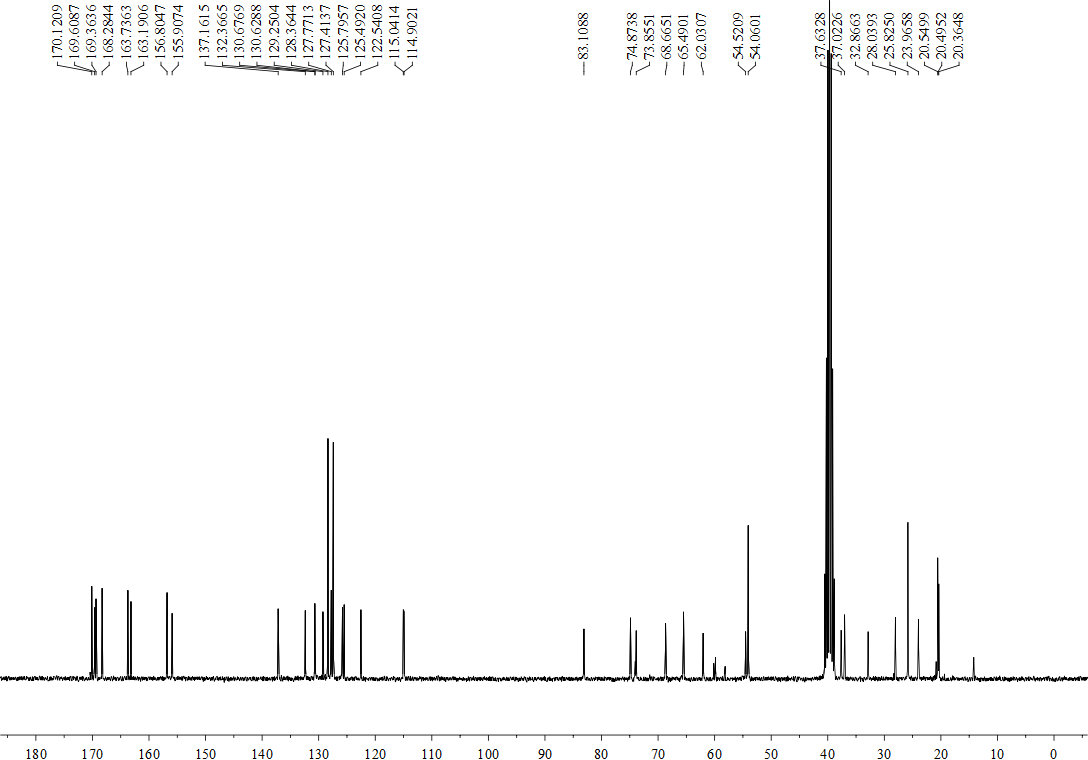


**^1^H NMR spectrum of 15d (300 MHz, DMSO-*d_6_*)**


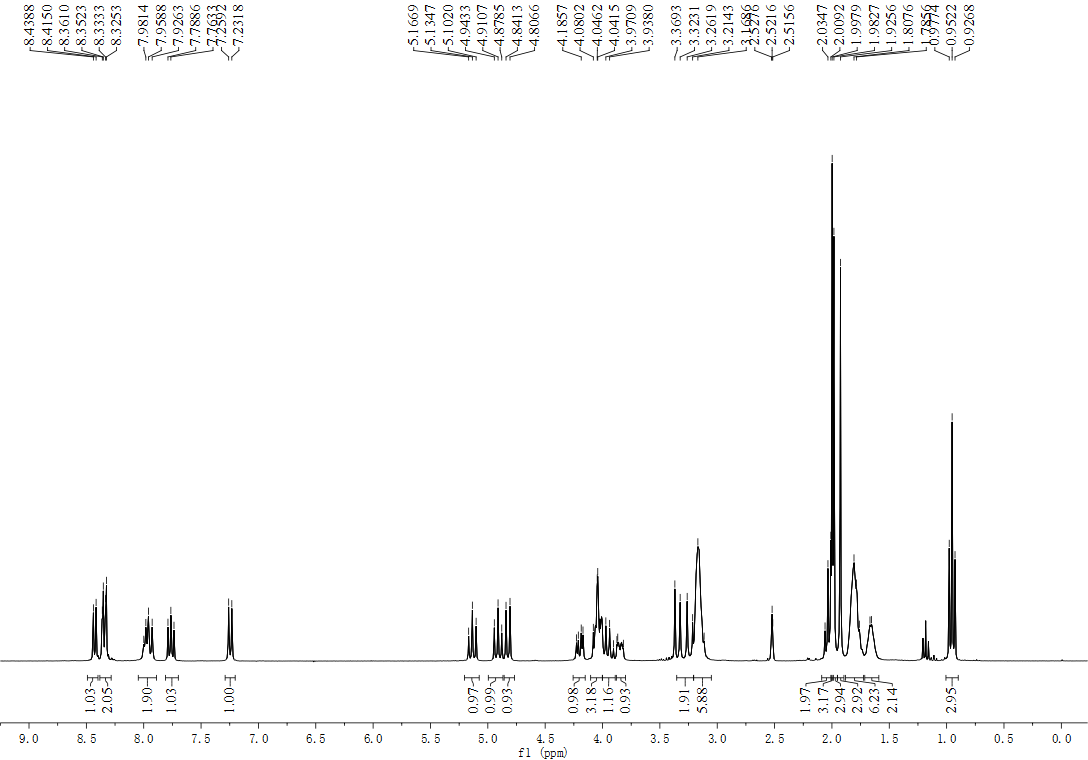


**^13^C NMR spectrum of 15d (300 MHz, DMSO-*d_6_*)**


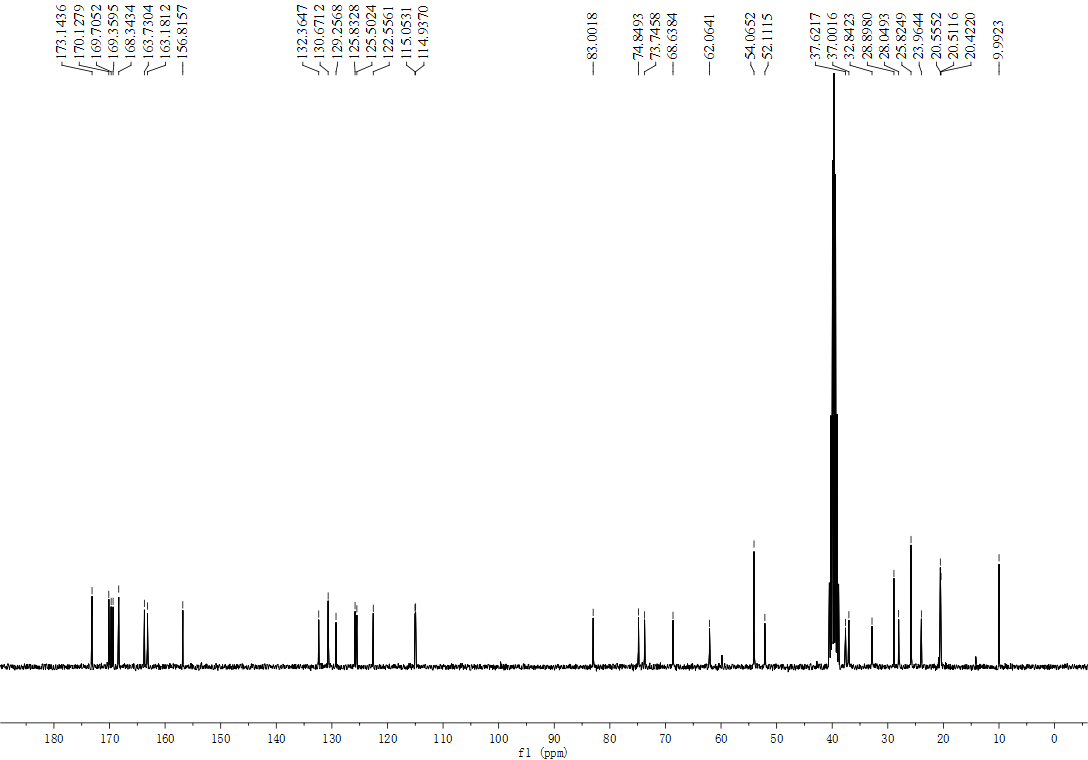


**^1^H NMR spectrum of 15e (300 MHz, DMSO-*d_6_*)**


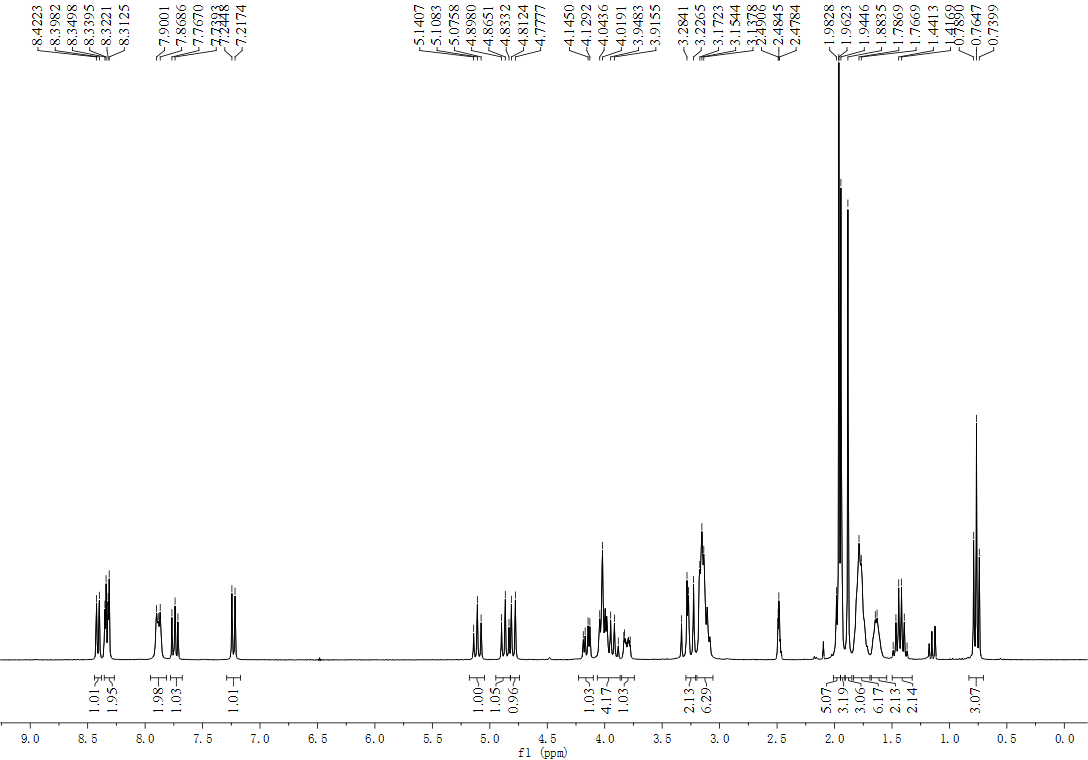


**^13^C NMR spectrum of 15e (300 MHz, DMSO-*d_6_*)**


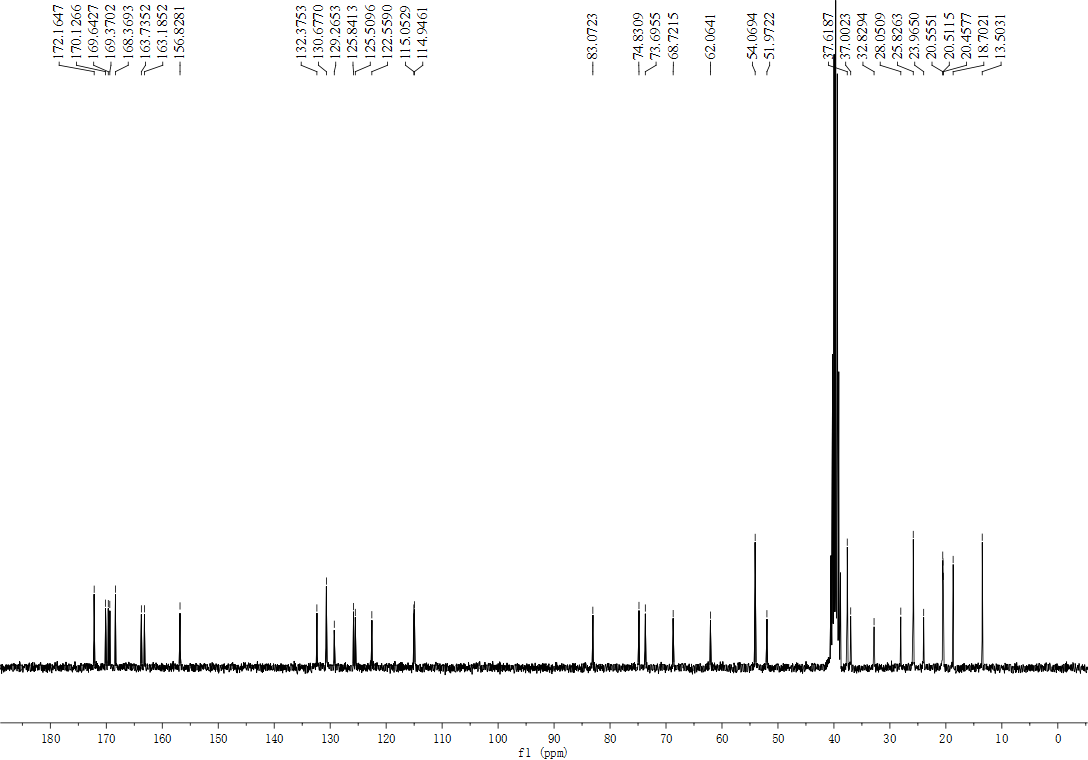


**^1^H NMR spectrum of 15f (300 MHz, DMSO-*d_6_*)**


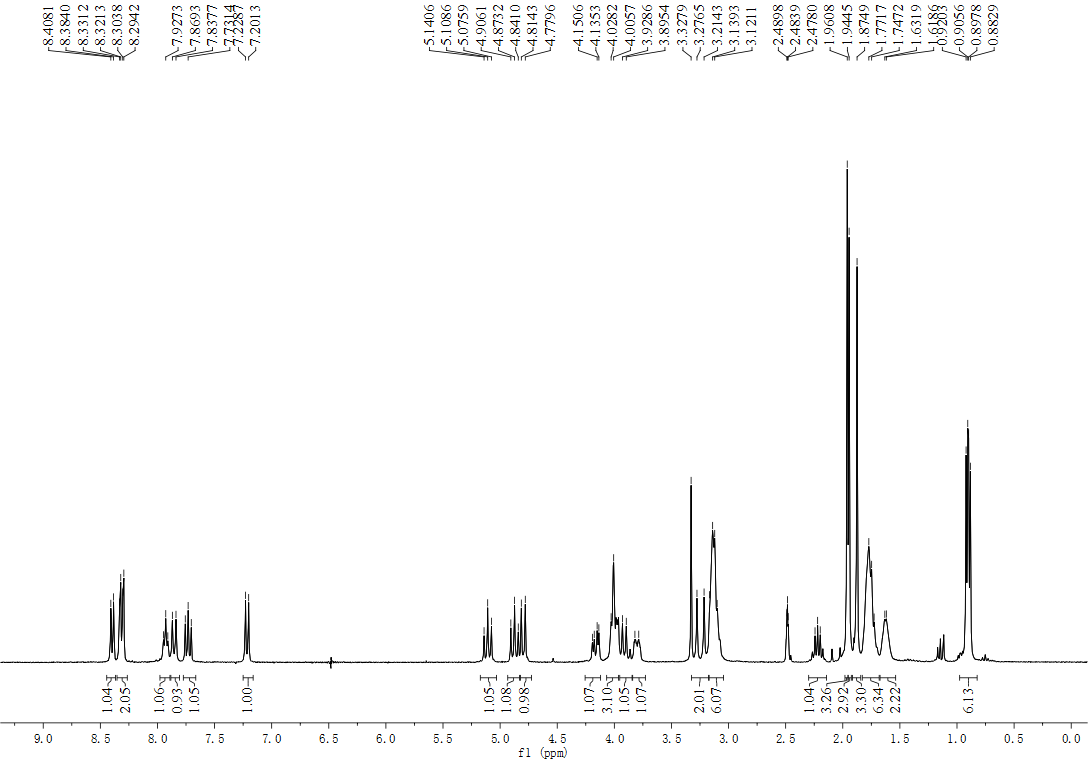


**^13^C NMR spectrum of 15f (300 MHz, DMSO-*d_6_*)**


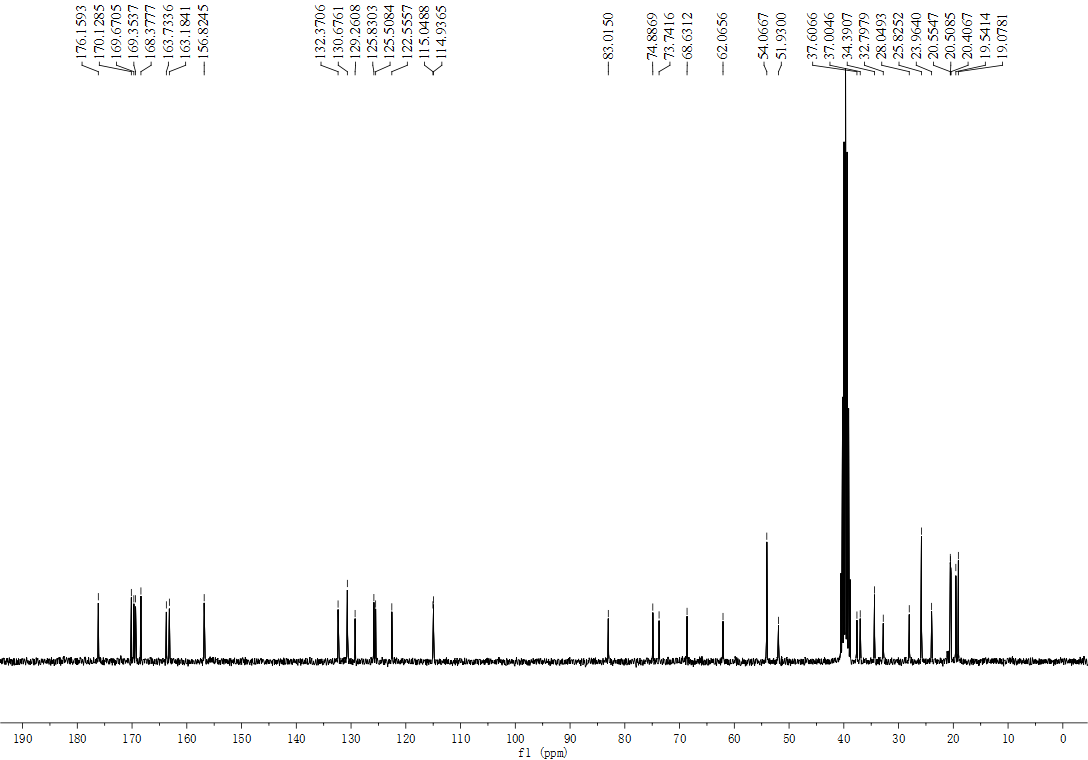


**^1^H NMR spectrum of 15g (300 MHz, DMSO-*d_6_*)**


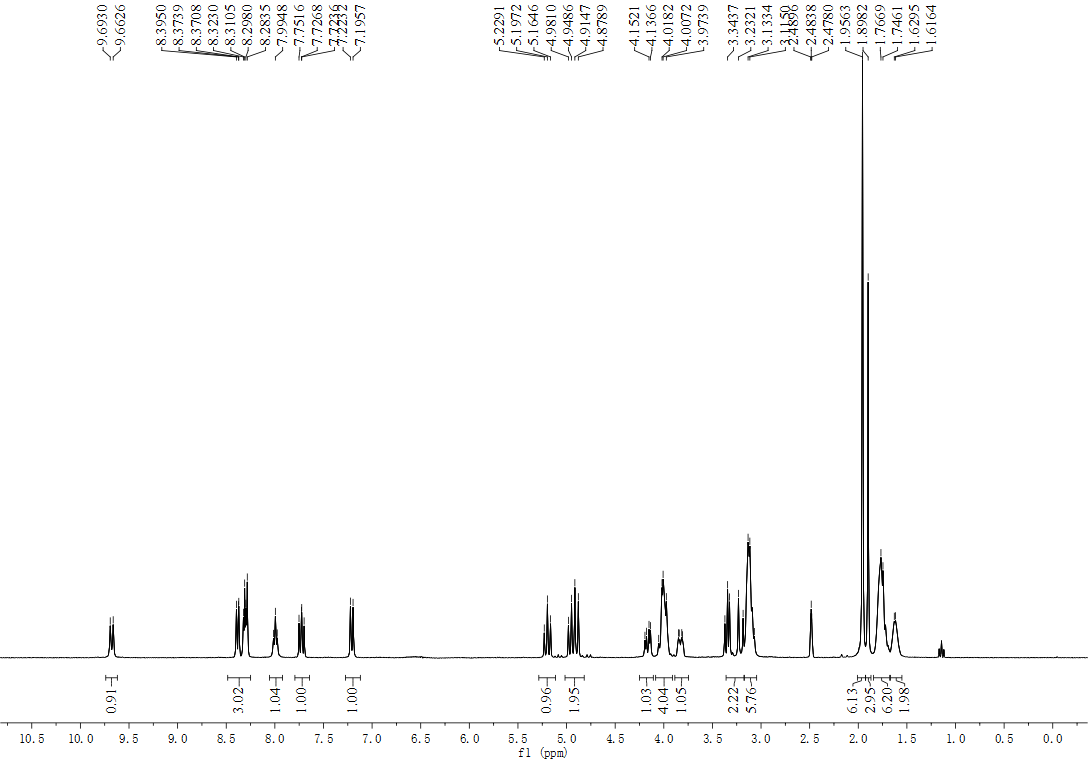


**^13^C NMR spectrum of 15g (300 MHz, DMSO-*d_6_*)**


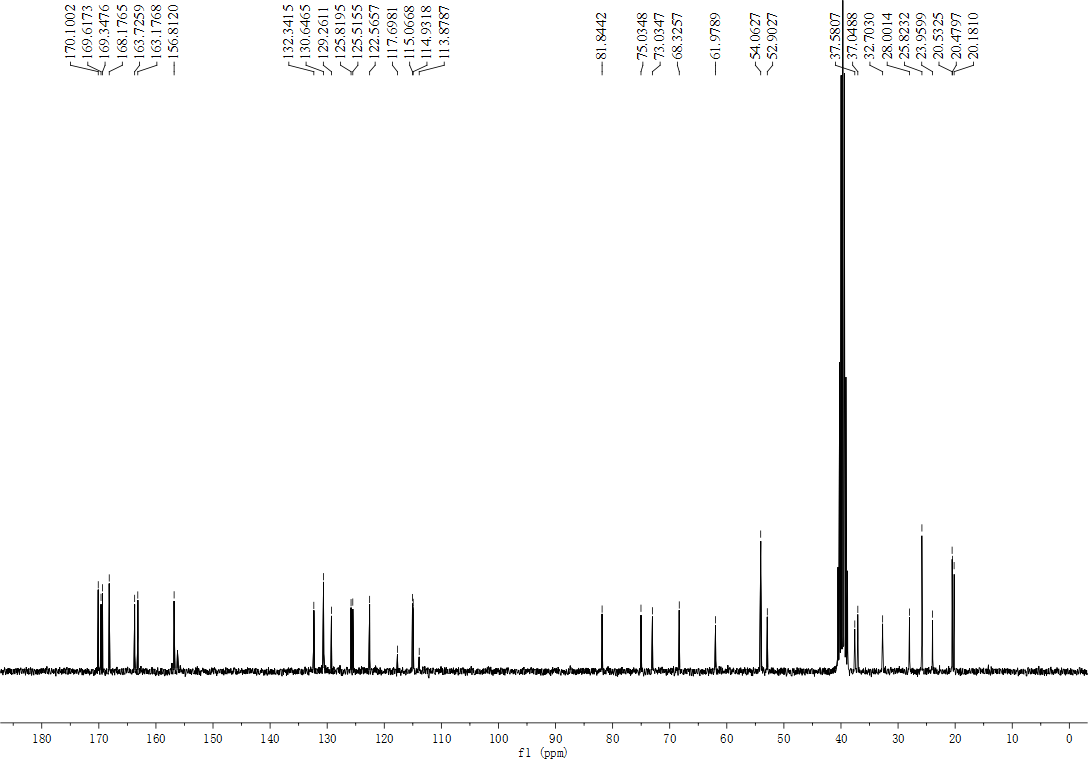


**^1^H NMR spectrum of 15h (300 MHz, DMSO-*d_6_*)**


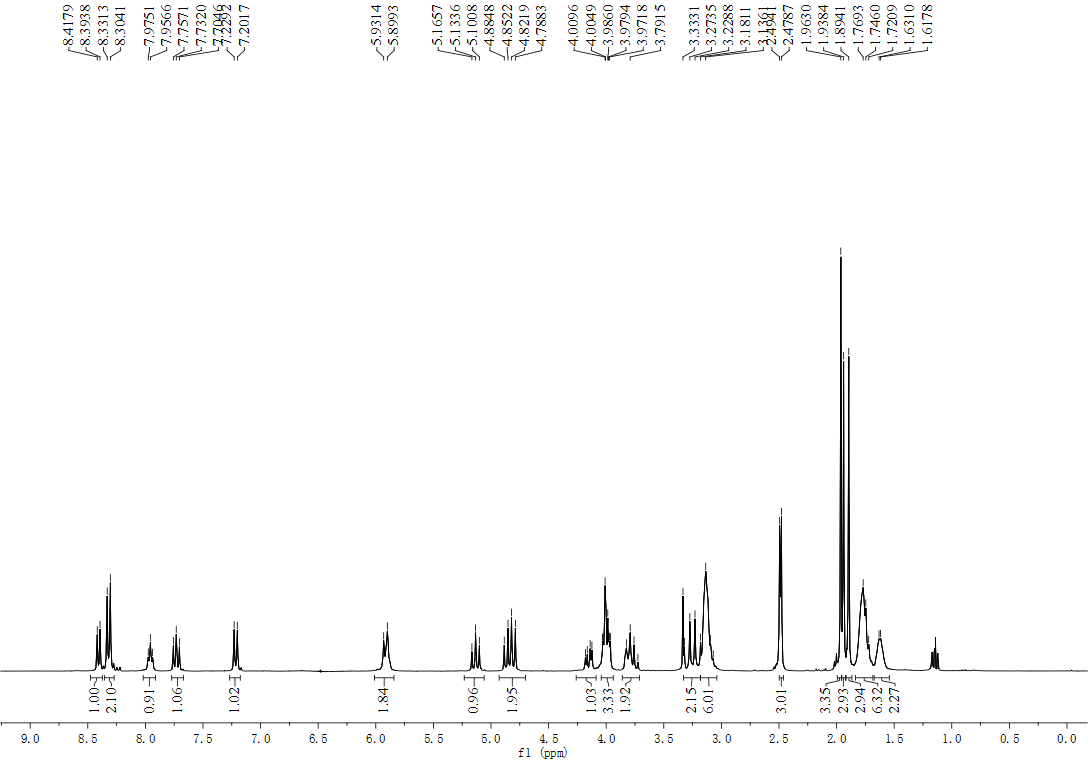


**^13^C NMR spectrum of 15h (300 MHz, DMSO-*d_6_*)**


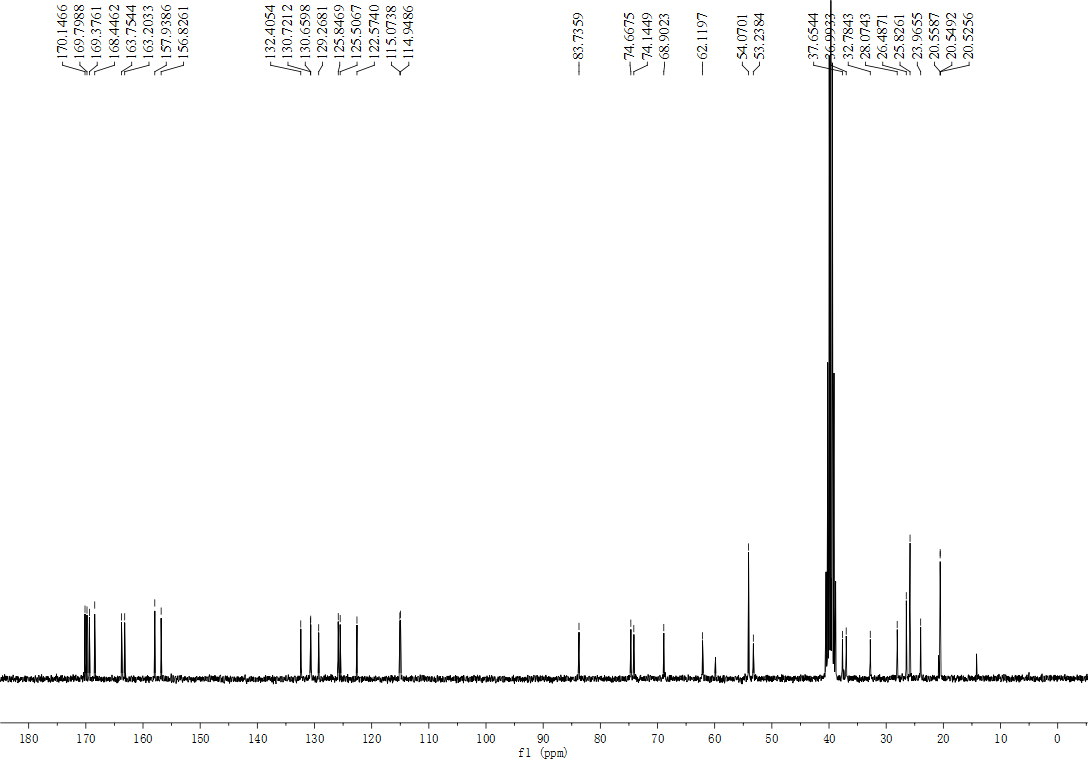


**^1^H NMR spectrum of 15i (300 MHz, DMSO-*d_6_*)**


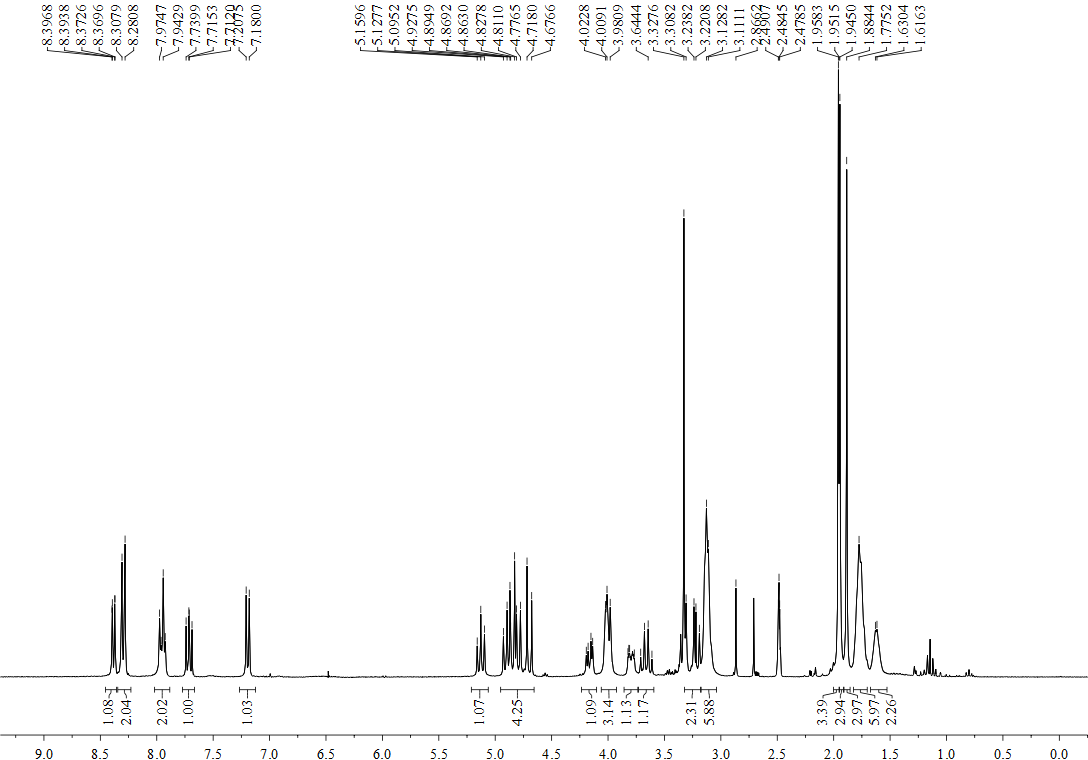


**^13^C NMR spectrum of 15i (300 MHz, DMSO-*d_6_*)**


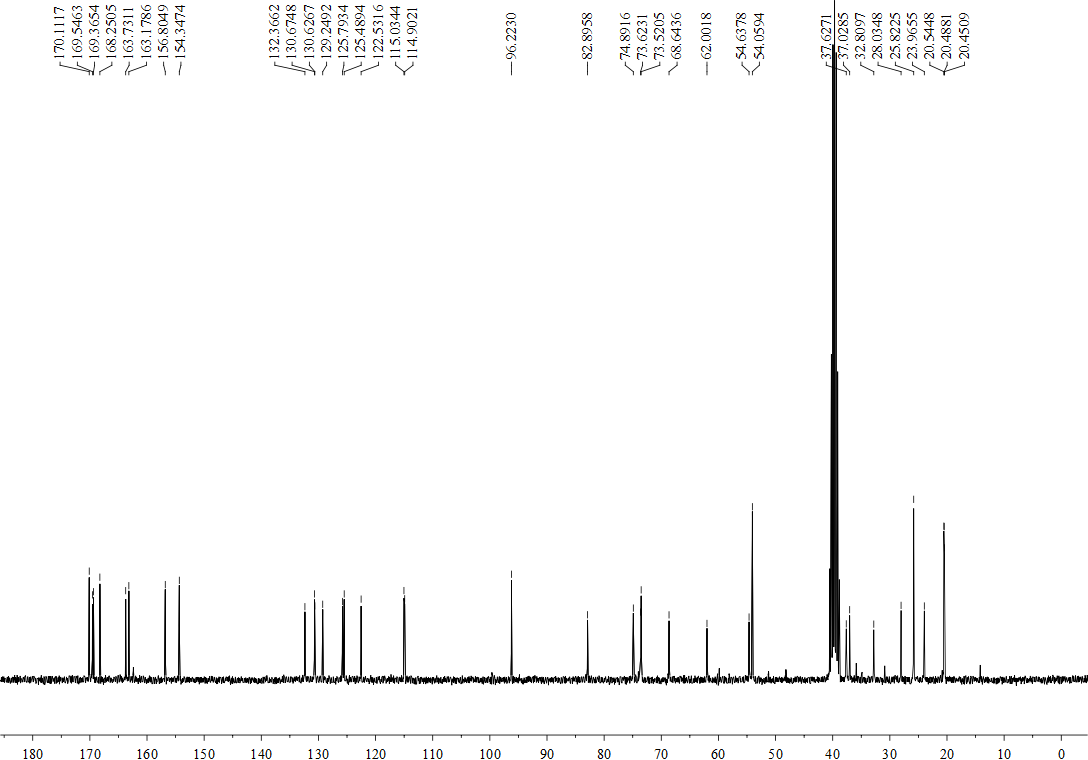


**^1^H NMR spectrum of 15j (300 MHz, DMSO-*d_6_*)**


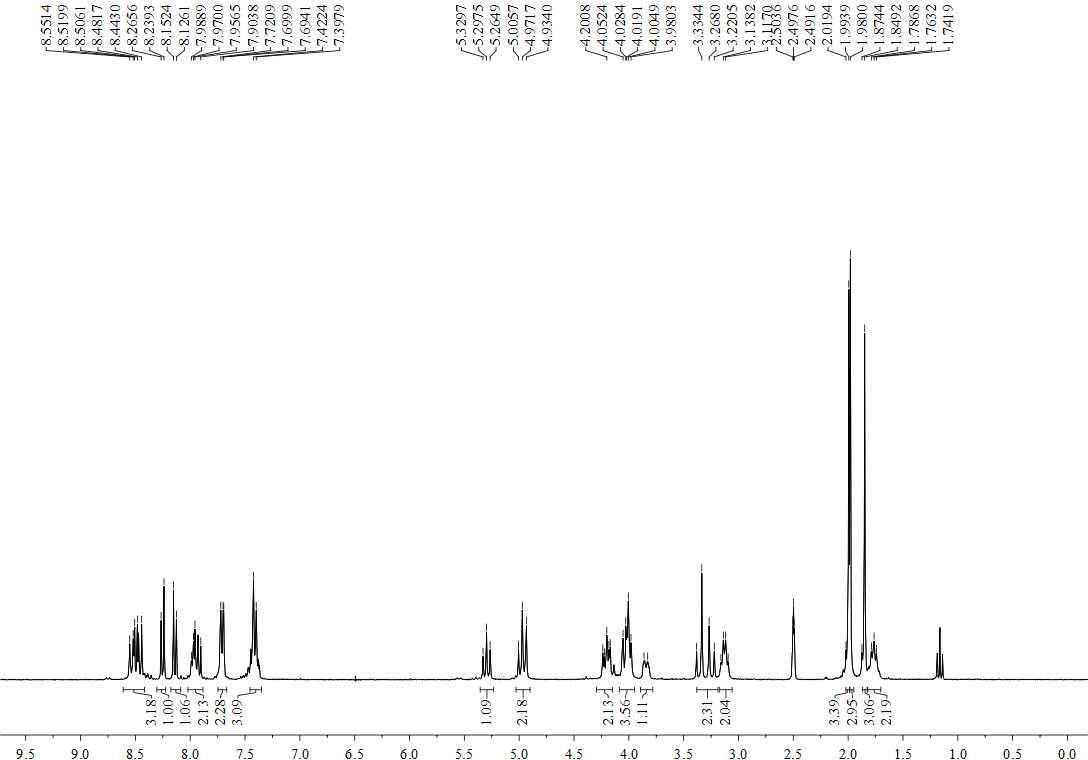


**^13^C NMR spectrum of 15j (300 MHz, DMSO-*d_6_*)**


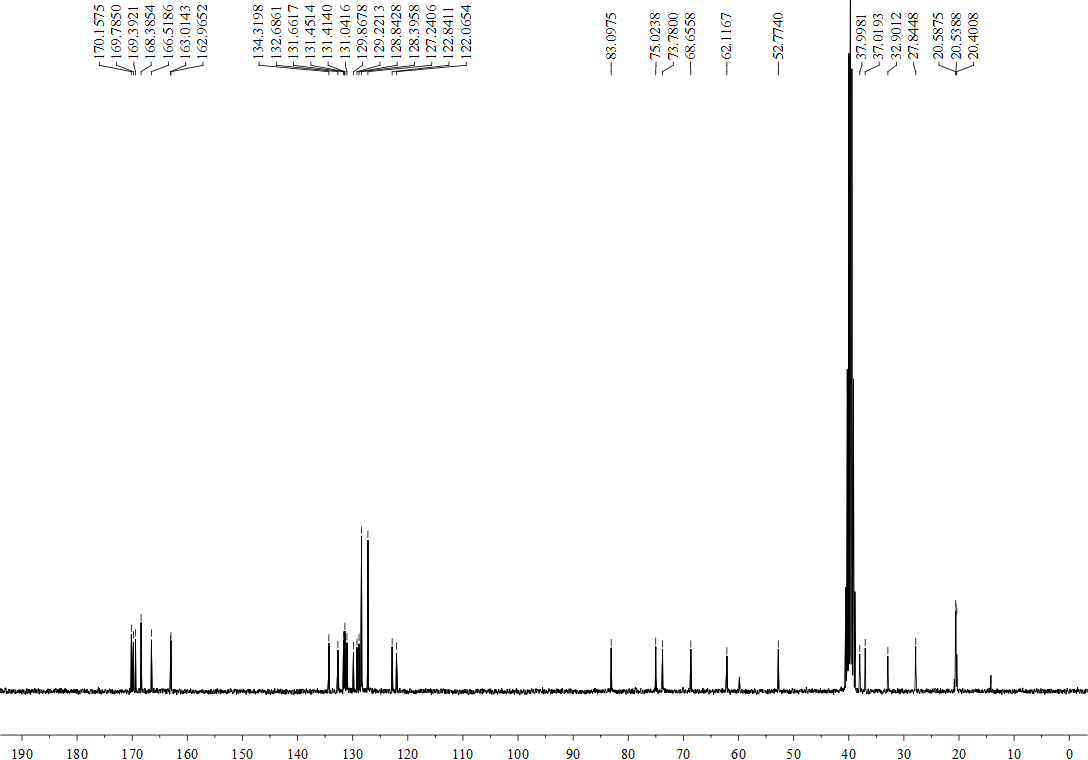


**^1^H NMR spectrum of 15k (300 MHz, DMSO-*d_6_*)**


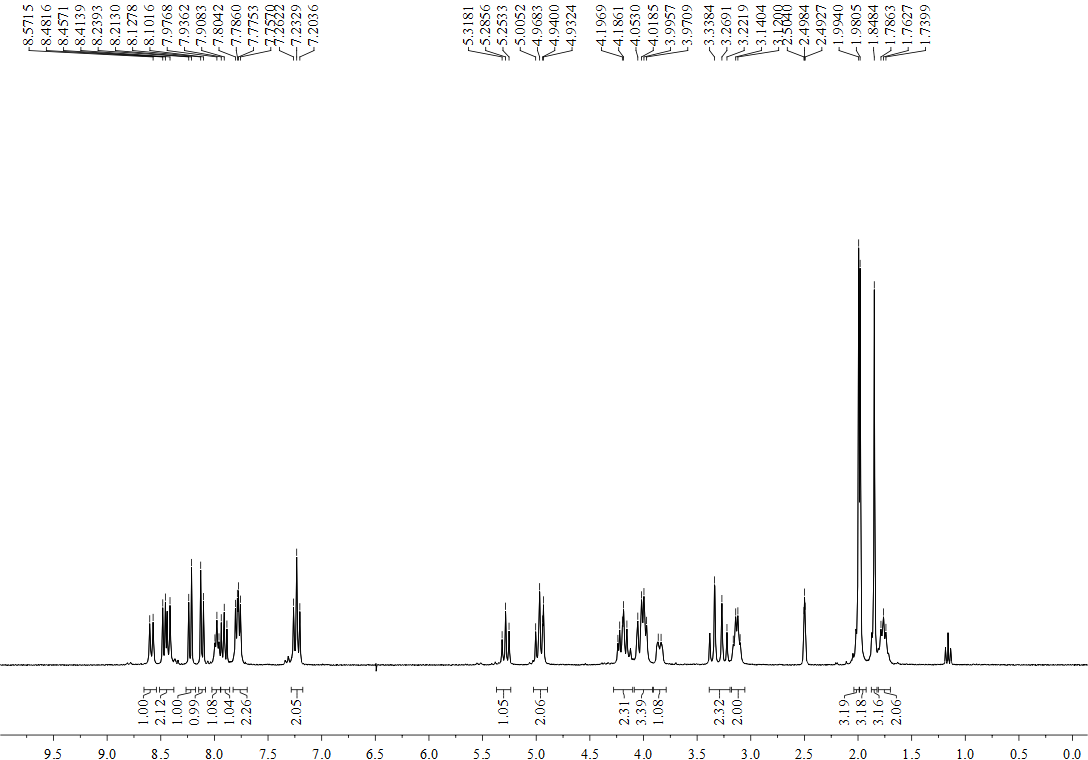


**^13^C NMR spectrum of 15k (300 MHz, DMSO-*d_6_*)**


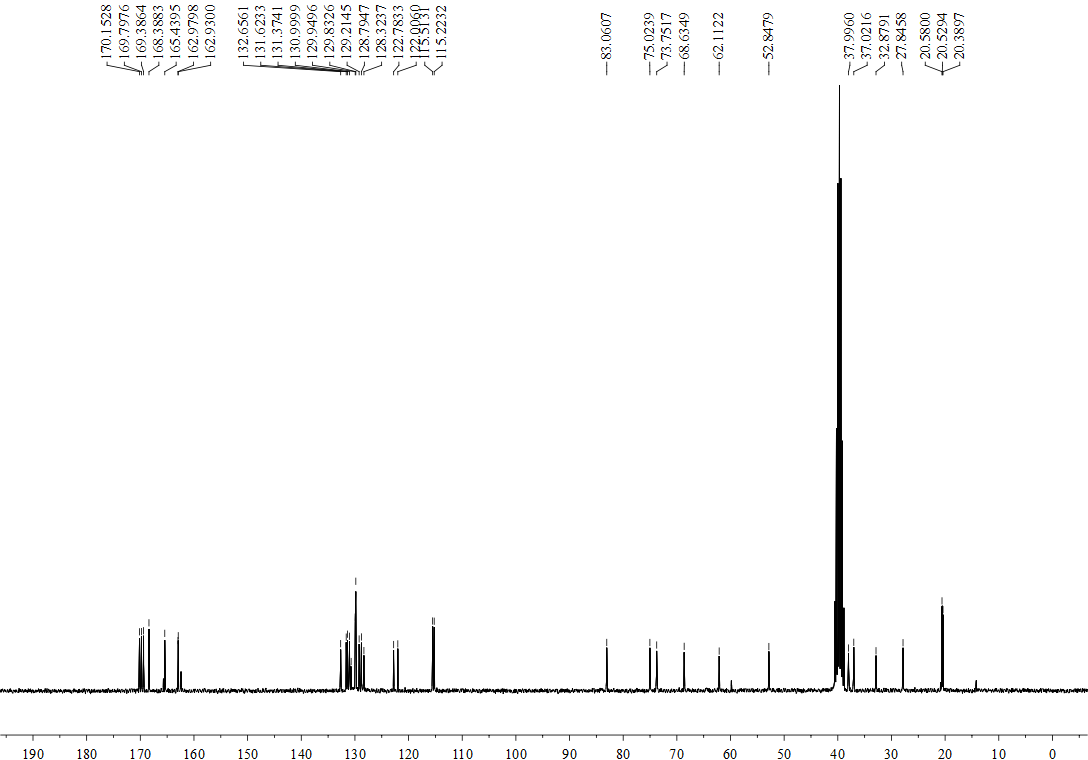


**^1^H NMR spectrum of 15l (300 MHz, DMSO-*d_6_*)**


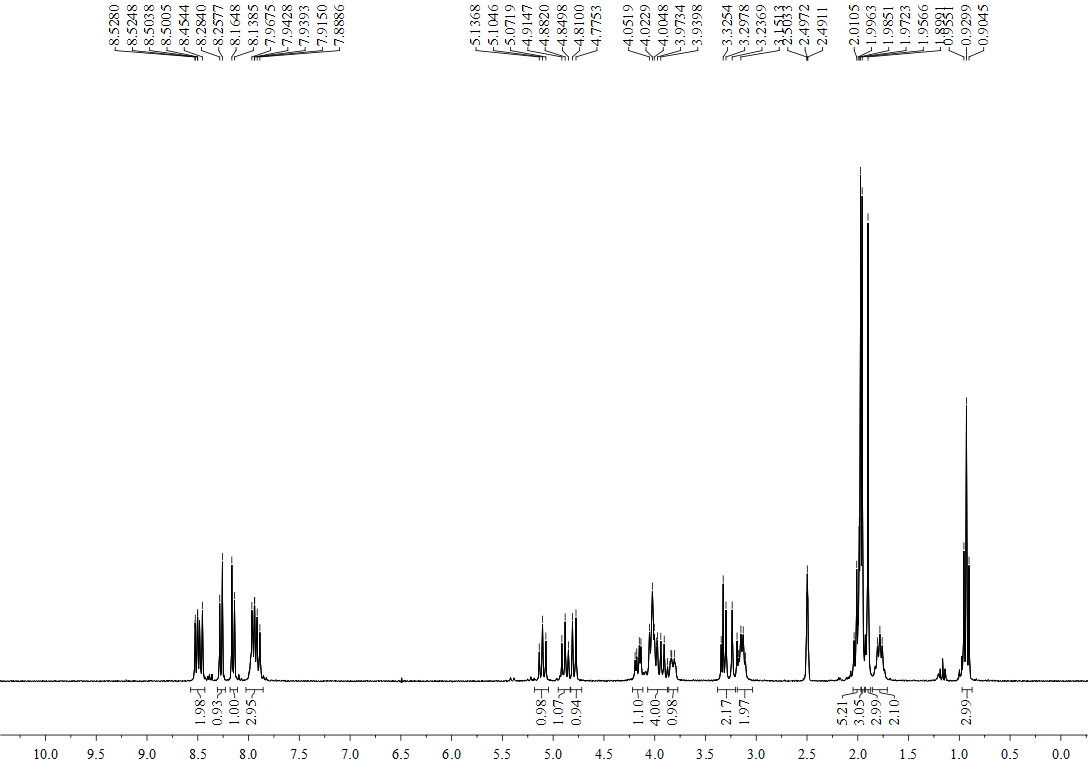


**^13^C NMR spectrum of 15l (300 MHz, DMSO-*d_6_*)**


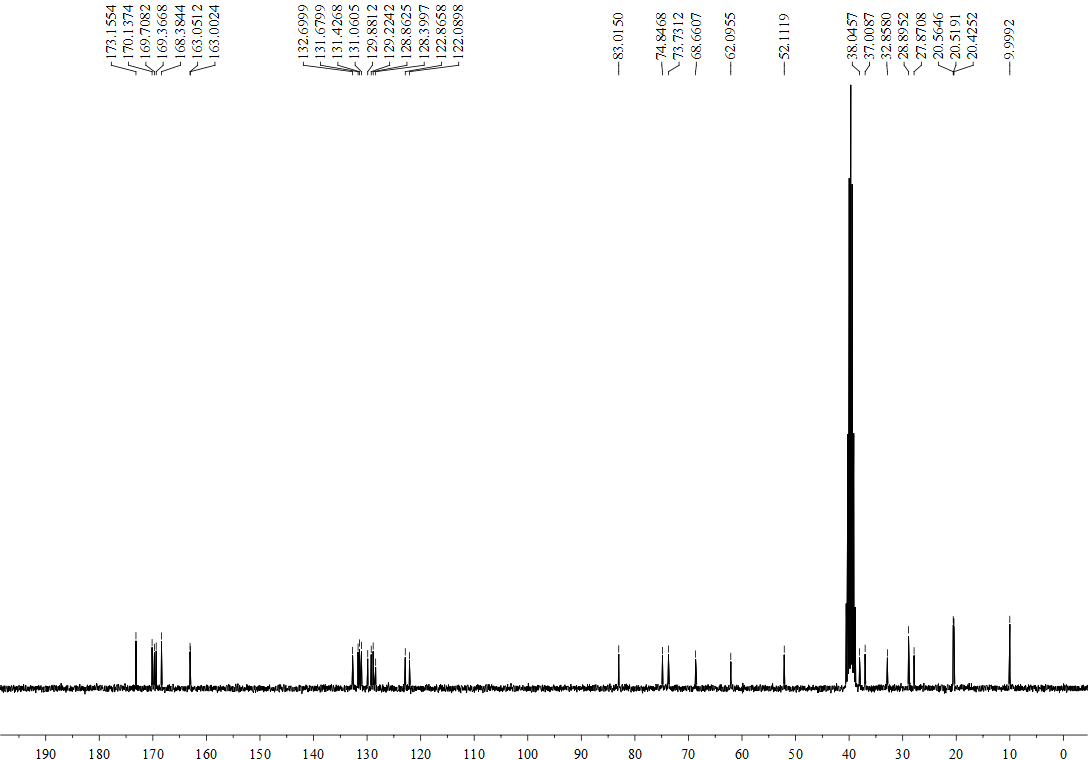


**^1^H NMR spectrum of 15m (300 MHz, DMSO-*d_6_*)**


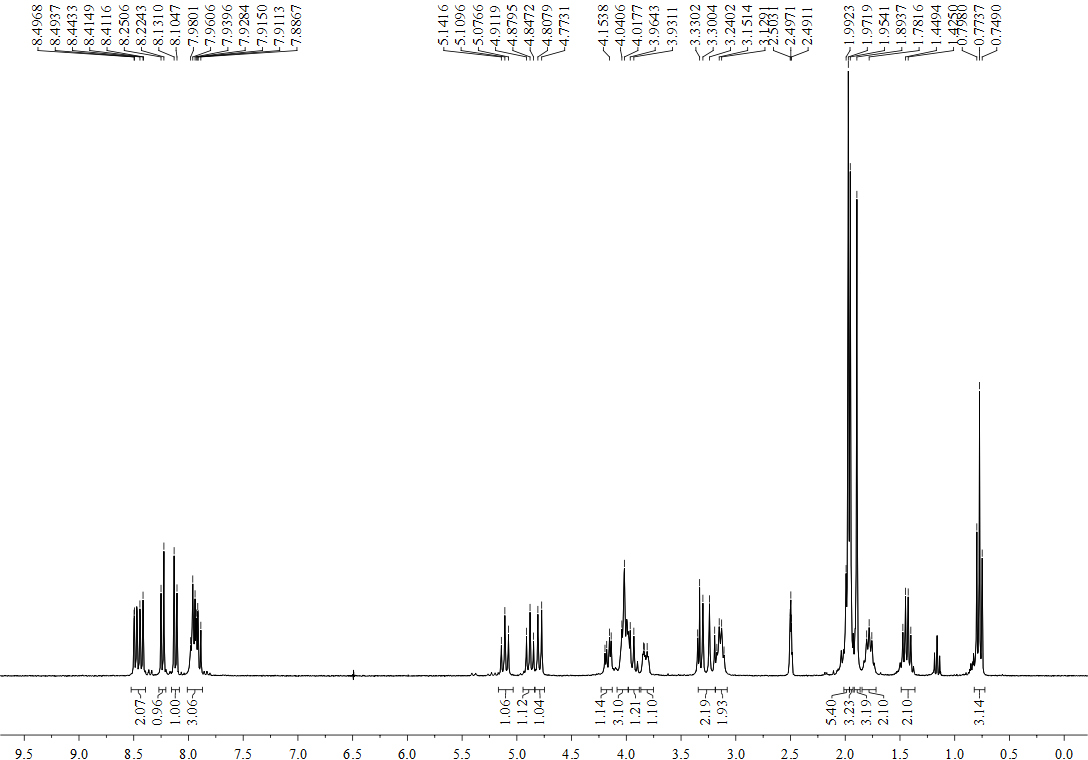


**^13^C NMR spectrum of 15m (300 MHz, DMSO-*d_6_*)**


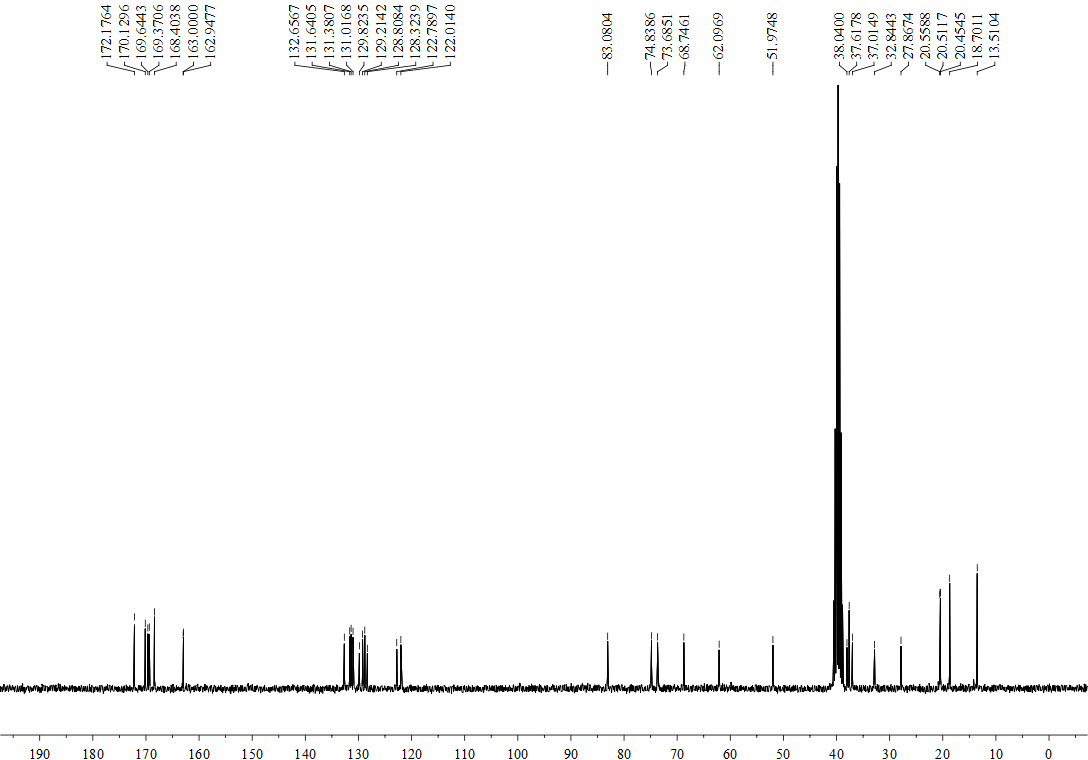


**^1^H NMR spectrum of 15n (300 MHz, DMSO-*d_6_*)**


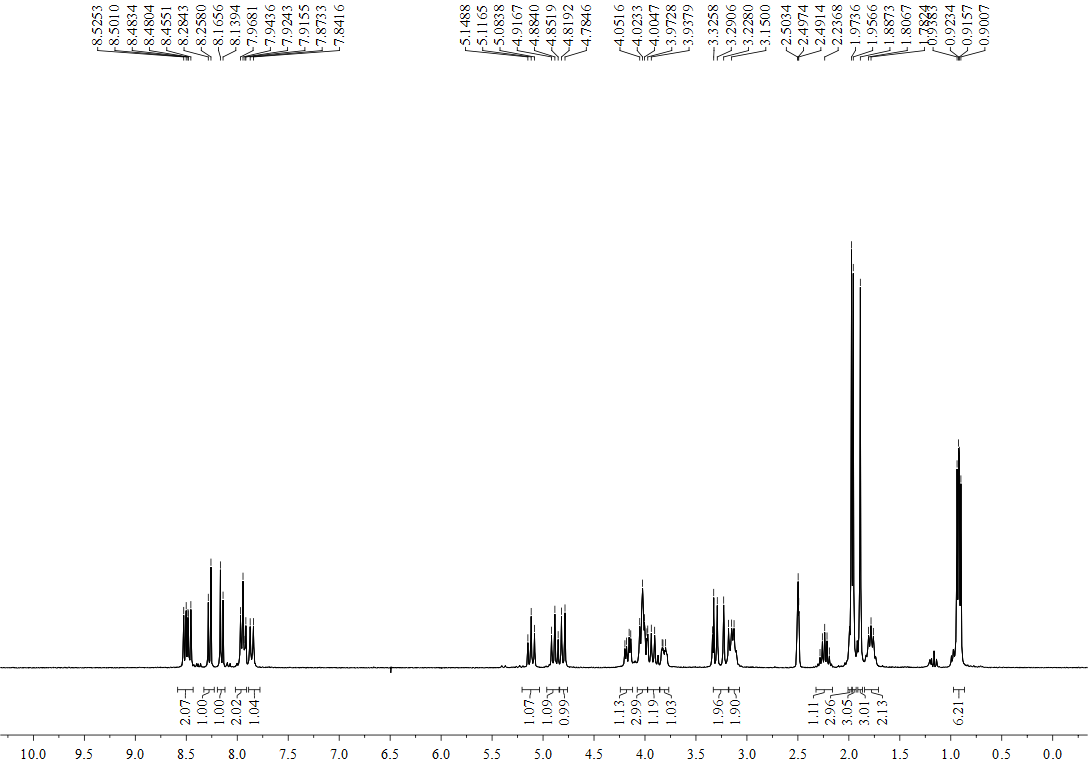


**^13^C NMR spectrum of 15n (300 MHz, DMSO-*d_6_*)**


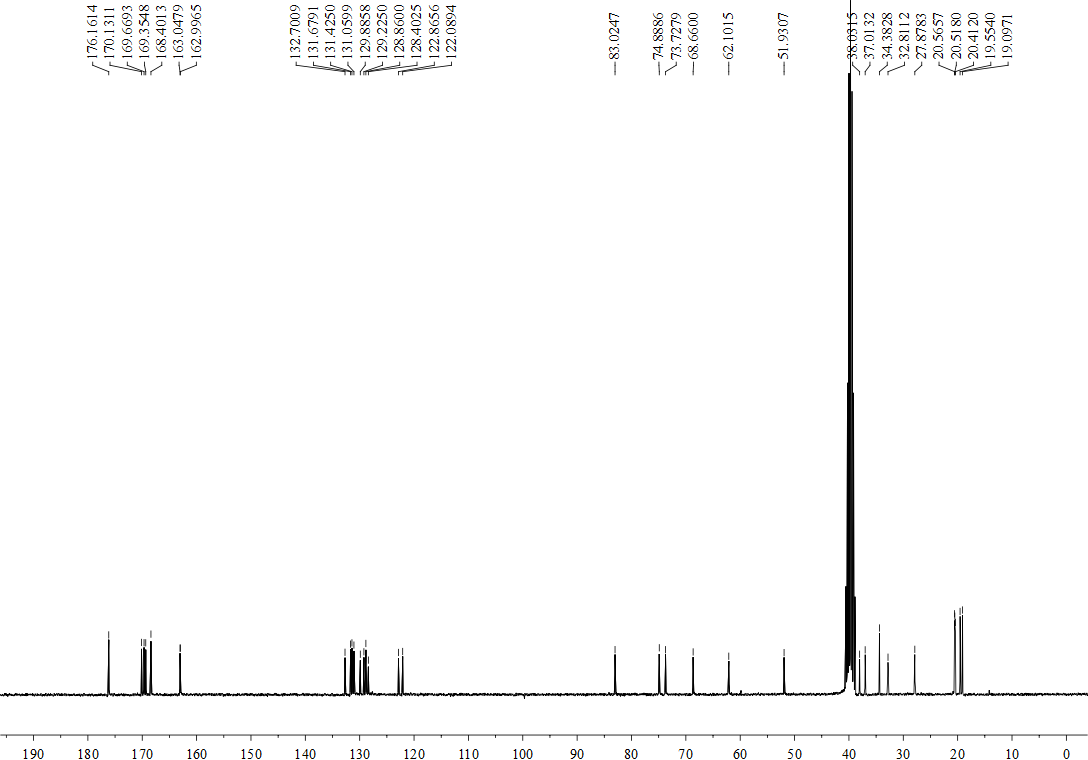


**^1^H NMR spectrum of 15o (300 MHz, DMSO-*d_6_*)**


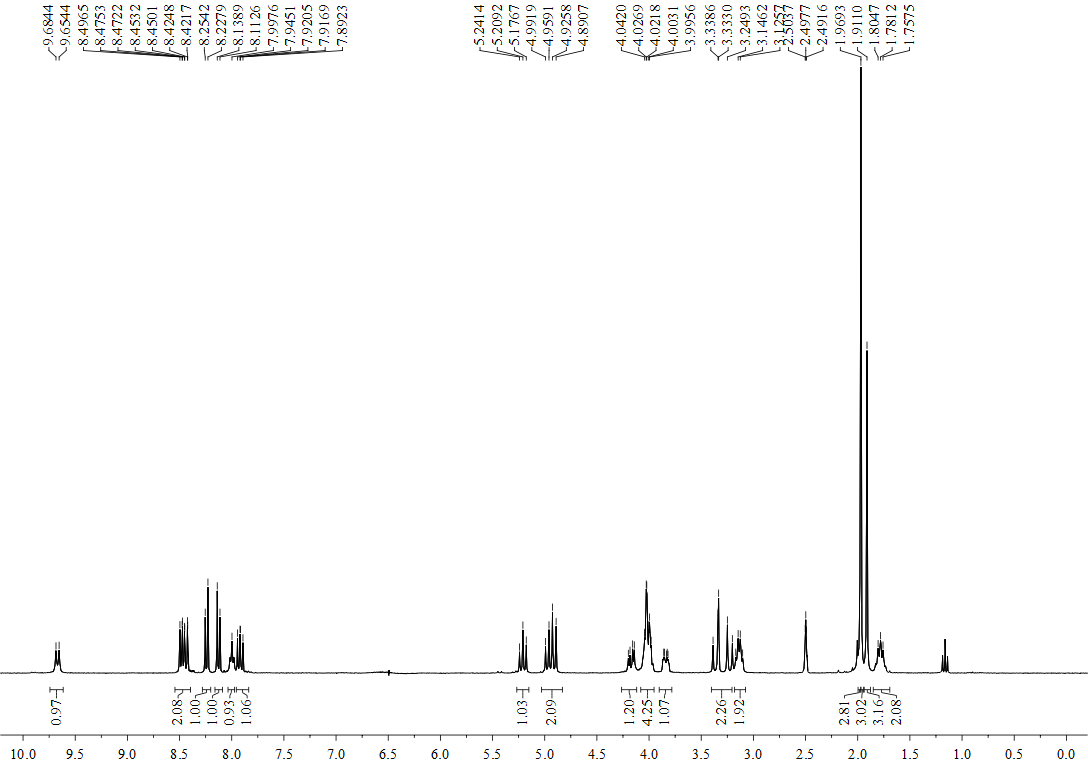


**^13^C NMR spectrum of 15o (300 MHz, DMSO-*d_6_*)**


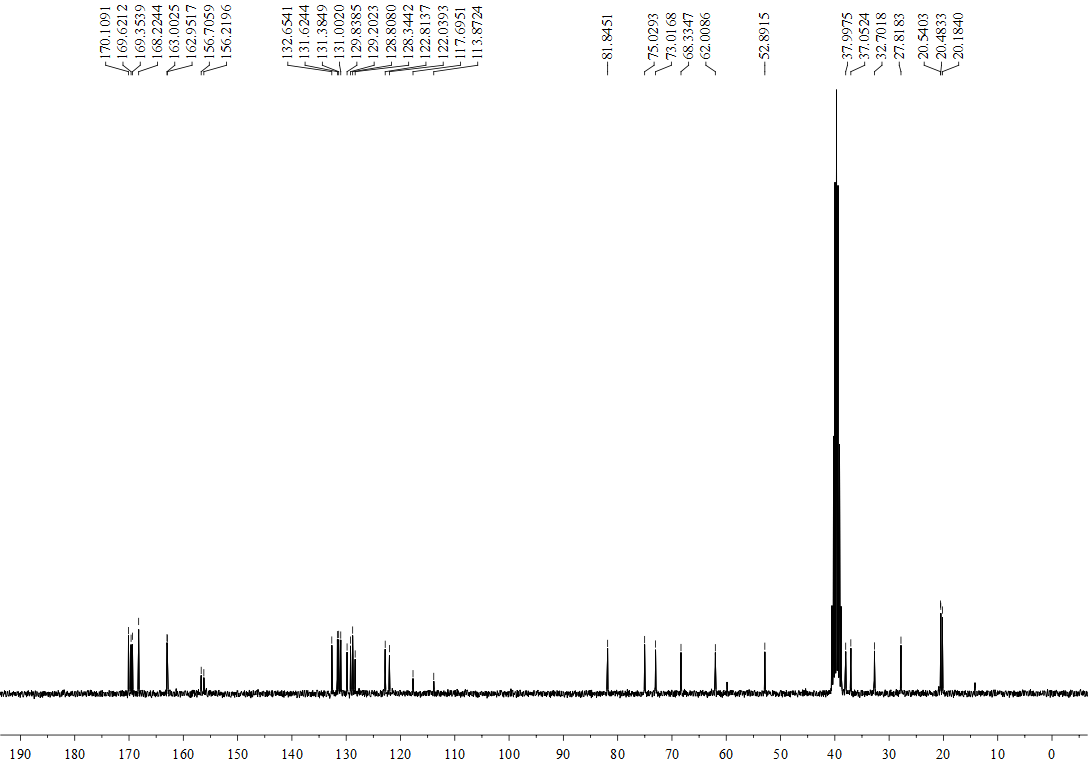


**^1^H NMR spectrum of 15p (300 MHz, DMSO-*d_6_*)**


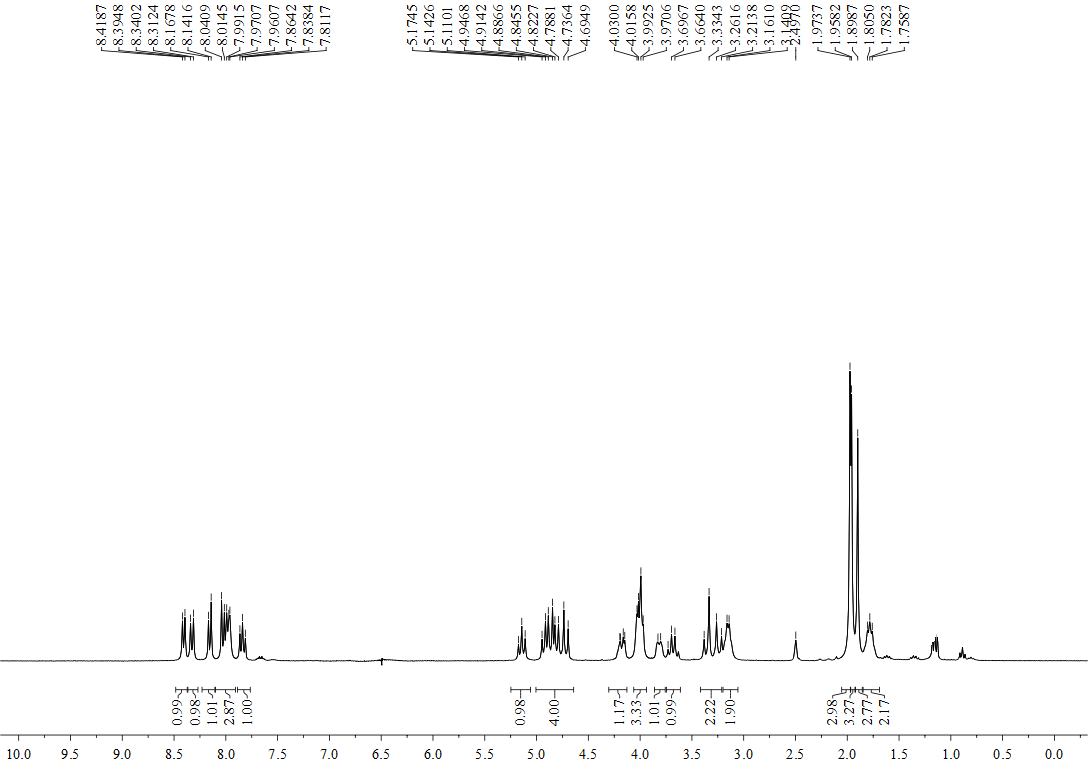


**^13^C NMR spectrum of 15p (300 MHz, DMSO-*d_6_*)**


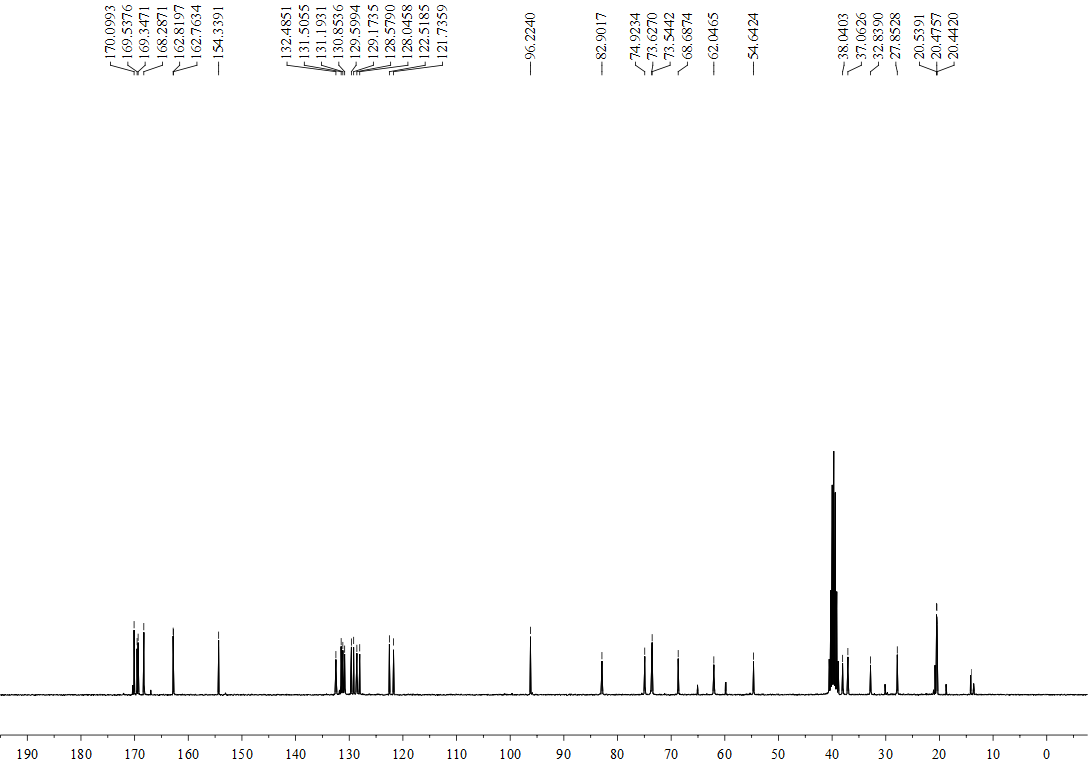


**^1^H NMR spectrum of 16a (300 MHz, DMSO-*d_6_*)**


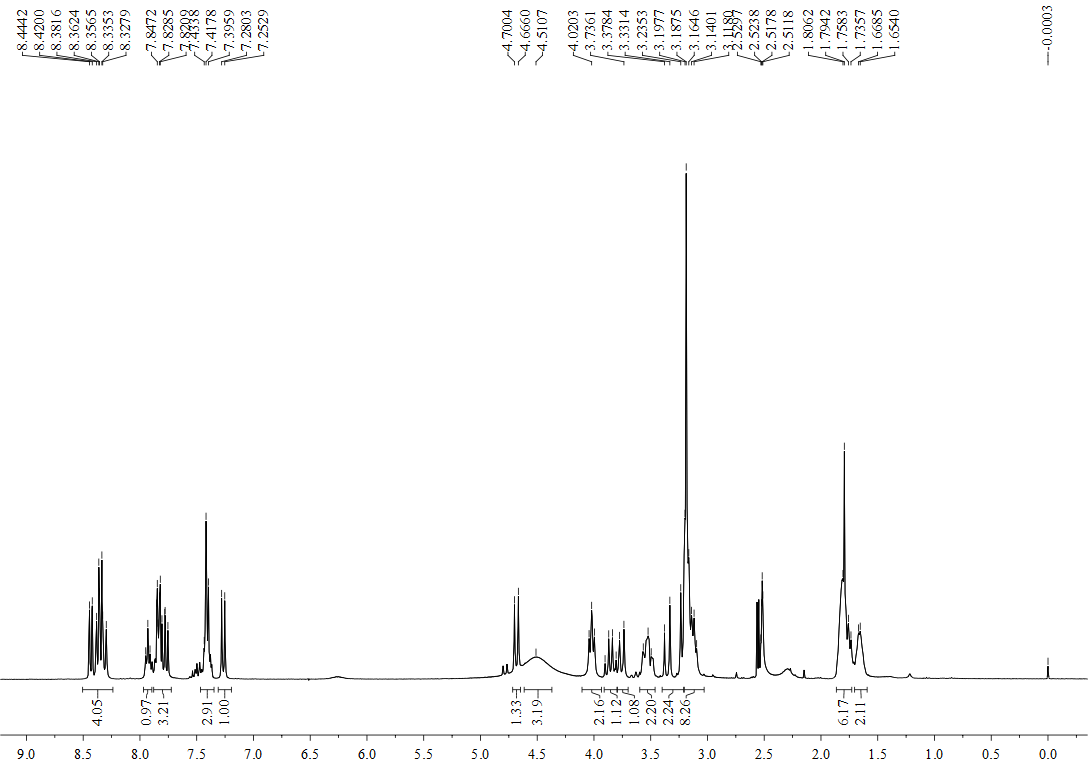


**^13^C NMR spectrum of 16a (300 MHz, DMSO-*d_6_*)**


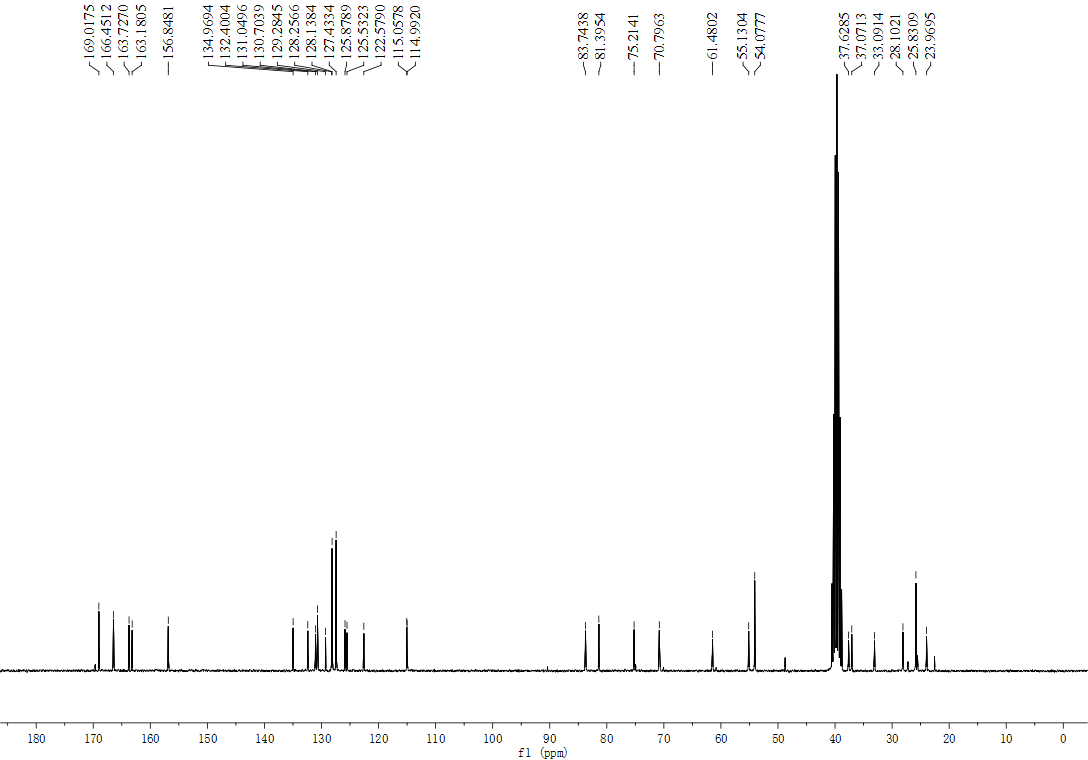


**^1^H NMR spectrum of 16b (300 MHz, DMSO-*d_6_*)**


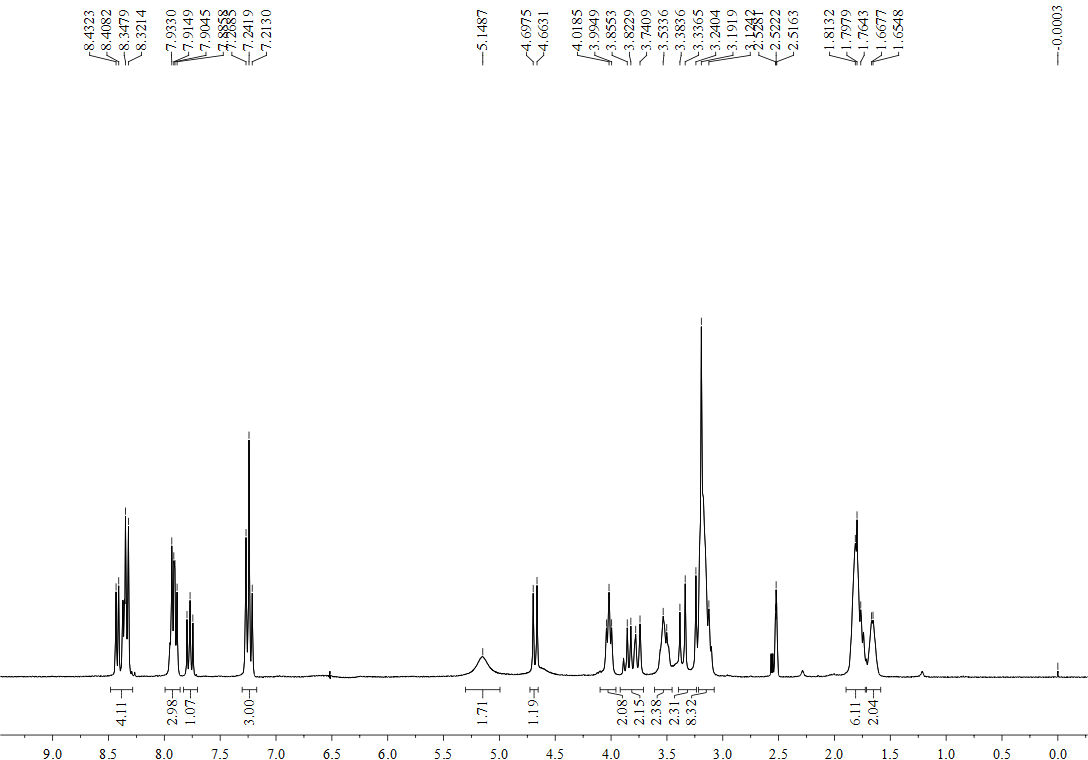


**^13^C NMR spectrum of 16b (300 MHz, DMSO-*d_6_*)**


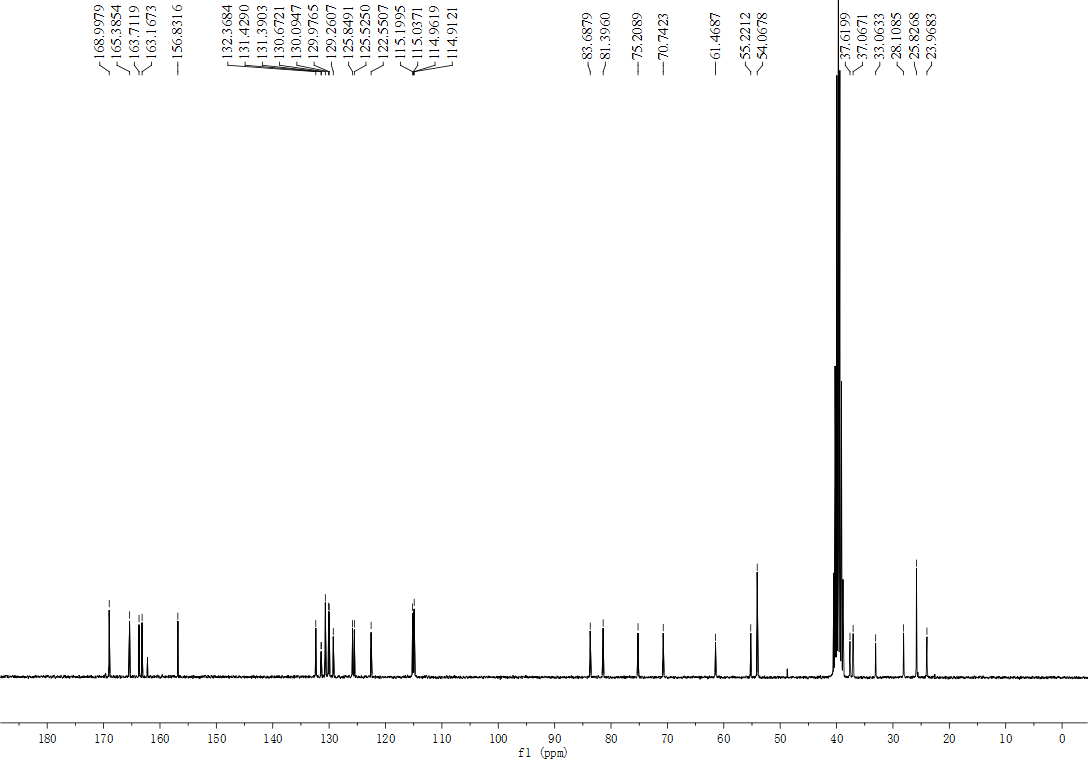


**^1^H NMR spectrum of 16c (300 MHz, DMSO-*d_6_*)**


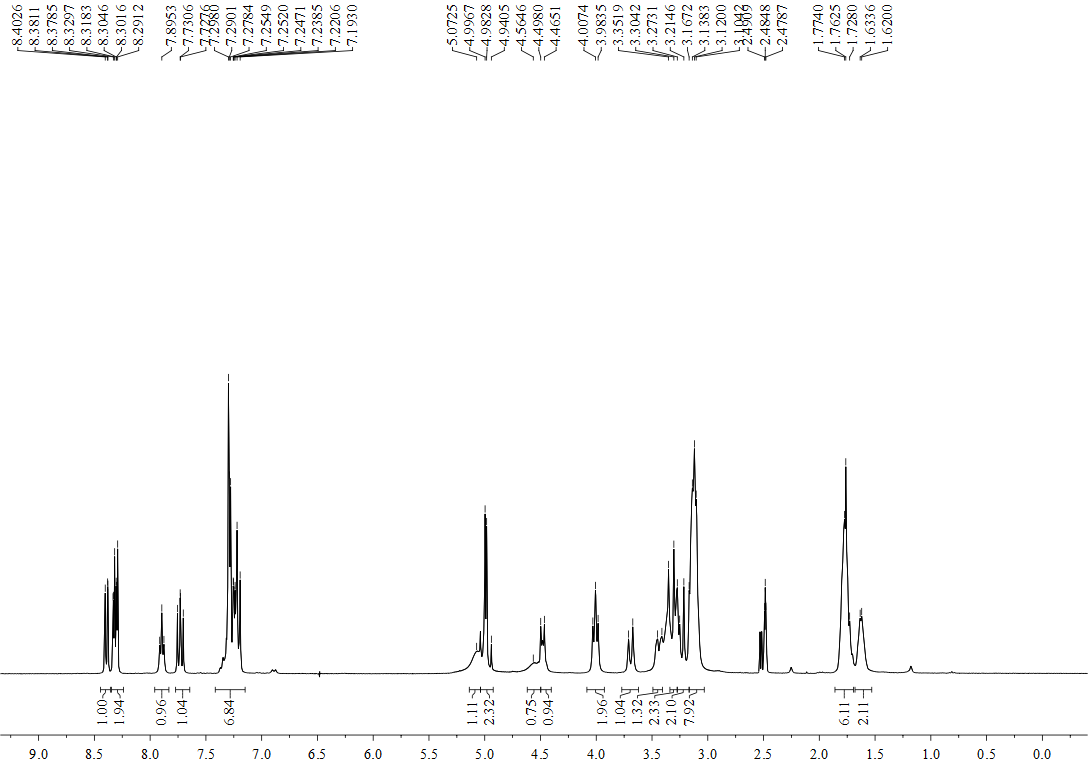


**^13^C NMR spectrum of 16c (300 MHz, DMSO-*d_6_*)**


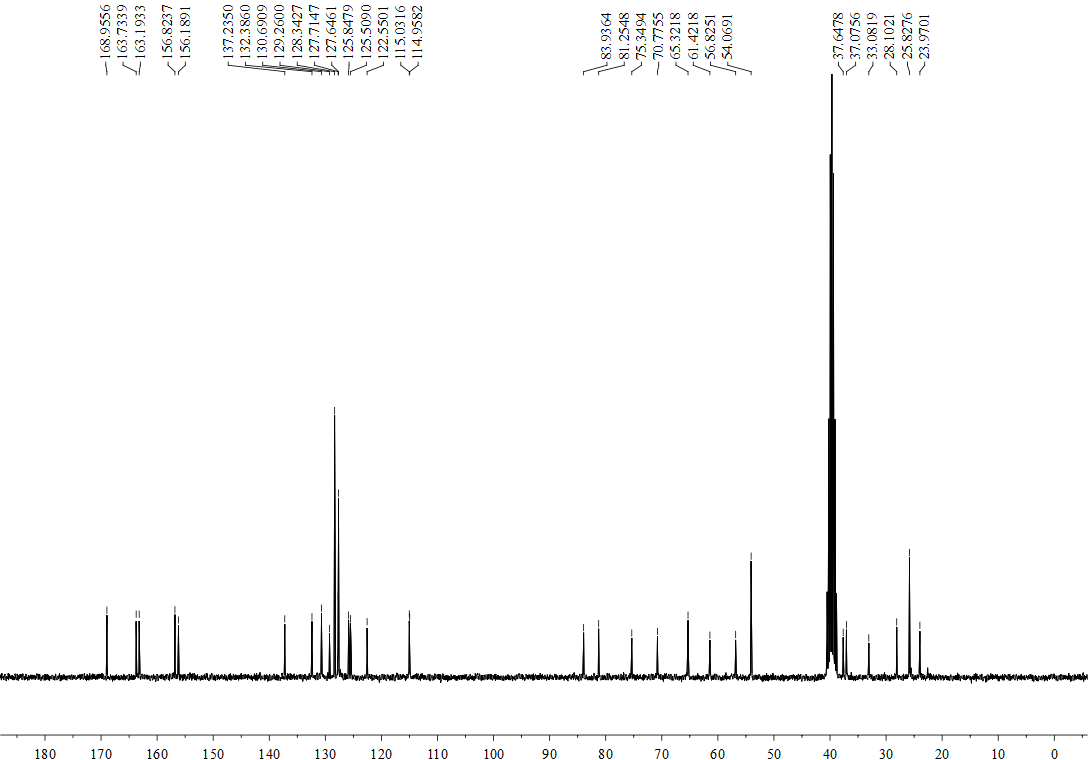


**^1^H NMR spectrum of 16d (300 MHz, DMSO-*d_6_*)**


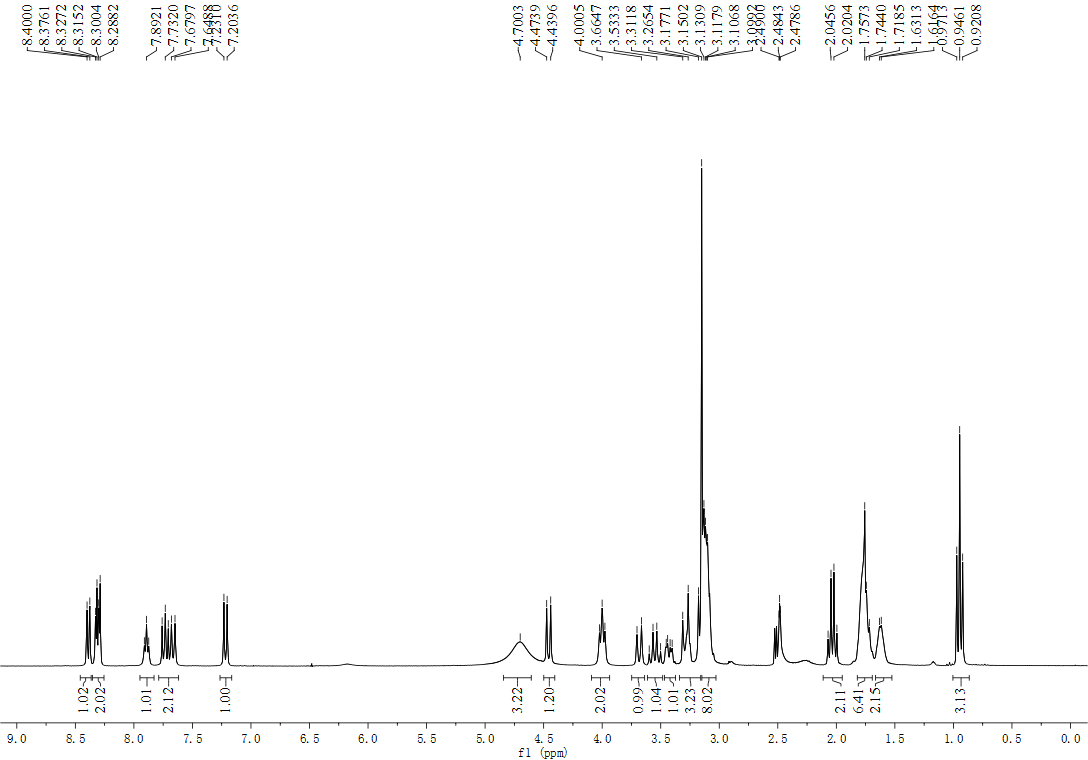


**^13^C NMR spectrum of 16d (300 MHz, DMSO-*d_6_*)**


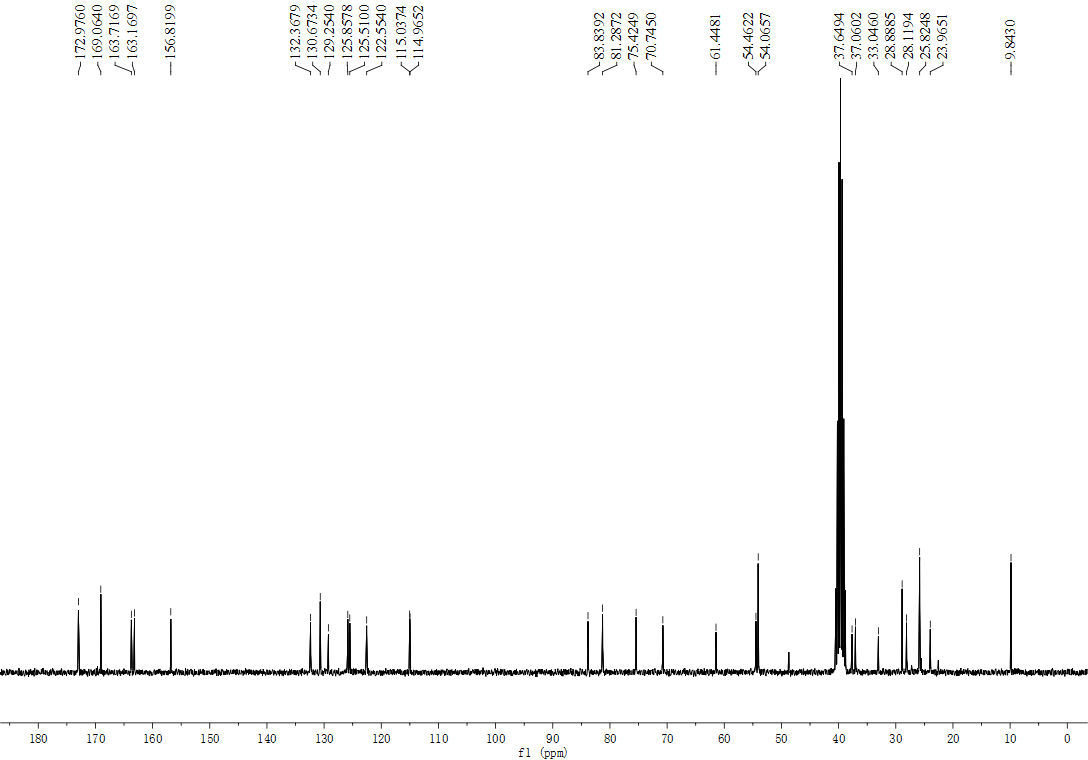


**^1^H NMR spectrum of 16e (300 MHz, DMSO-*d_6_*)**


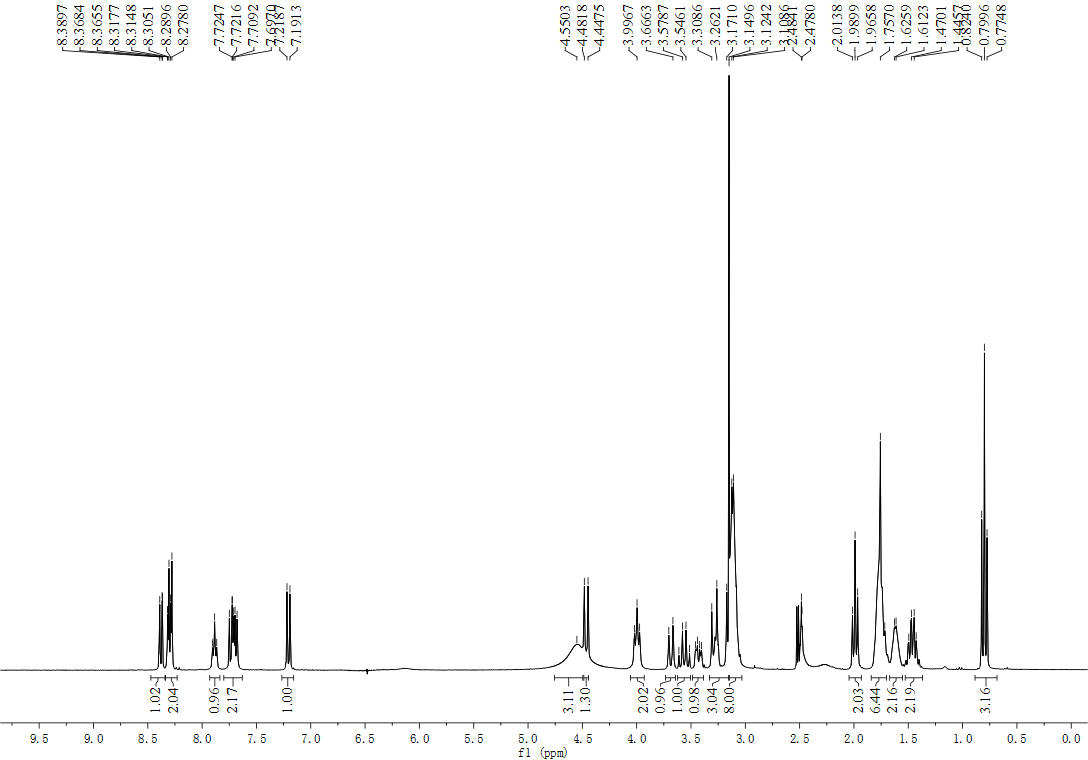


**^13^C NMR spectrum of 16e (300 MHz, DMSO-*d_6_*)**


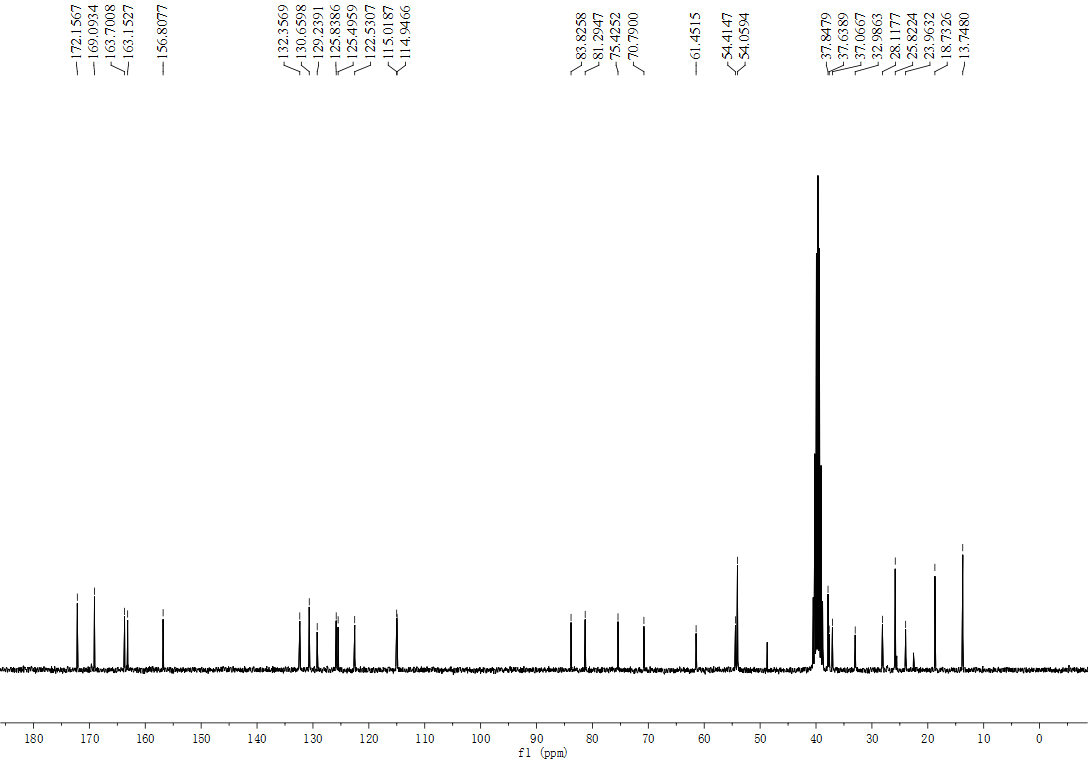


**^1^H NMR spectrum of 16f (300 MHz, DMSO-*d_6_*)**


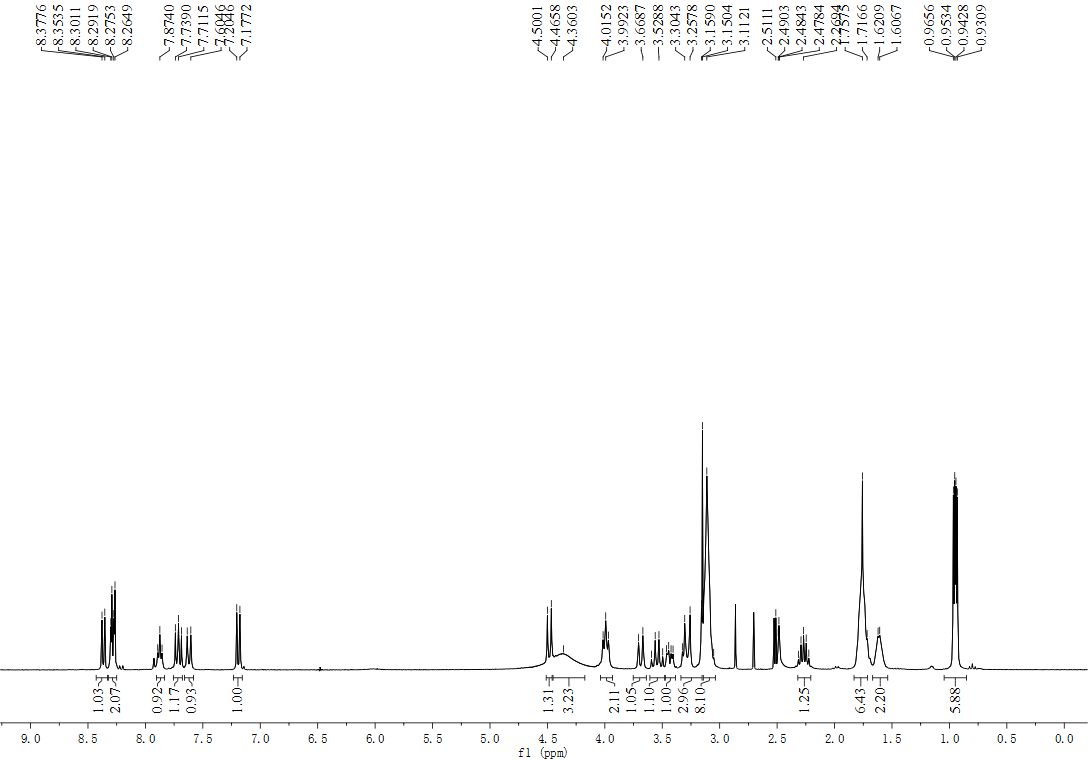


**^13^C NMR spectrum of 16f (300 MHz, DMSO-*d_6_*)**


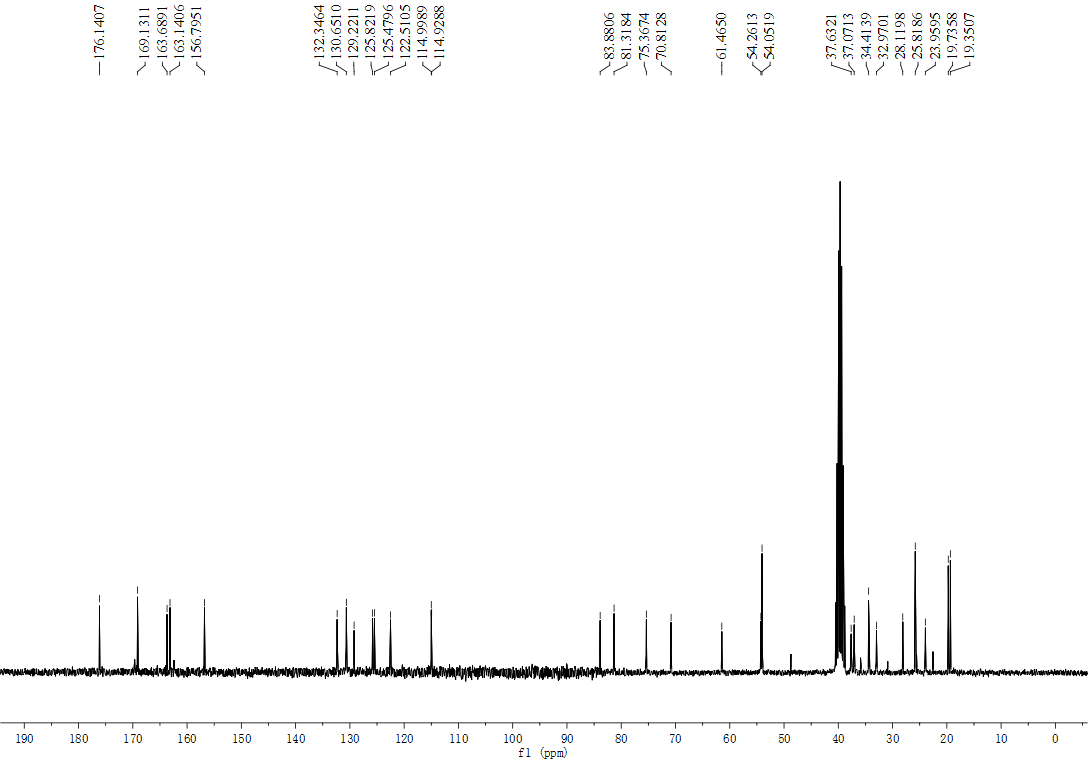


**^1^H NMR spectrum of 16g (300 MHz, DMSO-*d_6_*)**


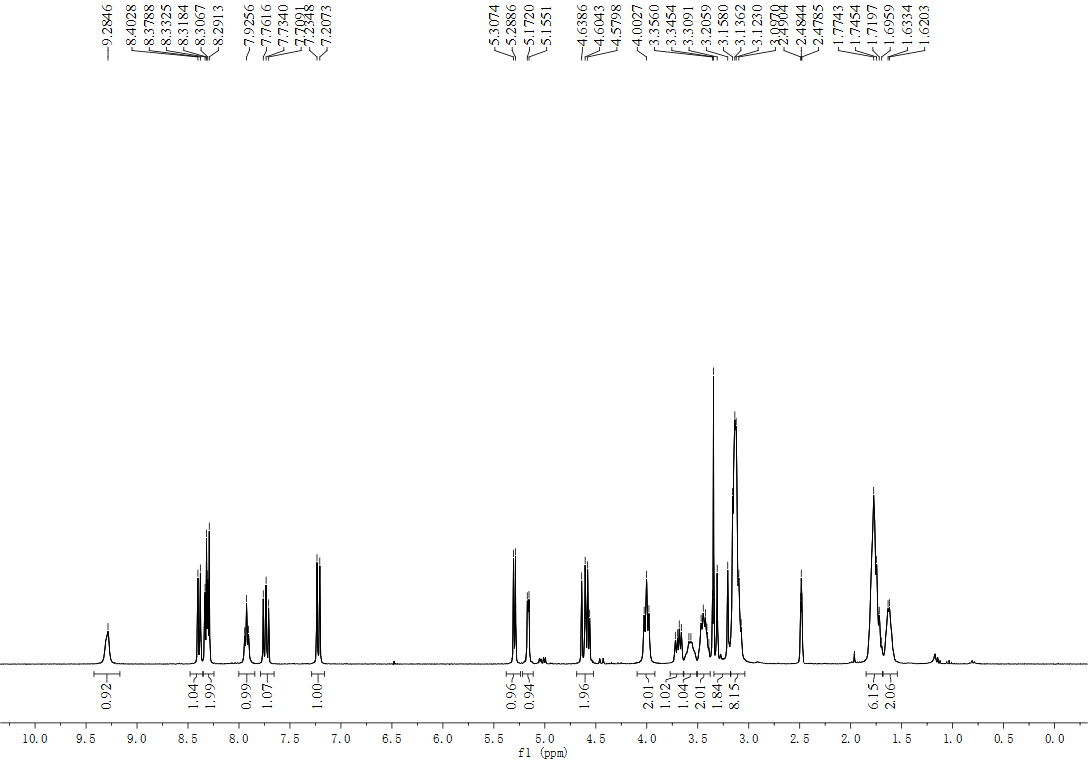


**^13^C NMR spectrum of 16g (300 MHz, DMSO-*d_6_*)**


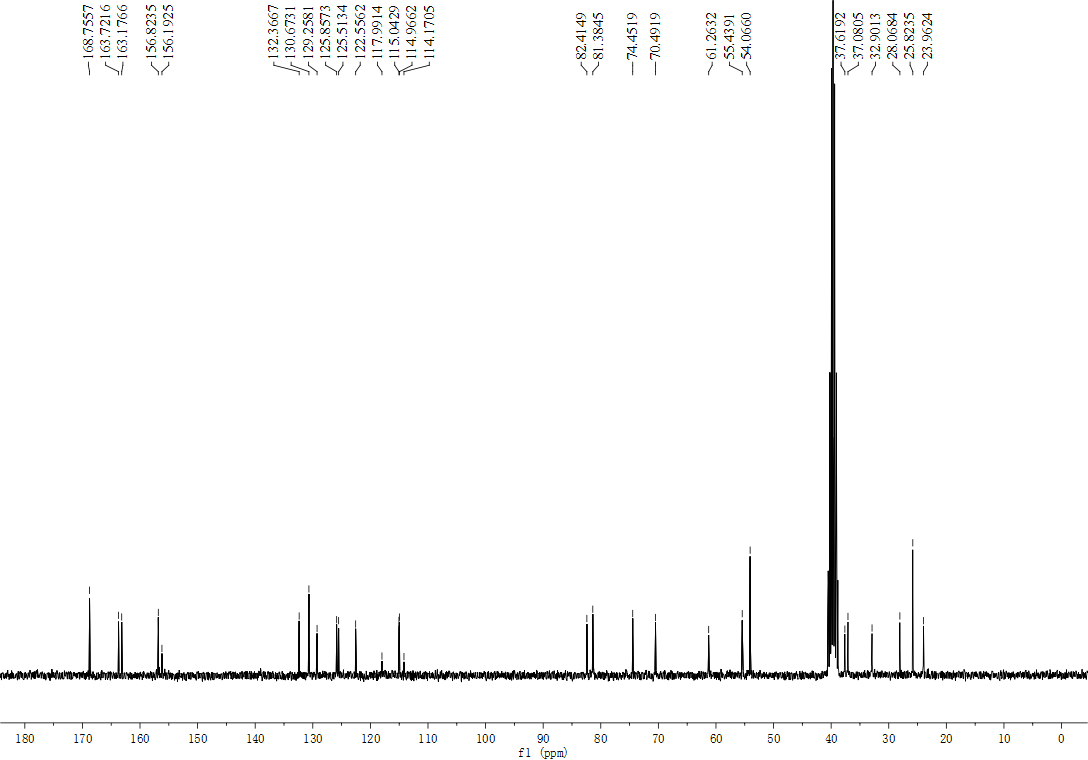


**^1^H NMR spectrum of 16h (300 MHz, DMSO-*d_6_*)**


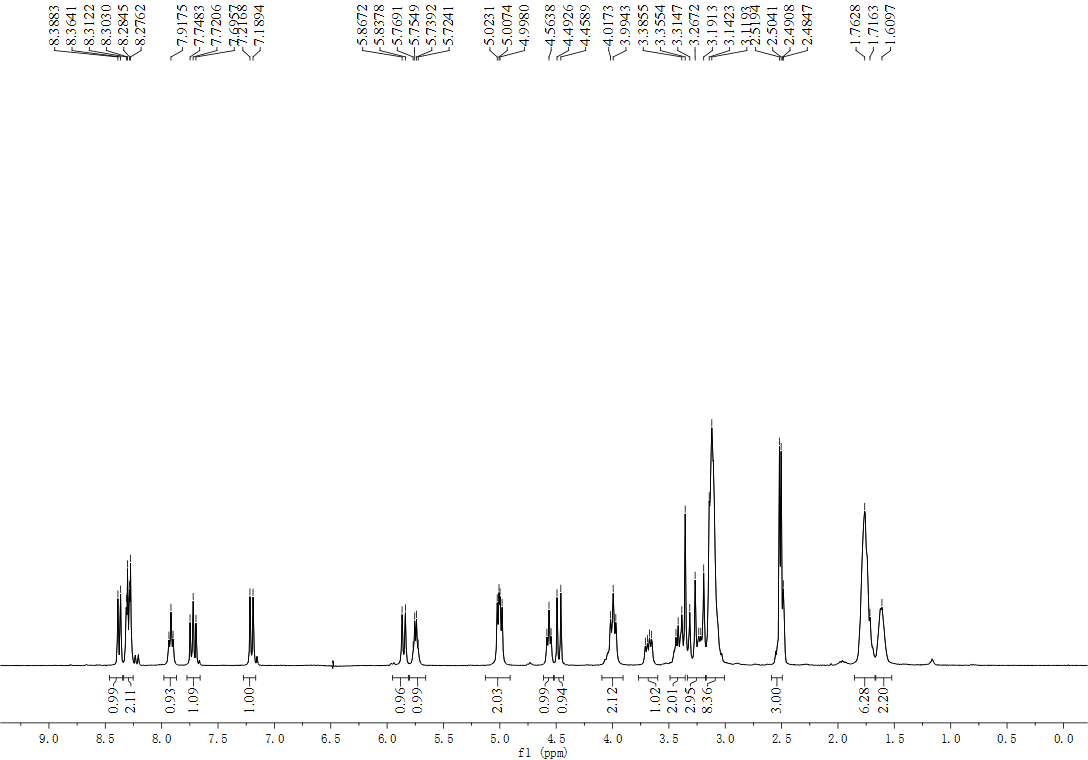


**^13^C NMR spectrum of 16h (300 MHz, DMSO-*d_6_*)**


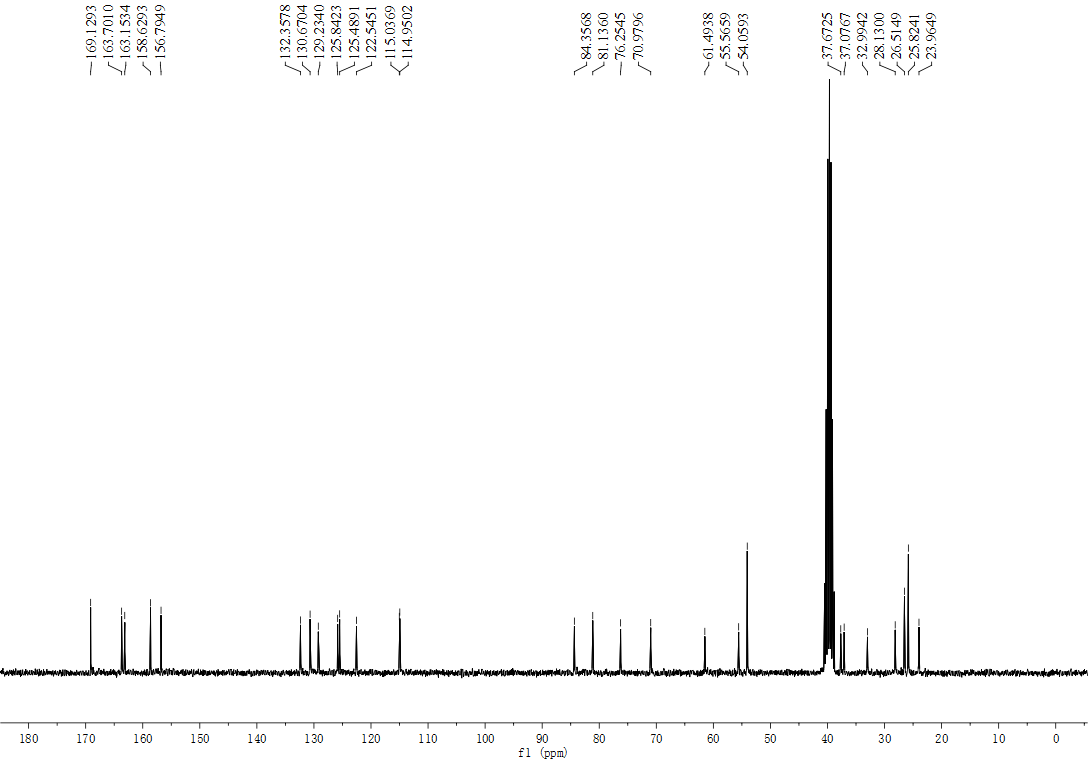


**^1^H NMR spectrum of 16j (300 MHz, DMSO-*d_6_*)**


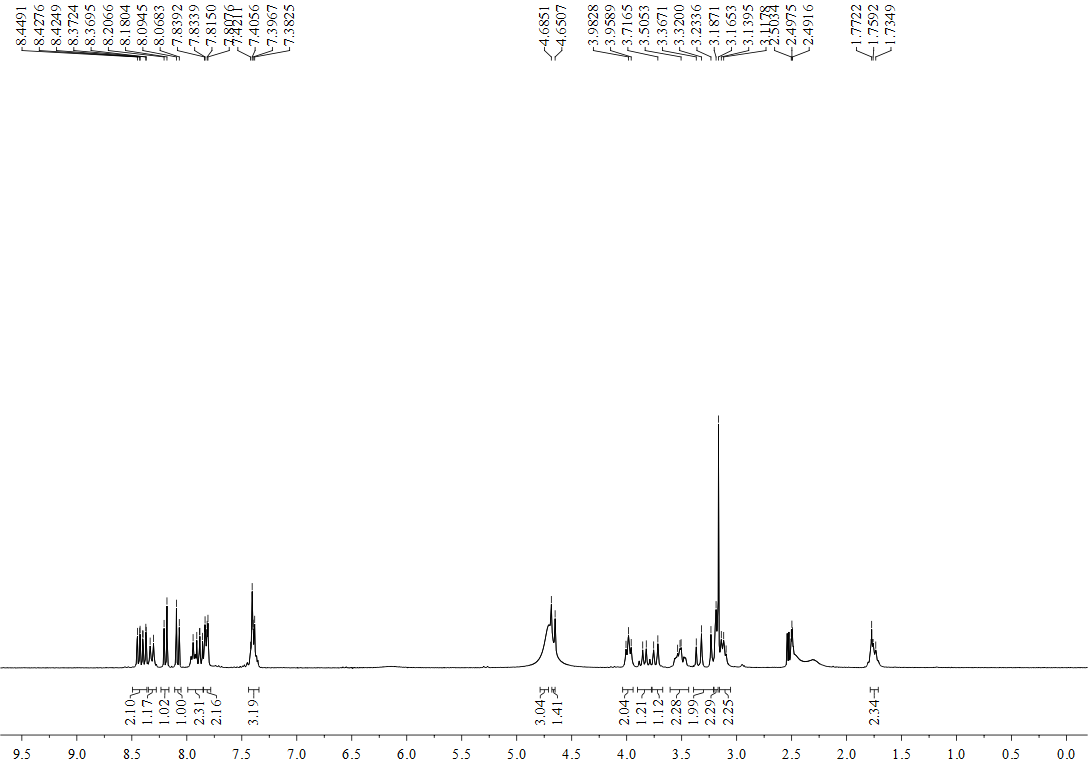


**^13^C NMR spectrum of 16j (300 MHz, DMSO-*d_6_*)**


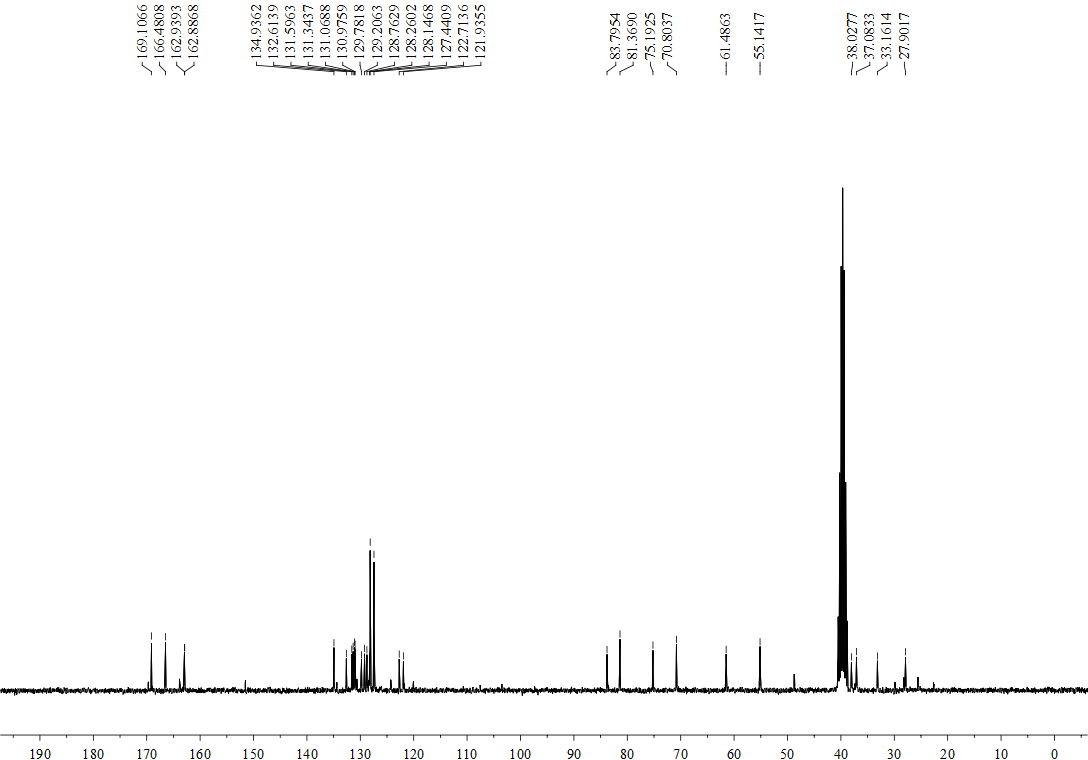


**^1^H NMR spectrum of 16k (300 MHz, DMSO-*d_6_*)**


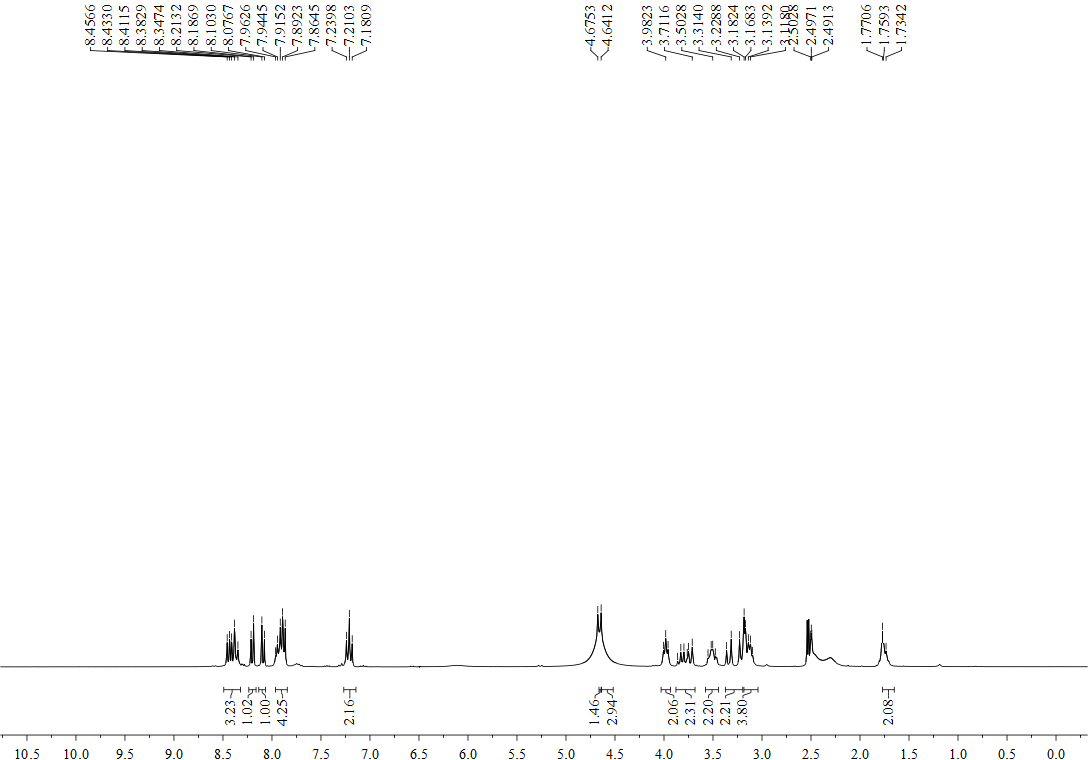


**^13^C NMR spectrum of 16k (300 MHz, DMSO-*d_6_*)**


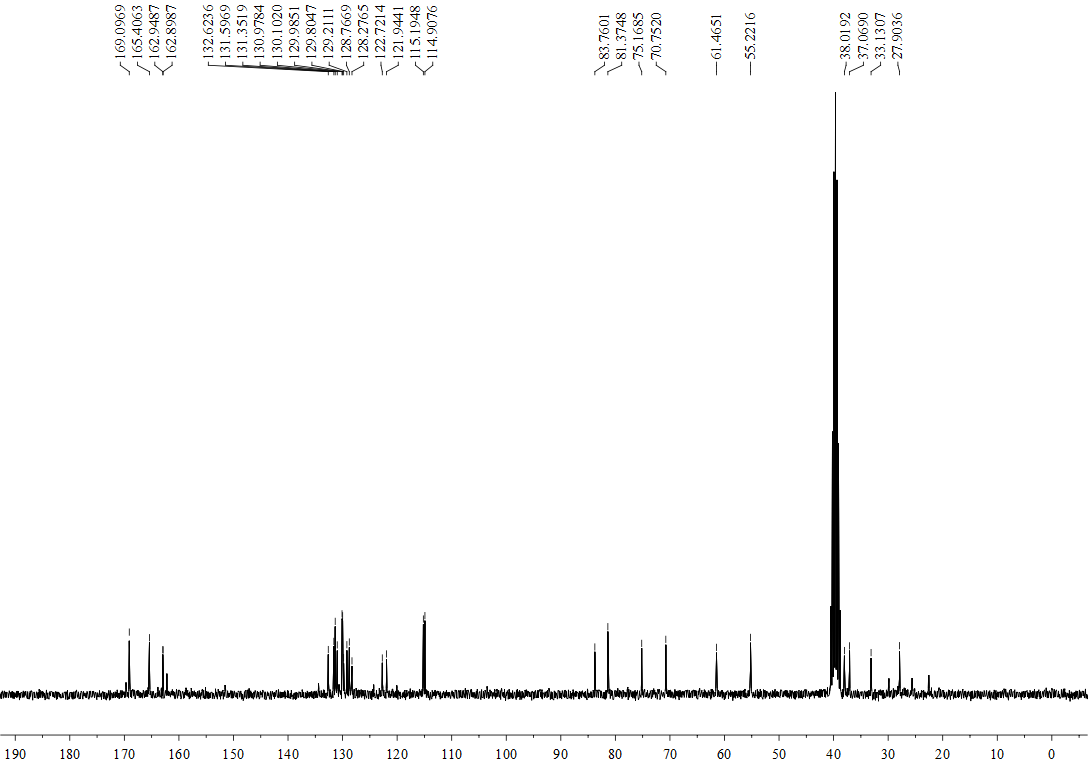


**^1^H NMR spectrum of 16l (300 MHz, DMSO-*d_6_*)**


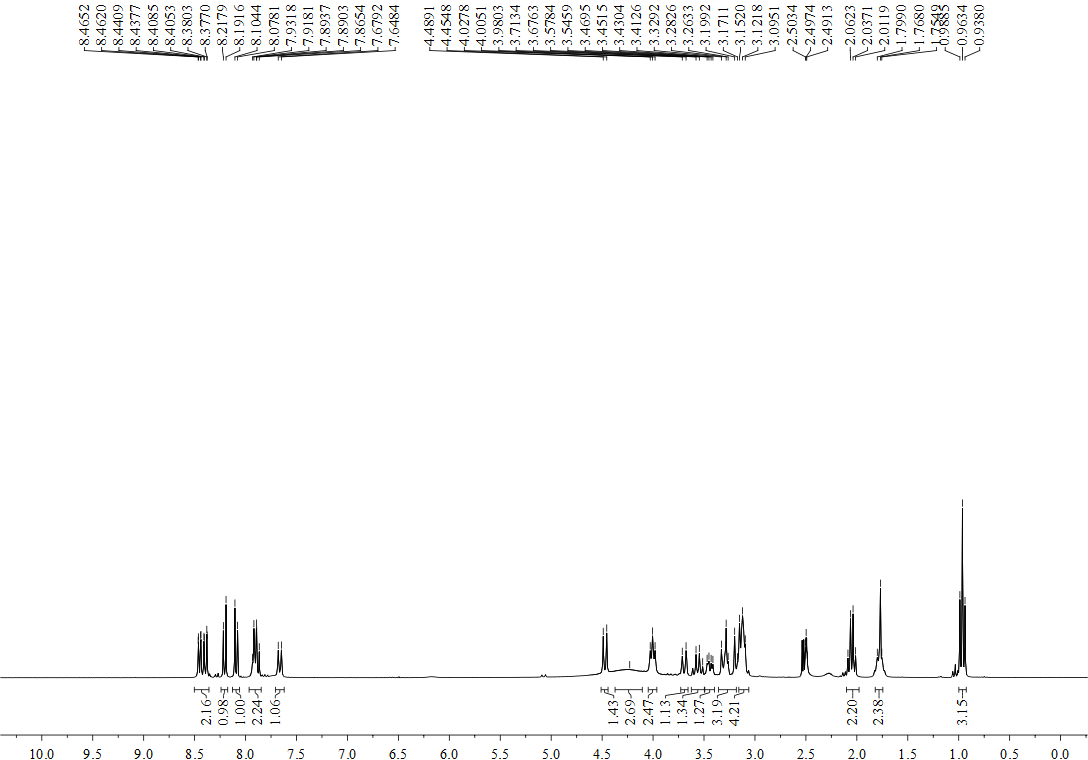


**^13^C NMR spectrum of 16l (300 MHz, DMSO-*d_6_*)**


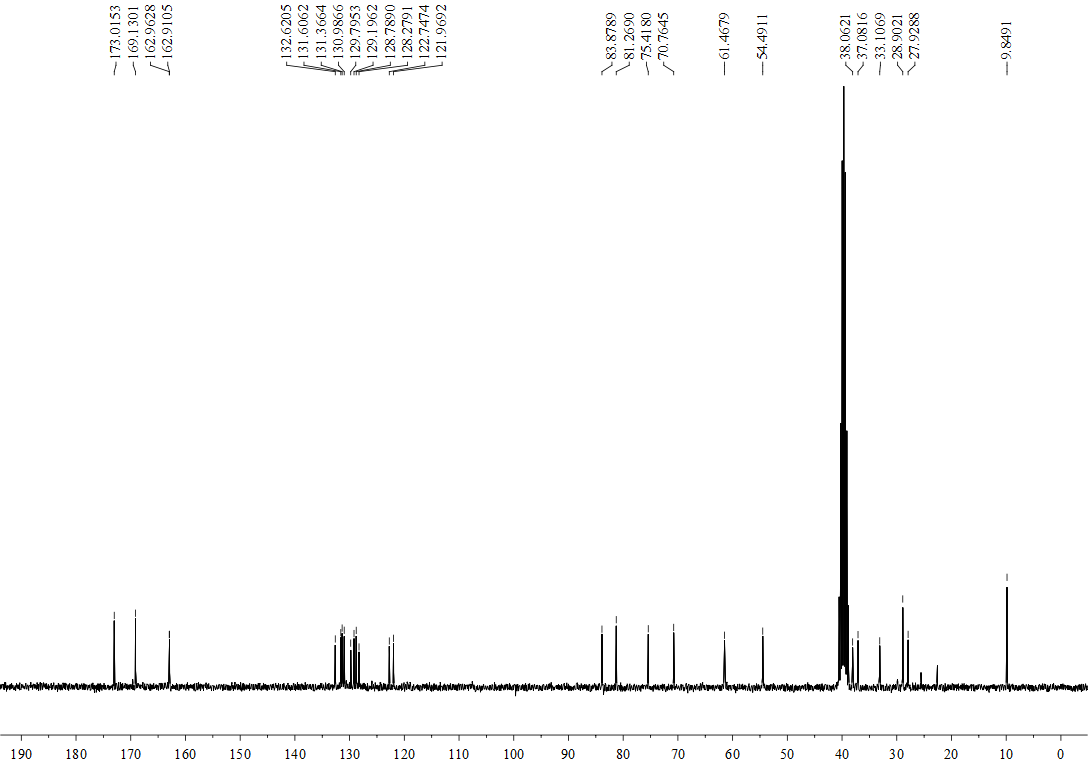


**^1^H NMR spectrum of 16m (300 MHz, DMSO-*d_6_*)**


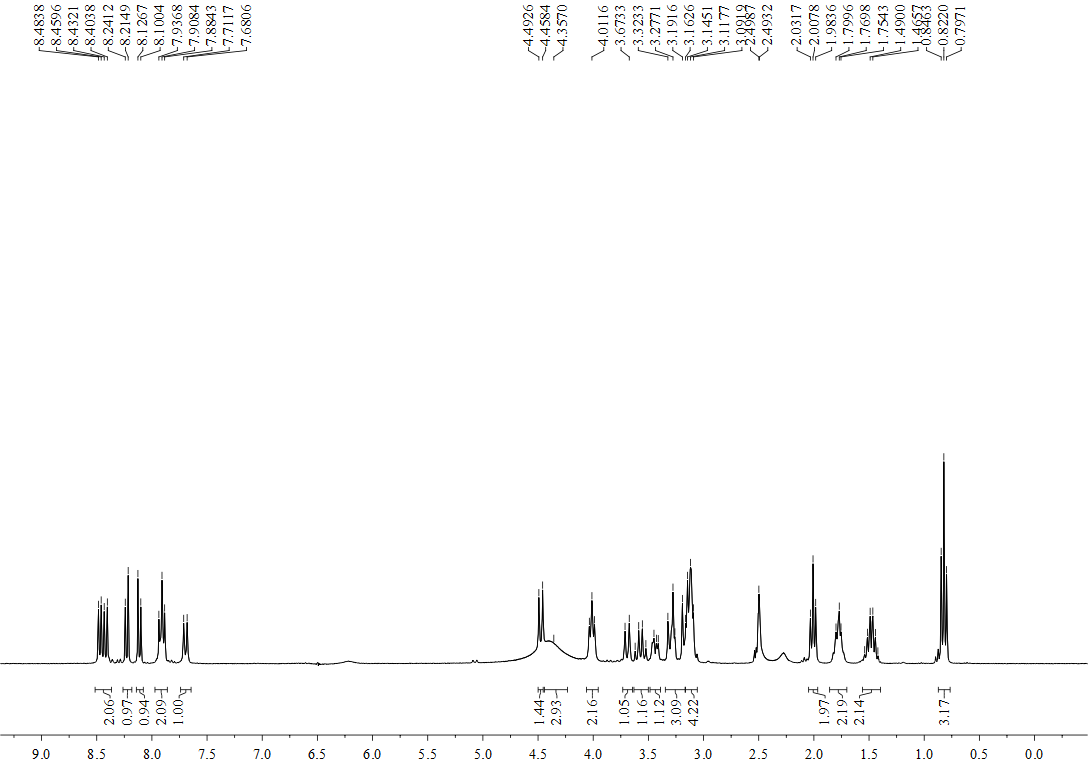


**^13^C NMR spectrum of 16m (300 MHz, DMSO-*d_6_*)**


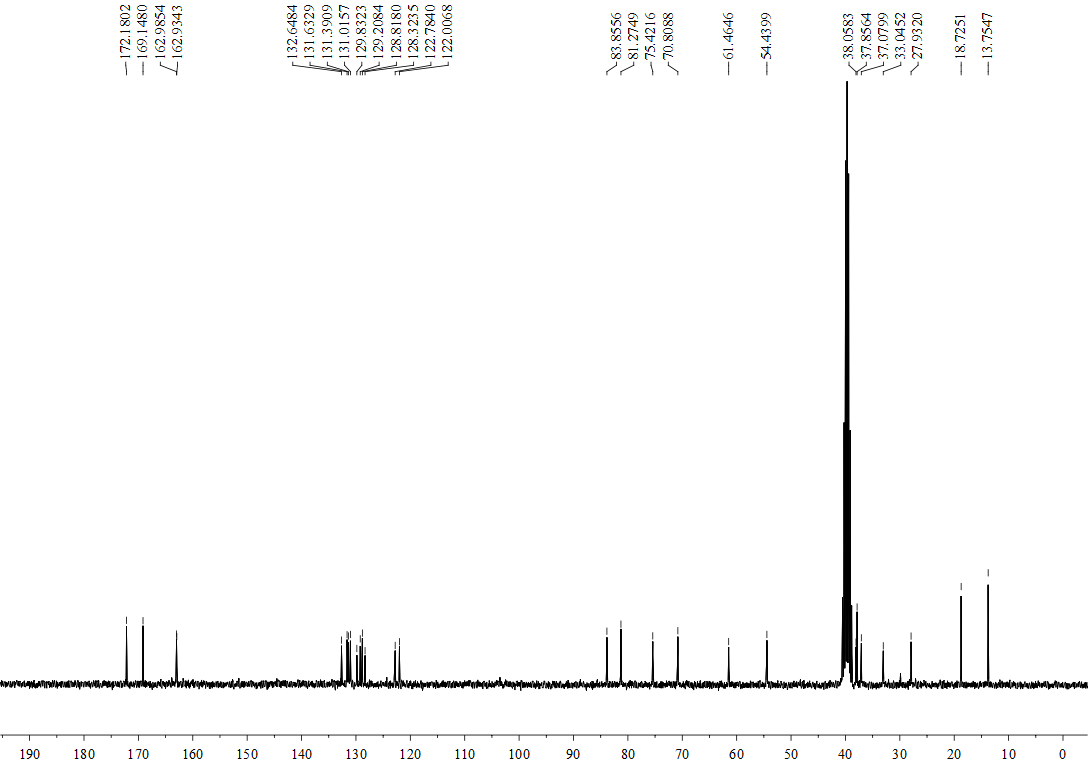


**^1^H NMR spectrum of 16n (300 MHz, DMSO-*d_6_*)**


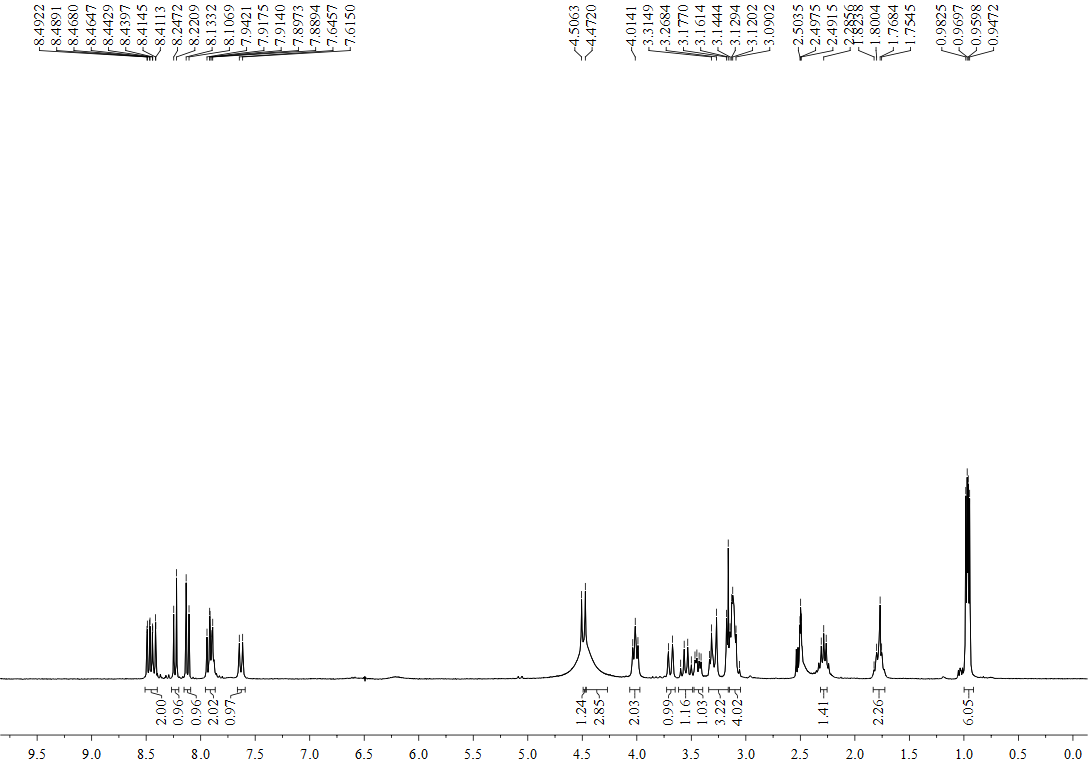


**^13^C NMR spectrum of 16n (300 MHz, DMSO-*d_6_*)**


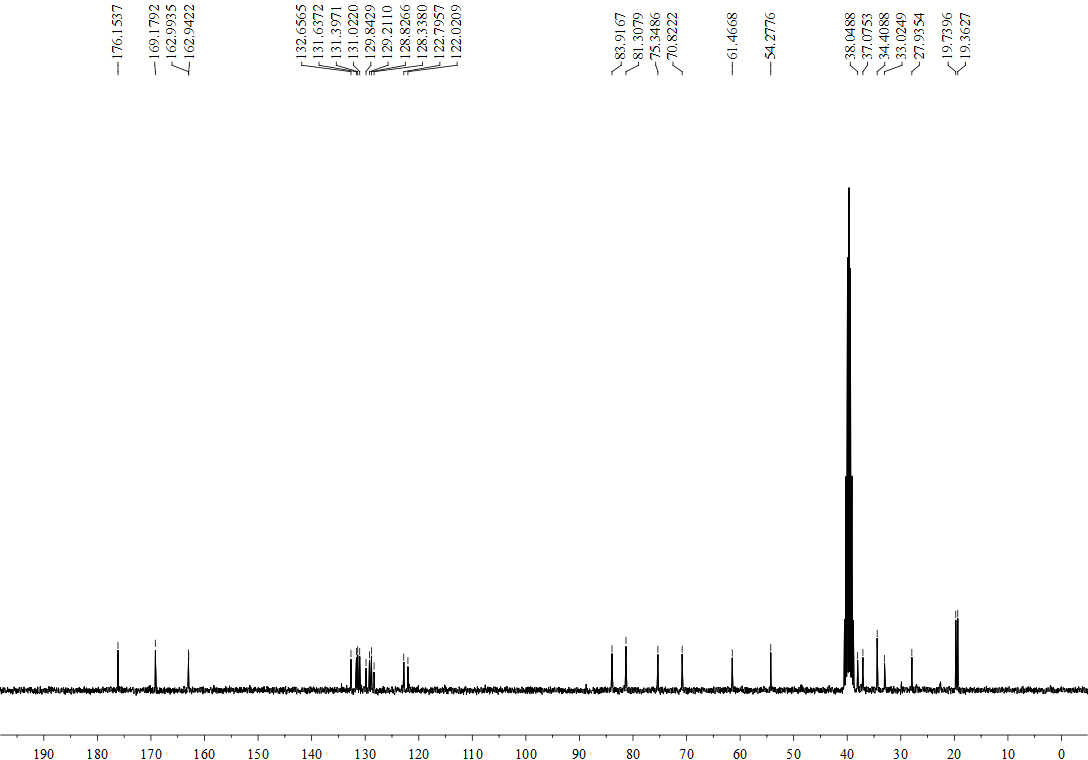


**^1^H NMR spectrum of 16o (300 MHz, DMSO-*d_6_*)**


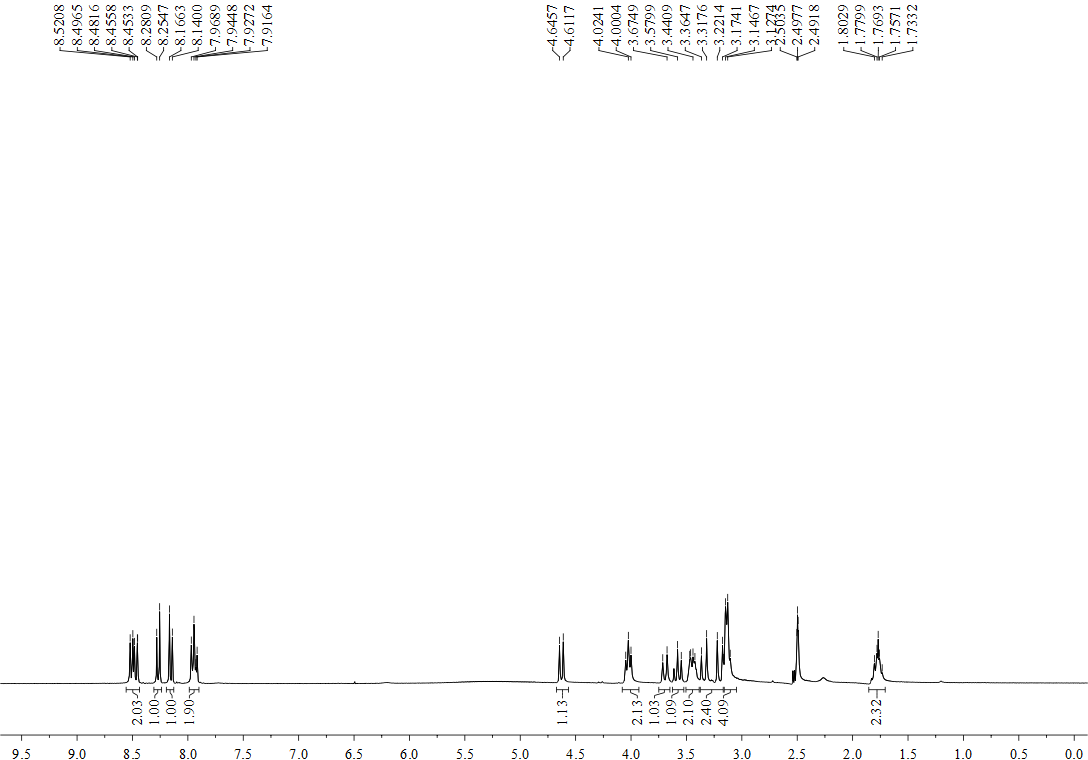


**^13^C NMR spectrum of 16o (300 MHz, DMSO-*d_6_*)**


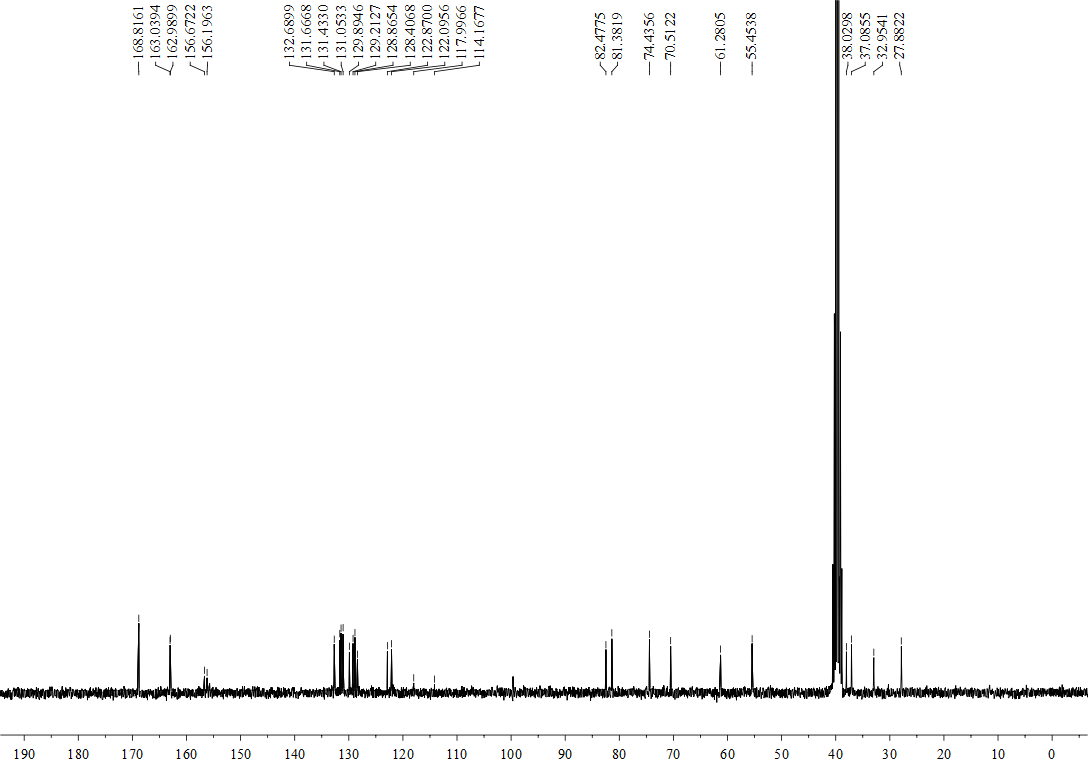


**^1^H NMR spectrum of 17a (300 MHz, DMSO-*d_6_*)**


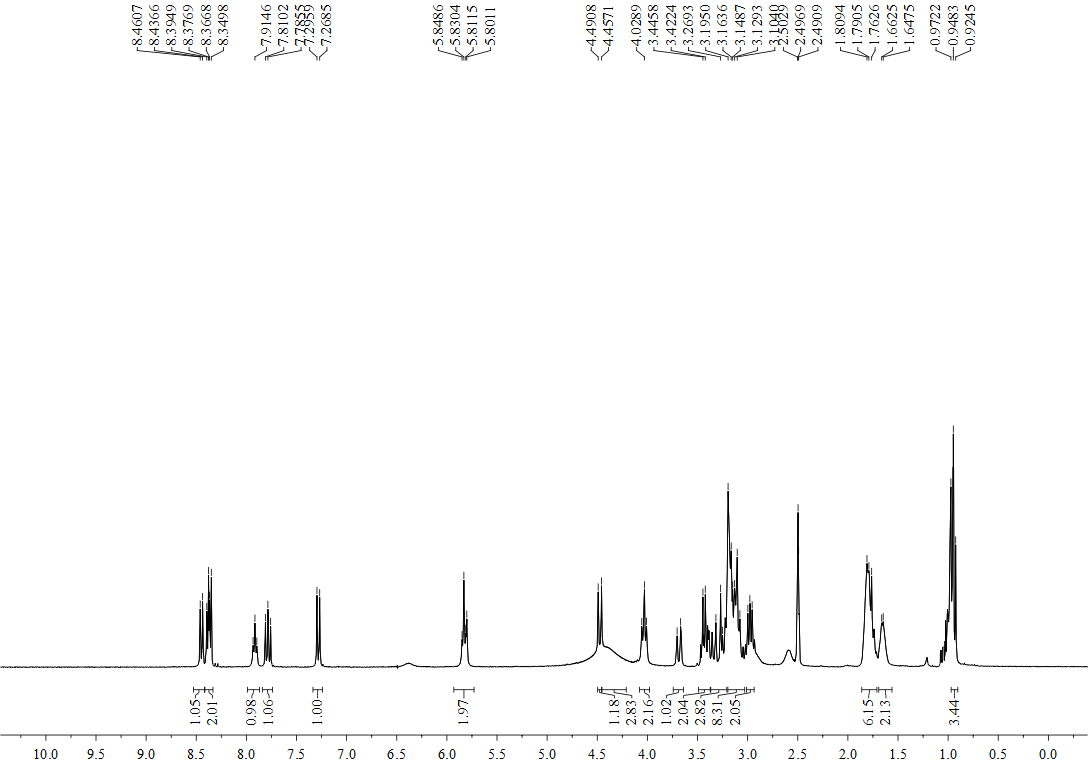


**^13^C NMR spectrum of 17a (300 MHz, DMSO-*d_6_*)**


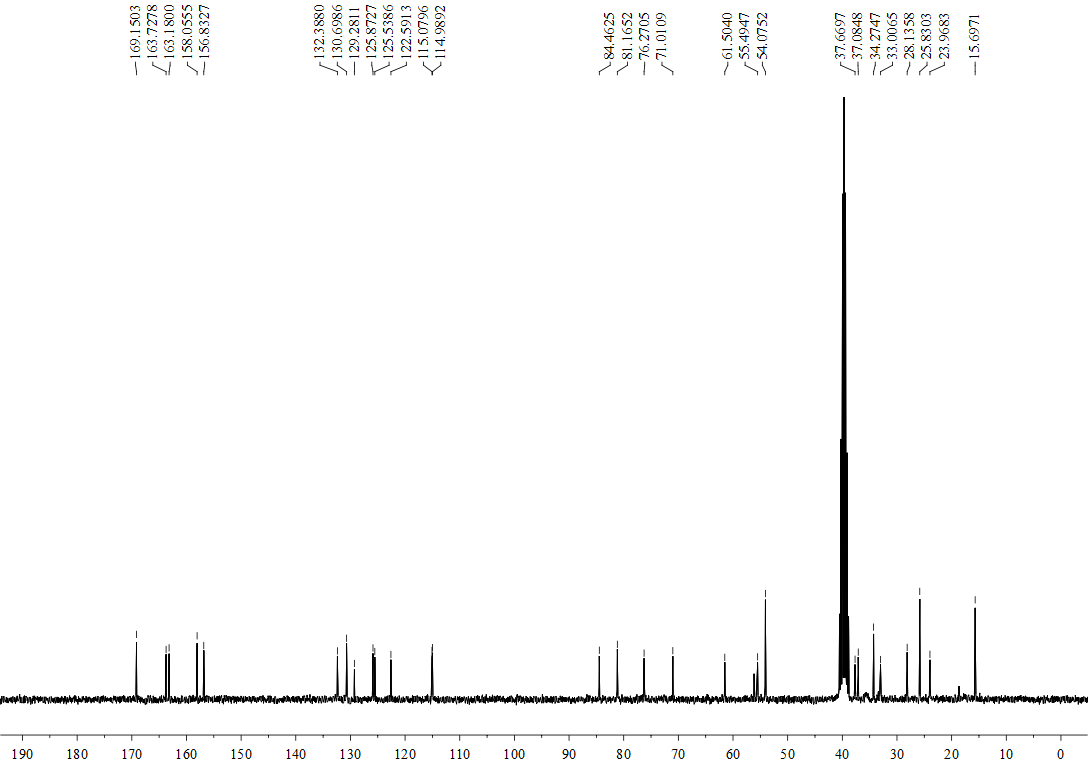


**^1^H NMR spectrum of 17b (300 MHz, DMSO-*d_6_*)**


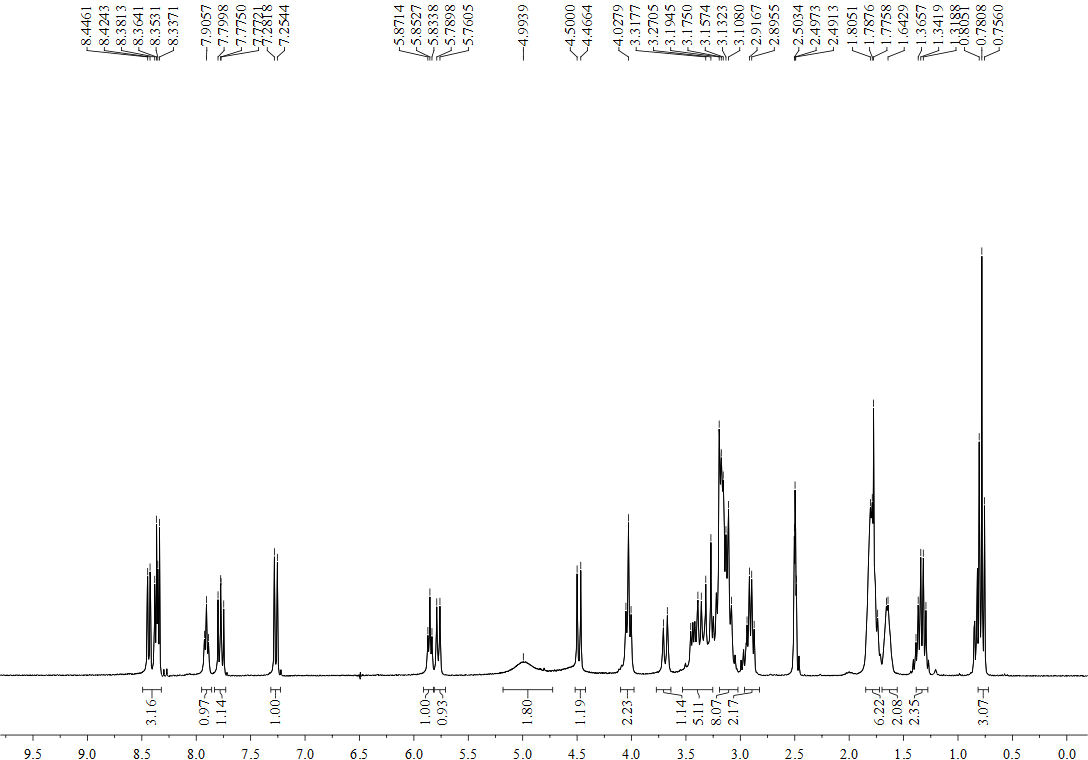


**^13^C NMR spectrum of 17b (300 MHz, DMSO-*d_6_*)**


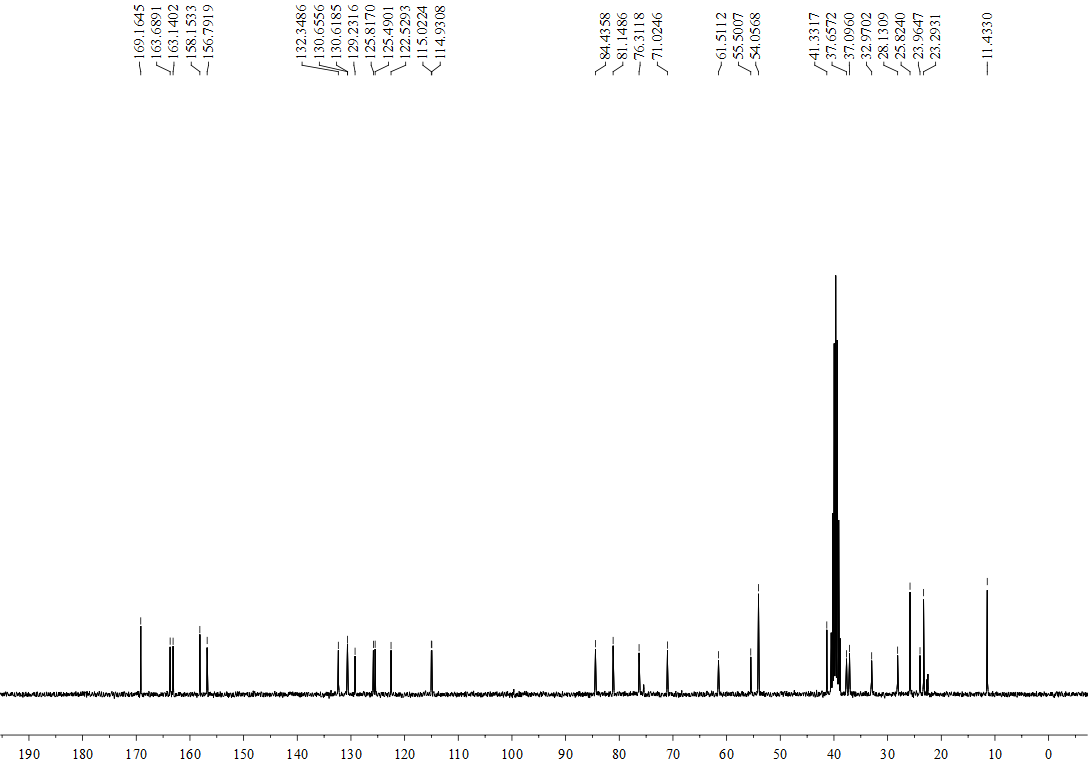


**^1^H NMR spectrum of 17c (300 MHz, DMSO-*d_6_*)**


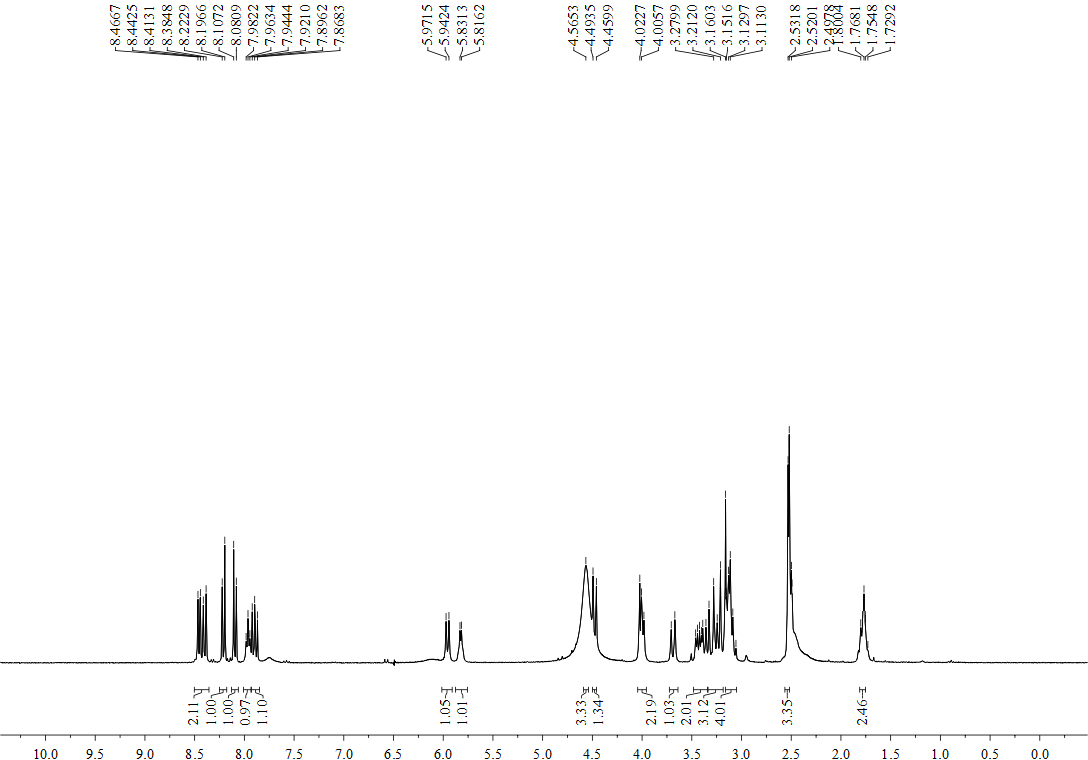


**^13^C NMR spectrum of 17c (300 MHz, DMSO-*d_6_*)**


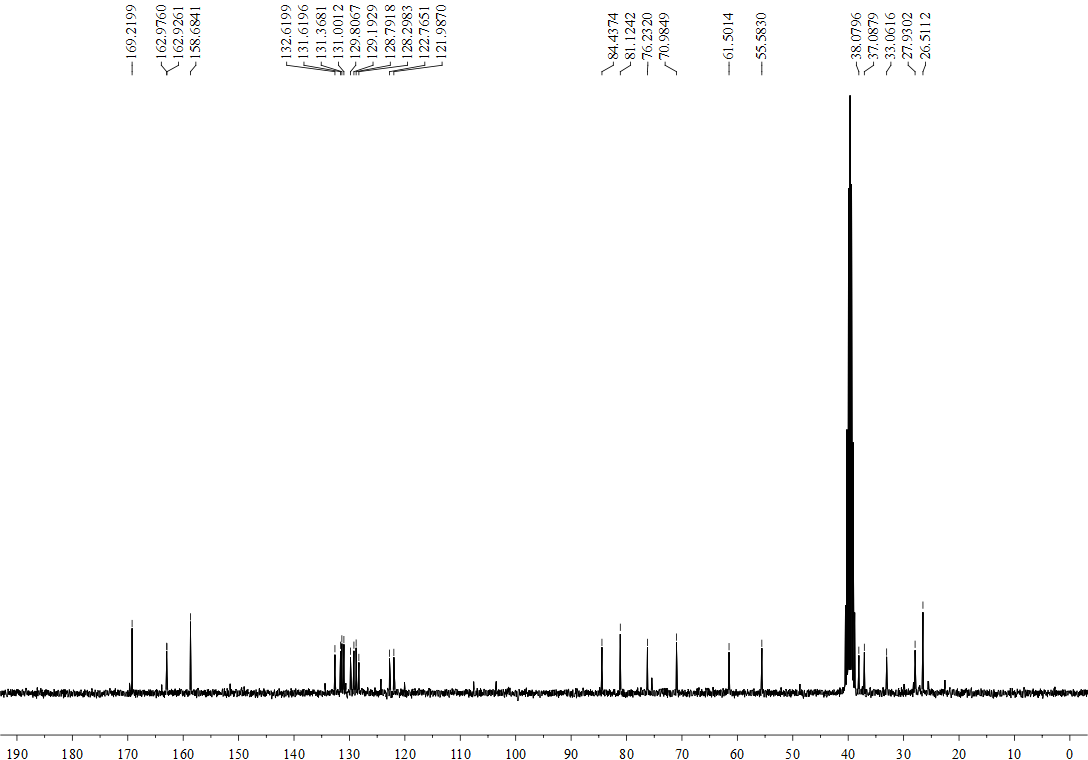


**^1^H NMR spectrum of 17d (300 MHz, DMSO-*d_6_*)**


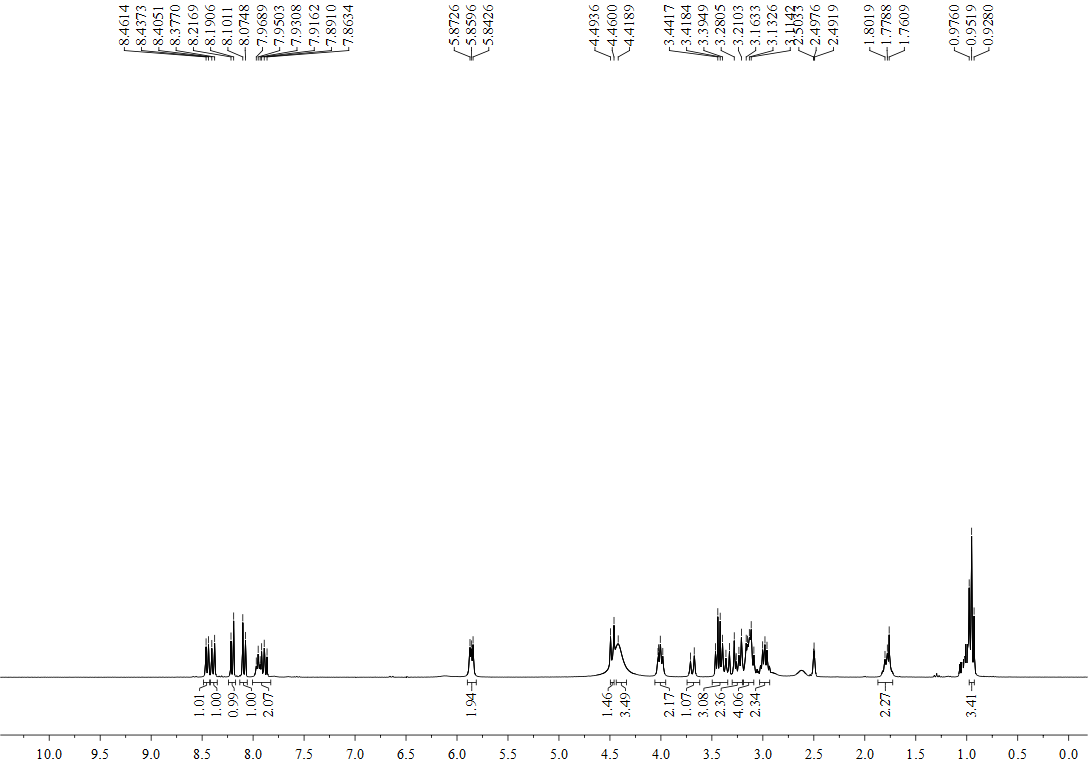


**^13^C NMR spectrum of 17d (300 MHz, DMSO-*d_6_*)**


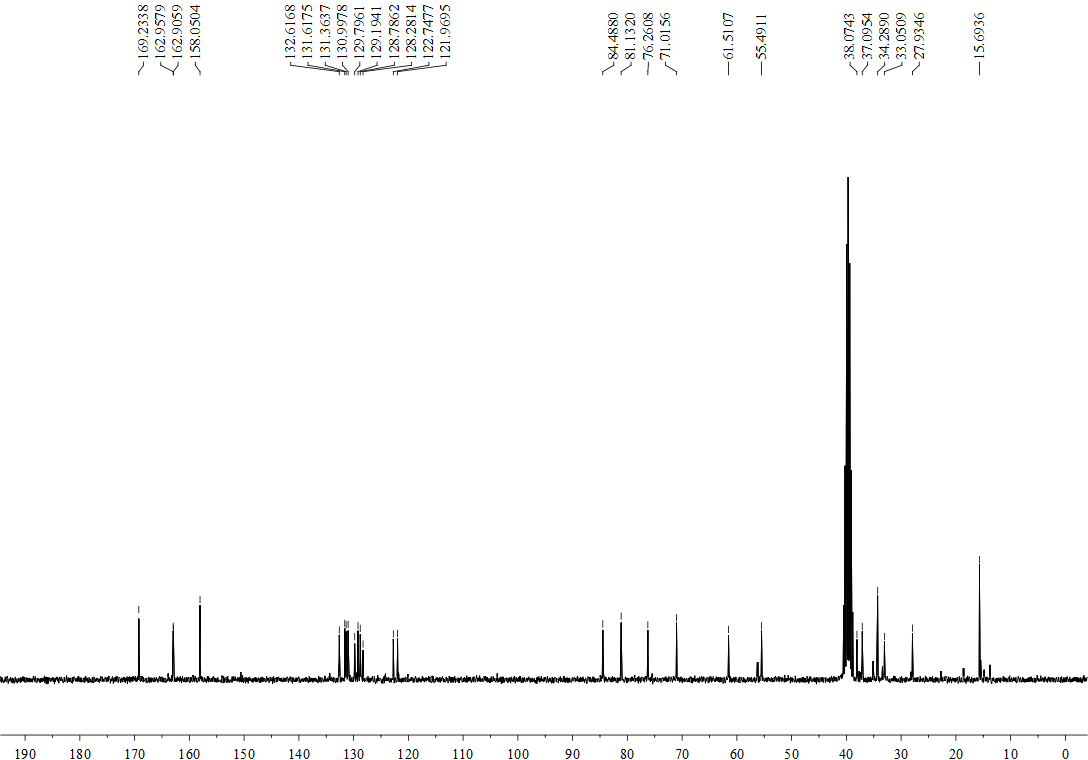

Supplement: Supplementary file 1 [file Table_1.docx]
